# Stratifin (SFN) regulates lung cancer progression via nucleating the Vps34‐BECN1‐TRAF6 complex for autophagy induction

**DOI:** 10.1002/ctm2.896

**Published:** 2022-06-08

**Authors:** Ji Young Kim, Mi‐Jeong Kim, Ji Su Lee, Juhee Son, Duk‐Hwan Kim, Joo Sang Lee, Soo‐Kyung Jeong, Eunyoung Chun, Ki‐Young Lee

**Affiliations:** ^1^ Department of Immunology and Samsung Biomedical Research Institute Sungkyunkwan University School of Medicine Suwon Republic of Korea; ^2^ Department of Molecular Cell Biology Sungkyunkwan University School of Medicine Suwon Republic of Korea; ^3^ Department of Precision Medicine Sungkyunkwan University School of Medicine Suwon Republic of Korea; ^4^ R&D Center, CHA Vaccine Institute Seongnam‐si Republic of Korea; ^5^ Samsung Medical Center Gangnam‐gu Republic of Korea; ^6^ Department of Health Sciences and Technology Samsung Advanced Institute for Health Sciences and Technology, Samsung Medical Center Sungkyunkwan University Gangnam‐gu Republic of Korea; ^7^ Single Cell Network Research Center Sungkyunkwan University School of Medicine Suwon Republic of Korea

Dear Editor,

1

Lung cancer progression is regulated by various extrinsic factors derived from tumour microenvironment as well as intrinsic factors.^1^ Recent studies have shown that toll‐like receptors (TLRs) are expressed in lung cancers, suggesting that TLRs may be implicated in lung cancer progression.^2^, ^3^ Although several studies have shown that stratifin (*SFN*, 14‐3‐3 sigma) facilitated lung cancer development and progression,^4^, ^5^, ^6^ the molecular and cellular mechanisms by which *SFN* is functionally involved in lung cancer progression, and the role of *SFN* in lung cancer progression in response to extrinsic stimulation, such as TLR agonist, are largely unknown. Here, we show that *SFN* expression is remarkably up‐regulated in lung cancer tissues through The Cancer Genome Atlas (TCGA) data and primary non‐small cell lung cancers (*n* = 31 of our cohort patients) analysis, and *SFN* positively regulates lung cancer progression through the autophagy induction by facilitating TRAF6‐ Vps34‐BECN1 complex in response to an extrinsic TLR4 agonist.

To get insight into the role of *SFN* in cancers, we first investigated whether the expression of *SFN* is associated with 33 different cancer types by analyzing TCGA datasets (http://gepia.cancer‐pku.cn/detail.php?gene=SFN, Figure 1A). The gene expression of *SFN* was significantly up‐regulated in 17 different tumour samples (Figure 1A, tumours are marked in red; Figure S1), whereas significantly down‐regulation was observed in four different tumour samples (Figure 1A, tumours are marked in green; Figure S2). In lung adenocarcinoma (LUAD) and lung squamous cell carcinoma (LUSC) tumour samples, *SFN* expression was significantly increased compared to those of paired normal tissues (Figure 1B,C). Interestingly, the percentage of survival was significantly lower in LUAD patients with high expression of SFN (Figure 1D, red vs. blue; *p* = .00065), whereas there was no significant difference was observed in LUSC patients (Figure 1E, red vs. blue; *p* = .74). Notably, microarray data analysis showed that the expression of *SFN* was increased in primary 23 LUAD patients and eight LUSC patients (Figure 1F), indicating that SFN may be functionally associated with LUAD. Given the above results, we examined whether the expression of SFN is associated with gene expressions related to cancer progression in primary LUAD patient tissues. We selected seven primary LUAD patients with high expression of SFN (Figure 1F, red bars), and the pathological classification was confirmed by H&E staining in matched lung normal tissue and lung tumour tissue, patient #26,^7^ #52, #13, #17, #51, #12 and #29 (Figure 2A). To sort out genes related to cancer progression, 500 up‐regulated genes or 500 down‐regulated genes were arranged based on the data of patient #26 with the most up‐regulation of SFN among the seven primary LUAD patients (Figure 2B, 500 up‐regulated genes; Table S1: Figure 2C, 500 down‐regulated genes; Table S2). Among the 500 down‐regulated genes, commonly up‐regulated genes (Figure 2D and Table S3) or down‐regulated genes (Figure 2E and Table S4) were further sorted out. Interestingly, 23 genes related to cancer proliferation (Figure 2F and Table S5), 17 genes related to cancer migration or invasion (Figure 2G and Table S6) and 12 genes related to cancer metastasis (Figure 2H and Table S7) were up‐regulated in the seven primary LUAD patient tumour tissues. Notably, 12, 10 and 8 genes were functionally associated with lung cancer proliferation, migration or invasion and metastasis, respectively (Figure 2F–H, red boxes; Table S5‐S7, red letters). Although the effect of SFN on cell cycle progression is controversial, a previous report has demonstrated that SFN in lung adenocarcinoma cells might have tissue‐specific functions and regulate cell cycle progression in a positive manner.^4^ Importantly, we found that 12 genes related to lung cancer proliferation have been reported to be involved in promoting cell cycle progression (Figure S3), indicating that *SFN* expression is associated with genes related to cell cycle progression in cancers. Moreover, 34 cancer suppressors genes were down‐regulated in tumour tissues of the seven primary LUAD patients (Figure 2I, Table S8), and 18 genes were functionally associated with lung cancer (Figure 2I, green box; Table S8, green letters). To verify the function of *SFN* in lung cancer progression, we generated *SFN*‐knockout (*SFN*KO) A549 human adenocarcinoma cells using the CRISPR cas9 gene editing method (Figure 2J, lane 2). The *SFN*KO A549 cells revealed marked inhibitions of cancer invasion and migration and colony formation and single‐cell mobility and proliferation as compared to those of control (Ctrl) A549 cells (Figure 2K, invasion; Figure 2L, migration; Figure 2M, anchorage‐dependent colony formation; Figure 2N, anchorage‐independent colony formation; Figure S4A,B, single‐cell mobility; Figure S5, cell proliferation: *SFN*KO A549 vs. Ctrl A549). Based on the TCGA and primary LUAD patient data analysis and the cancer progression assay of *SFN*KO lung cancer cells, we suggested that *SFN* might be functionally implicated in lung cancer progression.

**FIGURE 1:**
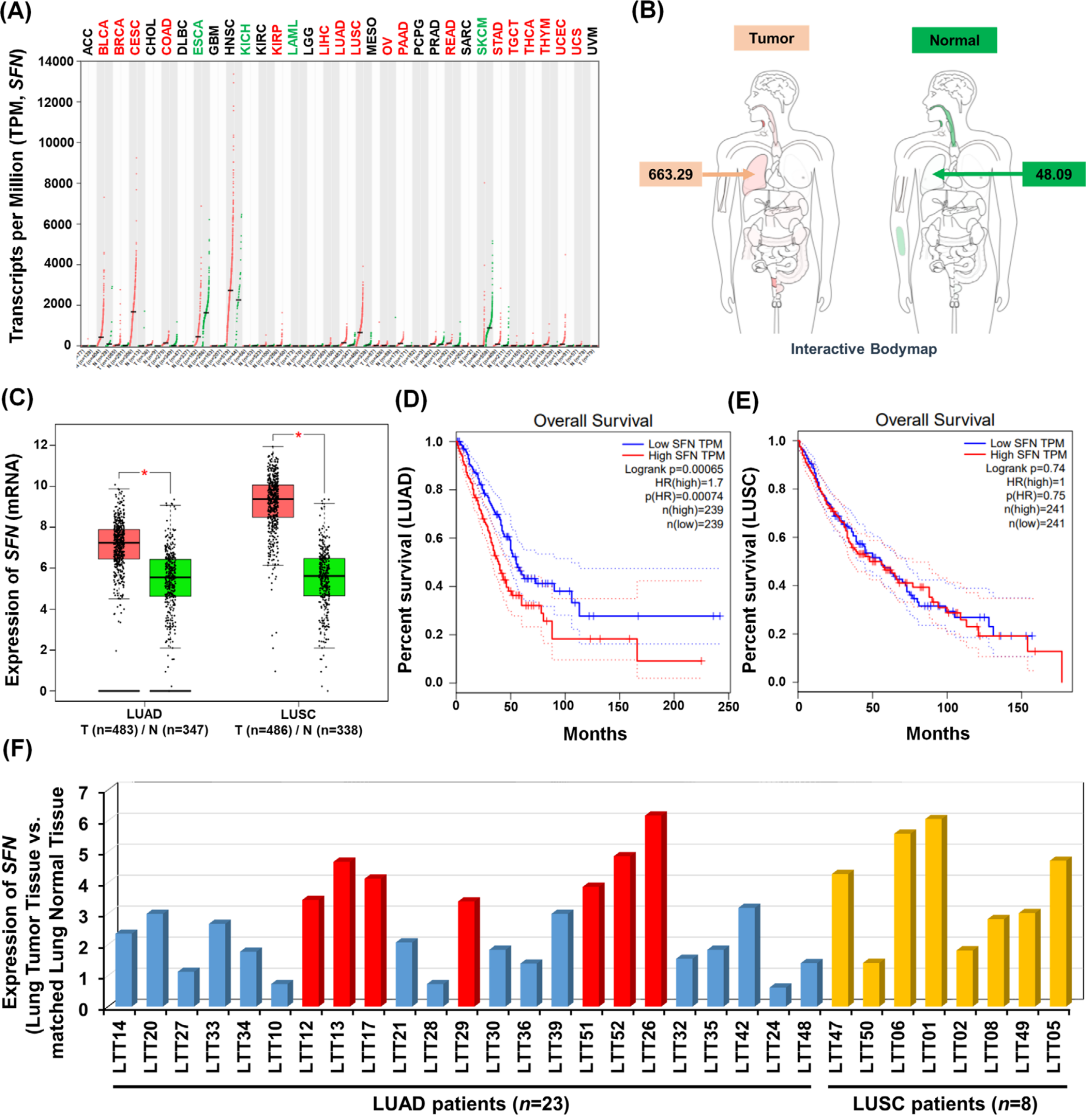
Stratifin *(SFN)* is up‐regulated in lung cancer. (A) The Cancer Genome Atlas (TCGA) data analysis of the expression of *SFN* in 33‐different cancer types (http://gepia.cancer‐pku.cn/detail.php?gene=SFN). (B) Interactive bodymap of a human lung in which the expression of *SFN* mRNA is significantly higher in tumours than in normal. (C) Scatter plots showing relative levels of *SFN* mRNA in normal and LUAD tumour or lung squamous cell carcinoma (LUSC) tumour tissues. Median expression levels in each group are indicated by horizontal lines. One‐way analysis of variance; *p < .05. (D and E) Overall survival and disease‐free survival curves for patient groups with high and low *SFN* expression levels in LUAD (D) or LUSC (E). The data were obtained from TCGA datasets. The significance of the differences between the two categories was determined by the Student *t* test (D, *p* = .00065; E, *p* = .74). (F) The expression of *SFN* in lung tumour tissues (LTTs) of primary 23 LUAD and eight LUSC patients. Microarray analysis was performed in lung tumour tissues (LTTs) and matched lung normal tissues (LNTs), as described in supporting information. The fold change of the expression of *SFN* is presented (LTTs vs. LNTs)

**FIGURE 2:**
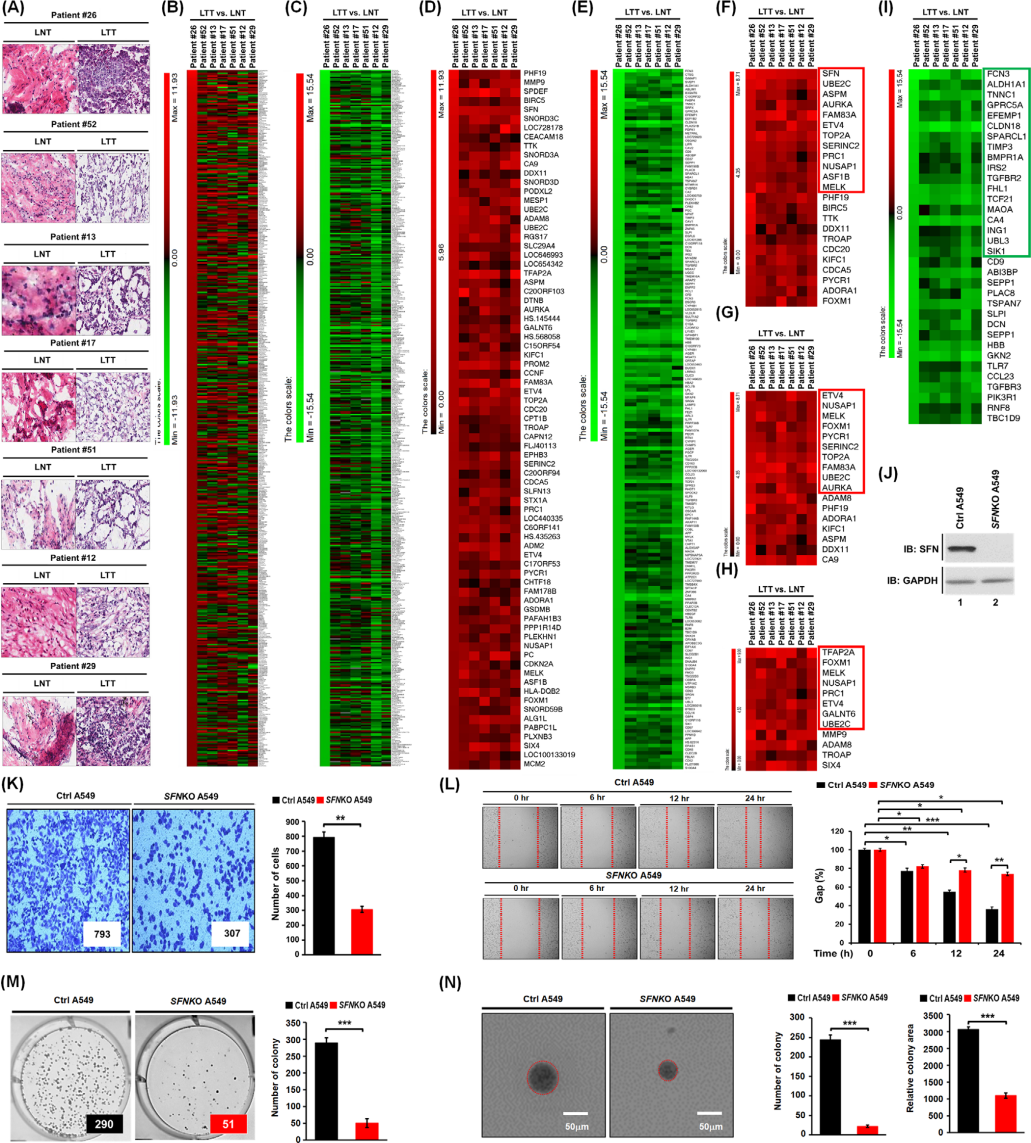
The Stratifin *(SFN)* expression is associated with genes related to lung cancer progression. (A) H&E (haematoxylin and eosin) staining was performed in primary lung tumour and matched normal tissues of LUAD patients with high expression of *SFN* in Figure 1F. (B–E) Microarray analysis was performed with tumour and matched normal tissues of patients #26, #52, #13, #17, #51, #12 and #29 with LUAD, as described in supporting information. Based on the results of patient #26 with the high expression of SFN among seven patients, 500 ea up‐regulated genes (B) and 500 ea down‐regulated genes (C) were sorted out and presented. Among the up‐regulated genes presented (B), commonly up‐regulated genes were sorted out and presented (D). Among the down‐regulated genes presented (C), commonly up‐regulated genes were sorted out and are presented (E). (F–I) Among the commonly up‐regulated genes, 23 genes related to cancer proliferation are represented, and 12 genes related to lung cancer proliferation were indicated as a red box (F). Seventeen genes related to cancer migration or invasion are represented, and 10 genes related to lung cancer migration or invasion were indicated as a red box (G). Twelve genes related to cancer metastasis are represented, and eight genes related to lung cancer metastasis are indicated as a red box (H). Among the commonly down‐regulated genes, 34 genes related to cancer suppressors are represented, and 18 genes related to lung cancer suppressors are indicated as a green box (I). (J) Generation of *SFN*‐knockout (*SFN*KO) A549 cells using CRISPR/Cas9 gene‐editing method. (K–N) Cancer invasion (K), migration (L), anchorage‐dependent colony‐forming (M) and anchorage‐independent colony‐forming (N) assay were performed with control (Ctrl) A549 and *SFN*KO A549 lung cancer cells. Results are presented as mean ± standard deviation (SD) of three independent experiments. The number of colonies was measured using Adobe Photoshop software (± SD, *n* = 3 plates). The size of the colony spheres was measured by ImageJ (± SD, *n* = 15 images). *p < .05, **p < .01 and ***p < .001

Further, we explored the molecular mechanism through which *SFN* is implicated in lung cancer progression. It has been reported that SFN accelerates lung cancer progression by regulating cell proliferation,^4^ and TLRs expressed in lung cancer promote the growth of cancer cells by inducing cell proliferation.^3^ Upon extrinsic TLR3/4 stimulations in lung cancer, additionally, autophagy was induced through the TRAF6‐BECN1 signaling axis, leading to enhanced cancer migration and invasion.^8^ In this study, we hypothesized that *SFN* regulates lung cancer progression through the autophagy induced by extrinsic TLR stimulation. SFN interacted with TRAF6 (Figure 3A, lane 4) or BECN1 (Figure 3B, lane 4). SFN interacted with TRAF6 wild type (WT), and TRAF6 110‐522 and TRAF6 260‐522 and TRAF6 349‐522 truncated mutants (Figure 3C). To verify the interaction, we performed an additional IP experiment with Flag‐TRAF6 1‐349 and Flag‐TRAF6 349‐522 truncated mutants. SFN interacted with Flag‐TRAF6 349‐522 but not with TRAF6 1‐349 (Figure S6), indicating that SFN interacts with the tumor necrosis factor receptor‐associated factor‐C terminus (TRAF‐C) domain of TRAF6 (Figure 3C, down). Additionally, SFN interacted with BECN1 WT, but not with BECN1 1‐269 and BECN1 1‐127 truncated mutants, indicating that SFN interacts with the C‐terminal domain of BECN1 (Figure 3D). These results suggest that SFN can nucleate the association of TRAF6‐BECN1 (Figure 3E). Importantly, the ubiquitination of BECN1 was markedly enhanced in the presence of SFN as compared to the absence of SFN (Figure 3F, lane 4 and 5 vs. lane 3), indicating that SFN nucleates the molecular association of TRAF6‐BECN1 and enhances the ubiquitination of BECN1 (Figure 3G). Since Vps34 interacted with the coiled‐coil domain of BECN1 and regulated autophagy,^9^ we further assessed whether SFN affects the association of Vps34‐BECN1 complex. Our results showed that Vps34 interacted with SFN (Figure 3H, lane 4) or BECN1 (Figure 3I, lane 4). Furthermore, the interaction between Vps34 and BECN1 was markedly enhanced in the presence of SFN as compared to the absence of SFN (Figure 3J, lane 4 or 5 vs. lane 3). Consistently, the endogenous interaction between BECN1 and Vps34 was significantly decreased in *SFN*KO A549 cells in the presence or absence of lipopolysaccharide (LPS) as compared to those of Ctrl A549 cells (Figure S7, lane 3 and 4 in *SFN*KO A549 vs. lane 1 and 2 in Ctrl A549). Interestingly, the ubiquitination of BECN1 was significantly enhanced in the presence of SFN and Vps34 compared to their absence (Figure 3K, lane 4 vs. lane 3). These results suggest that SFN facilitates the molecular associations of the TRAF6‐BECN1‐Vps34 complex through the interaction with BECN1 (Figure 3D) or Vps34 (Figure 3H), leading to the enhancement of the BECN1 ubiquitination (Figure 3L).

**FIGURE 3:**
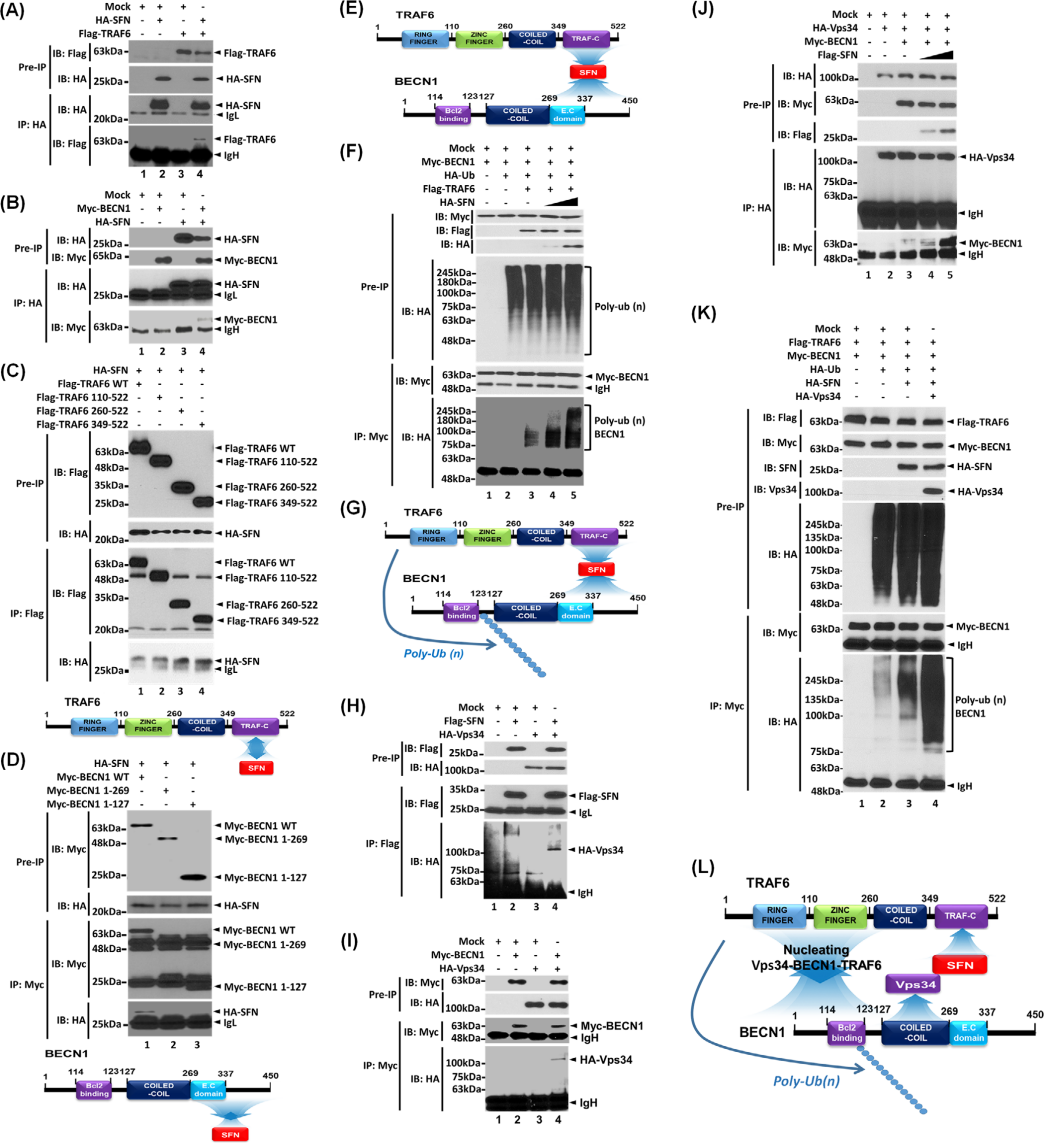
Stratifin *(SFN)* nucleates TRAF6‐Vps34‐BECN1 proteins and enhances BECN1 ubiquitination. (A and B) hemagglutinin (HA)‐SFN interacts with Flag‐TRAF6 (A, lane 4) or Myc‐BECN1 (B, lane 4). (C and D) HA‐SFN interacts with the tumor necrosis factor receptor‐associated factor‐C terminus (TRAF‐C) domain of TRAF6 (C) or with the C‐terminal region of BECN1 (D). (E) A schematic model of the association of TRAF6, SFN and BECN1. (F) SFN enhances BECN1 ubiquitination by TRAF6. (G) A schematic model of the BECN1 ubiquitination in a TRAF6‐SFN‐BECN1 complex. (H and I) HA‐Vps34 interacts with Flag‐SFN (H, lane 4) or Myc‐BECN1 (I, lane 4). (J) Flag‐SFN increases the molecular association of HA‐Vps34 and Myc‐BECN1. (K) HA‐SFN and HA‐Vps34 enhance the ubiquitination of BECN1. (L) A schematic model of how SFN nucleates TRAF6‐Vps34‐BECN1 proteins and enhances BECN1 ubiquitination

Autophagy regulated by the ubiquitination of BECN1 enhanced lung cancer migration and invasion in response to TLR3/4 stimulation.^8^ Upon TLR4 stimulation with LPS, the level of light chain 3‐II (LC3‐II) was significantly attenuated in *SFN*KO A549 cells as compared to Ctrl A549 (Figure 4A, lane 6 vs. lane 2). Moreover, cancer migration and invasion were markedly inhibited in *SFN*KO A549 cells treated with LPS, as compared to those of Ctrl A549 cells (Figure 4B,C, migration; Figure 4D,E, invasion: *SFN*KO A549 treated with LPS vs. Ctrl A549 treated with LPS). Consistently, single‐cell mobility was significantly attenuated in *SFN*KO A549 cells treated with LPS (Figure S8A,B, *SFN*KO A549 vs. Ctrl A549). Matrix metalloproteinase‐2 (MMP2) and interleukin‐6 (IL‐6), which are known to regulate cancer migration and invasion,^8^ were significantly down‐regulated in *SFN*KO A549 cells treated with LPS as compared to Ctrl A549 cells treated with LPS (Figure 4F, MMP2; Figure 4G, IL‐6). Furthermore, anchorage‐dependent or ‐independent colony formation revealed a significant decrease in *SFN*KO A549 cells‐treated LPS as compared to Ctrl A549 treated LPS (Figure 4H,I, anchorage‐dependent; Figure 4J,K, anchorage‐independent: *SFN*KO A549 cells treated LPS vs. Ctrl A549 cells treated LPS). Consistently, cell proliferation assay showed a significant decrease in *SFN*KO A549 cells in the presence of LPS (Figure S9, *SFN*KO A549 vs. Ctrl A549). In contrast, the co‐treatment of autophagy inhibitors, 3‐methyladenine (3‐MA) and chloroquine (CQ), markedly attenuated cancer migration and invasion, and colony formation induced by LPS stimulation (Figure 4B–E,H–K, LPS vs. LPS + 3‐MA or LPS + CQ). To verify these results, we performed rescue experiments with *SFN*KO A549 and *SFN*KO A549 transiently expressed with SFN (Figure S10). In *SFN*KO A549 cells transfected with hemagglutinin (HA)‐SFN (Figure S10A, lane 2), cancer migration and invasion were significantly elevated as compared to those of *SFN*KO A549 cells transfected with mock vector (Figure S10B–C, HA‐SFN‐expressed *SFN*KO A549 vs. *SFN*KO A549). These results suggest that *SFN* positively regulates cancer migration and invasion, and colony formation through the autophagy induction by TLR4.

**FIGURE 4:**
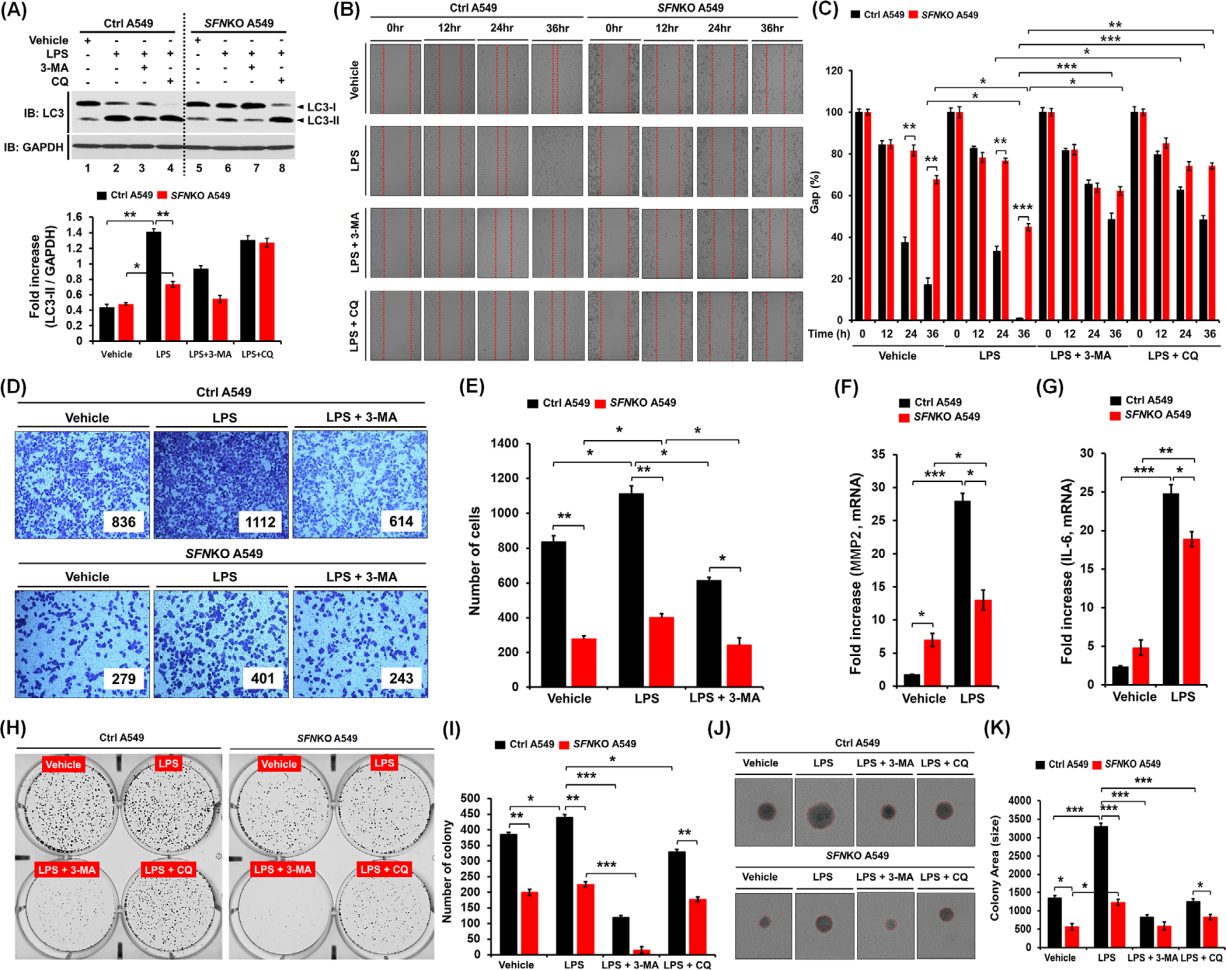
*SFN*KO A549 lung cancer cells exhibit the attenuation of autophagy induction and cancer progressive abilities induced by TLR4 stimulation. (A) Control (Ctrl) A549 and *SFN*KO A549 lung cancer cells were treated with a vehicle, lipopolysaccharide (LPS), 3‐methyladenine (3‐MA) and chloroquine (CQ), as indicated. LC3‐II levels were evaluated by western blotting assay. Results are presented as mean ± standard deviation (SD) of three independent experiments (down). (B and C) Cell migration assay was performed with Ctrl A549 and *SFN*KO A549 cells treated with vehicle, LPS, 3‐MA and CQ, as indicated (B). Results are presented as mean ± SD of three independent experiments (C). (D and E) Cell invasion assay was performed with Ctrl A549 and *SFN*KO A549 cells treated with vehicle, LPS and 3‐MA, as indicated (D). Results are presented as mean ± SD of three independent experiments (E). (F and G) Ctrl A549 and *SFN*KO A549 cells treated with vehicle and LPS, the mRNA levels of MMP2 (F) and interleukin‐6 (IL‐6) (G) were measured by quantitative real time polymerase chain reaction. Results are presented as mean ± SD (*n* = 3). (H and I) Anchorage‐dependent colony‐forming assay was performed with Ctrl A549 and *SFN*KO A549 cells treated with a vehicle, LPS, 3‐MA and CQ as indicated (H). The number of the colony was counted and presented as mean ± SD (*n* = 3) (I). (J and K) Anchorage‐independent colony‐forming assay was performed with Ctrl A549 and *SFN*KO A549 cells treated with vehicle, LPS, 3‐MA and CQ as indicated (J). Results are presented as mean ± SD of three independent experiments. The number of colonies was measured using Adobe Photoshop software (± SD, *n* = 3 plates). The size of the colony spheres was measured by ImageJ (± SD, *n* = 15 images). *p < .05, **p < .01, and ***p < .001

In summary, we demonstrated that *SFN* expression in lung cancer is clinically associated with poor patient survival and lung cancer progression, accompanying the up‐regulation of genes related to cancer progression and the down‐regulation of genes related to cancer suppression. Through the biochemical and cellular studies, we propose possible molecular and cellular mechanisms in which *SFN* is functionally implicated in lung cancer progression: (1) SFN nucleates the TRAF6‐BECN1‐Vps34 complex and enhances the ubiquitination of BECN1 and subsequently regulates autophagy, (2) upon extrinsic TLR4 stimulation, *SFN* enhances cancer progression including cancer migration and invasion, proliferation and colony formation through autophagy induction. Together, our clinically comparative results and functional investigations of SFN expression in lung cancer will potentially contribute to translational approaches for the development of lung cancer therapeutic agents.

## CONFLICT OF INTEREST

The authors declare that they have no competing interests.

## FUNDING INFORMATION

National Research Foundation of Korea (NRF) Grants, Korean Government, Grant Numbers: NRF‐2021R1F1A1049324 and NRF‐2021R1A2C1094478; Korea Basic Science Institute (National Research Facilities and Equipment Center) Grant, Ministry of Education, Grant Number: 2020R1A6C101A191; Ministry of Science ICT and Future Planning (MSIP), Korean Government, Grant Number: NRF‐2016R1A5A2945889

## Supporting information

Supporting information

Supporting information

Supporting information

Supporting information

Supporting information

Supporting information

Supporting information

Supporting information

Supporting information
