# Supplementary material for: Stratifin (SFN) regulates lung cancer progression via nucleating the Vps34‐BECN1‐TRAF6 complex for autophagy induction
Source: Clin Transl Med. 2022 Jun 8;12(6):e896. doi: 10.1002/ctm2.896 (PMC9174881; doi:10.1002/ctm2.896)
Supplement: Supplementary file 1 — Supporting information [file CTM2-12-e896-s008.docx]

**Supporting Information**

Stratifin (SFN) regulates lung cancer progression via nucleating the Vps34-BECN1-TRAF6 complex for autophagy induction

**Methods and Materials**

**Patient-derived Lung Tumor Tissues**

From 31 patients with primary Non-Small Cell Lung Cancer (NSCLC), tumor and matched normal tissues were obtained in accordance with the ethical principles stated in the Declaration of Helsinki. This study was approved by the Institutional Review Board (IRB) of the Samsung Medical Center (SMC) (IRB#: 2010-07-204), following procedures previously described.^1, 2^ We obtained written informed consent from each patient prior to surgery.

**Cells**

Human embryonic kidney (HEK) 293T cells (ATCC, CRL-11268) were cultured and maintained in Dulbecco’s modified Eagle’s medium (DMEM; Thermo Fisher Scientific, 11965092) with 10% fetal bovine serum (FBS). A549 cells (human lung cancer cell line; ATCC, CCL-185) were maintained in RPMI 1640 medium (Sigma Aldrich, 31800-022) supplemented with 10% FBS, penicillin (100 μg/mL), and streptomycin (100 μg/mL) in a 5% CO_2_ humidified atmosphere at 37°C.

**Antibodies and Reagents**

Anti-Myc (2276), anti-GAPDH (2118), and anti-LC3A/B (4108) were purchased from Cell Signaling Technology. Anti-Flag (SAB4200071) was purchased from Sigma-Aldrich. Anti-HA (ab18181), Anti-Vps34 (ab227861), Anti-BECN1 (ab62557), and Anti-SFN (ab151504) were purchased from Abcam. Lipopolysaccharide (LPS; serotype 0128: B12), chloroquine (CQ; C6628), dimethyl sulfoxide (DMSO; 472301), puromycin (P8833), paraformaldehyde (P6148), Triton X-100 (T8787), 3-methyladenine (3-MA; M9281), gentamicin (G1272), deoxycholate (D6750), and Dulbecco’s phosphate-buffered saline (DPBS; D8537) were purchased from Sigma-Aldrich. Lipofectamine 2000 (11668019) was purchased from Thermo Fisher Scientific.

**Plasmid Constructs**

Flag-TRAF6 (21624), HA-14-3-3 sigma (SFN, 11946), HA-Vps34 (86749), and Flag-BECN1 (24388) plasmids were purchased from Addgene. HA-tagged Ub plasmids were obtained from Dr. J. H. Shim (University of Massachusetts Medical School, USA). Using the HA-SFN or Flag-BECN1 plasmid, full-length Flag-SFN, and Myc-BECN1 constructs were cloned into a pCMV-3Tag-7 (Agilent technologies, 240202) or a pCMV-3Tag-6 vector (Agilent technologies, 240200). Truncated mutants of Flag-TRAF6 and Myc-BECN1 were generated as previously described.^3, 4^

**Generation of SFN-knockout (SFNKO) Cell Line with CRISPR/Cas9**

Guide RNA sequences for CRISPR/Cas9 were designed on the CRISPR design website (http://crispr.mit.edu/), provided by the Feng Zhang Lab. Insert oligonucleotides for human SFN gRNA were 5’-CACCGGGCCGGGGACGCCGAGAGCC-3’ / 3’- CCCGGCCCCTGCGGCTCTCGGCAAA-5’. The complementary oligonucleotides for guide RNAs (gRNAs) were annealed, and cloned into lenti CRISPR v2 vector (Addgene plasmid, Ca#52961). A549 cells were transfected with lenti CRISPR v2/gRNA using Lipofectamine 2000, according to the manufacturer’s instructions. Two days after transfection, the cells were treated with 2 μg/ml of puromycin for three days. After two weeks, colonies were isolated into 96-well plates, and the expression levels of SFN were analyzed with western blot.

**Western Blotting (WB) and Immunoprecipitation (IP) Assays**

WB and IP assays were performed as previously described.^3-9^. Briefly, HEK-293T cells were seeded into 6-well plates, transfected, and treated as described in the text and Figures. These cells were then incubated for 38 to 48 hr. After collecting the cells, cell lysates were prepared and immunoprecipitated with anti-Myc, anti-HA, or anti-Flag antibodies. IP complexes were separated by sodium dodecyl sulfate-polyacrylamide gel electrophoresis (SDS-PAGE, 6 – 10%) and immune-probed with different antibodies as indicated in the text. For endogenous IP assay, Control (Ctrl) A549 and *SFN*KO A549 cells were treated with or without LPS (10 μg/mL) for 60 min, and cell lysates were prepared and immunoprecipitated with anti-Vps34 antibody. IP complexes were separated by sodium dodecyl sulfate-polyacrylamide gel electrophoresis (SDS-PAGE, 6 – 10%) and immune-probed with different antibodies as indicated in the text. For ubiquitination assay, mock vector, Myc-BECN1, HA-Ub, Flag-TRAF6, HA-SFN, and HA-Vps34 vectors were transfected into HEK293T cells as described in the text and Figures. Cell lysates were immunoprecipitated with anti-Myc antibody and probed with different antibodies as indicated in the text and Figures. Ctrl A549 and *SFN*KO A549 cells were treated with or without a vehicle or CQ (10 μM) or 3-MA (5 mM) in the presence or absence of LPS (10 μg/mL) for 6 hr. Cell lysates were immunoblotted with an anti-LC3A/B antibody and anti-GAPDH (as a loading control). For rescue experiments, *SFN*KO A549 cells were transiently transfected with mock or HA-SFN, and confirmed with anti-HA and anti-GAPDH antibodies.

**Reverse Transcription-Quantitative Polymerase Chain Reaction (qRT-PCR) Analysis**

Control (Ctrl) A549 and *SFN*KO A549 cells were treated with or without 10 μg/mL LPS for 6 hr. qRT-PCR analysis was performed. Briefly, after extracting total RNA using an RNA isolation kit (A&A Biotechnology, Gdynia, Poland) according to the manufacturer’s protocol, cDNA was prepared by RT using AmfiRivert II cDNA Synthesis Master Mix (genDEPOT, R550) according to the manufacturer’s protocol. Primers hIL-6 (PPH 00560C) and hMMP2 (PPH 00151B) were purchased from Qiagen, Inc. (Chatsworth, CA, USA). Fluorescence detection was performed using an ABI PRISM 7700 Sequence Detector (PerkinElmer; Applied Biosystems; Thermo Fisher Scientific, Inc.). The mRNA expression level was calculated and normalized to the level of GAPDH.

**Wound-Healing Migration Assay**

A wound-healing migration assay was performed following previous protocols.^3, 6, 10, 11^ Briefly, Control (Ctrl) A549 and *SFN*KO A549 cells were seeded into 12-well plates and cultured to reach confluence. Cell monolayers were gently scratched and washed with a culture medium. After floating cells and debris were removed, cells attached to the culture plates were treated with a vehicle (DMSO), 3-MA (5mM), or CQ (10 μM) in the presence or absence of LPS (10 μg/mL). Cell images were captured after culturing for different time periods as indicated in each experiment. For rescue experiment, *SFN*KO A549 cells were transfected with mock or HA-SFN vector, and cells were seeded into 12-well plates and cultured to reach confluence. Cell monolayers were gently scratched and washed with a culture medium. After floating cells and debris were removed, cells attached to the culture plates were treated with a vehicle (DMSO) or 3-MA (5mM) in the presence or absence of LPS (10 μg/mL). Cell images were captured after culturing for different time periods as indicated in each experiment.

**Transwell Invasion Assay**

Transwell invasion assay was performed following previous protocols.^3, 6, 10, 11^ Briefly, Control (Ctrl) A549 and *SFN*KO A549 cells were suspended in culture medium (250 μL) without FBS. Cells were then added to the upper compartment of a 24-well Transwell® chamber containing a polycarbonate filter with 8-mm pores and coated with 60 mL of Matrigel (Sigma Aldrich, E1270; 1:9 dilution). A culture medium with 10% FBS was added to the lower chamber. After incubating for 24 hr, the cells in the upper compartment were removed, washed with PBS, and fixed. Invaded cells were stained with 4,6-diamidino-2-phenylindole (Sigma-Aldrich, D9542) and quantified by counting the number of fluorescent cells. For rescue experiment, *SFN*KO A549 cells were transfected with mock or HA-SFN vector and were suspended in culture medium (250 μL) without FBS. Cells were then added to the upper compartment of a 24-well Transwell® chamber containing a polycarbonate filter with 8-mm pores and coated with 60 mL of Matrigel (Sigma Aldrich, E1270; 1:9 dilution). A culture medium with 10% FBS was added to the lower chamber. After incubating for 24 hr, the cells in the upper compartment were removed, washed with PBS, and fixed. Invaded cells were stained with 4,6-diamidino-2-phenylindole (Sigma-Aldrich, D9542) and quantified by counting the number of fluorescent cells.

**Single-cell Migration Assay**

Single-cell migration assay was performed following the protocols provided by the Bio-protocol (www.bio-protocol.org/e3586). Briefly, Ctrl A549 and *SFN*KO A549 cells were seeded into a 6-well culture plate, treated with a vehicle (DMSO) and 3-MA (5mM) in the presence or absence of LPS (10 μg/mL), and allowed to attach overnight at 37 °C and 5% CO_2_. 5-10 single cells were selected from multiple culture fields, and time-lapse imaging analysis was performed at different times using a phase-contrast microscope (Olympus IX71 inverted microscope). Data analysis was performed following protocols provided by the Bio-protocol (www.bio-protocol.org/e3586).

**Anchorage-independent Soft Agar Colony Formation Assay**

Anchorage-independent soft agar colony formation assay was performed following previous protocols.^6,12^ Briefly, Ctrl A549 and *SFN*KO A549 cells (1.0 × 10^4^ cells per well) mixed with 0.3% Difco Noble Agar (BD) in complete medium were plated on bottom of a 0.5% agar layer in a 6-well plate with a complete medium. Growth medium (1.5 mL) with a vehicle (DMSO), LPS (10 μg/mL), 3-MA (5mM), or CQ (10 μM) was added on top of the layer and the cells were incubated at 37 °C for 4 weeks.

**Colony Formation Assay**

The ability of a single cell to grow into a colony was passed through a colony formation assay as previously described.^6, 13, 14^ Ctrl A549 and *SFN*KO A549 cells were harvested with trypsin-EDTA and resuspended in a singular form. The 1 × 10^3^ cells (per well) were plated in a 6-well plate and treated with a vehicle (DMSO), 3-MA (5mM), and CQ (10 μM) in the presence or absence of LPS (10 μg/mL). After incubation for ~9 days, colonies were stained with 0.5% crystal violet (Sigma) for 30 min at room temperature.

**MTT Assay**

Ctrl A549 and *SFN*KO A549 cells were seeded in a 96-well culture plate at a density of 1000 cells/well, treated with vehicle (DMSO, < 0.1%) and LPS (10 μg/mL), and grown in a culture medium supplemented with 10% FBS for different times. Cells viability was measured using an MTT reagent (Sigma) dissolved in PBS (5 mg/ml). On the day when the measurements were taken, the medium was carefully replaced on fresh RPMI + 10% FBS with diluted MTT (1:10, 10% MTT) and incubated for 3 hr at 37 °C. After removing the incubation medium, formazan crystals were dissolved in a 200 μl solution of DMSO. MTT reduction was quantified by measuring the light absorbance at 570 nm using the ELx800 absorbance microplate reader (BioTek Instruments, VT, USA). Each test was repeated at least four times in the quadruple.

**Microarray Analysis**

Microarray analysis was performed as previously described.^4, 15-17^ From tumor and matched normal tissues of 31 patients with NSCLC, total RNAs were extracted with Trizol (Thermo Fisher Scientific, 15596026) and purified using RNeasy columns (Qiagen, 74106) according to each manufacturer’s protocol. Microarray analysis was performed and analyzed as previously described.^4, 15-17^

**Hematoxylin and Eosin (H&E) Stain**

Lung tumor and matched normal tissues were embedded in paraffin. The paraffin-embedded tissue sections were stained with hematoxylin and eosin (H&E) as previously described.^5^

**TCGA Data Analysis**

The expression of SFN in human cancers was analyzed using TCGA data (GEPIA, gene expression profiling interactive analysis; http://gepia.cancer-pku.cn/ and http://gepia.cancer-pku.cn/detail.php?gene=SFN).

**Statistical Analysis**

All in vitro data are expressed as mean ± SD of triplicate samples or three independent experiments. Statistical significance was analyzed with ANOVA or Student’s t-test using GraphPad Prism 5.0 (GraphPad Software, San Diego, CA, USA).

**References**

1 Kim MJ, Min Y, Jeong SK, et al. USP15 negatively regulates lung cancer progression through the TRAF6-BECN1 signaling axis for autophagy induction. *Cell Death Dis.* 2022; **13**(4):348.

2 Kim Y, Lee BB, Kim D, et al. Clinicopathological Significance of RUNX1 in Non-Small Cell Lung Cancer. *J Clin Med.* 2020;9(6):1694.

3. Min Y, Kim MJ, Lee S, Chun E, Lee KY. Inhibition of TRAF6 ubiquitin-ligase activity by PRDX1 leads to inhibition of NFKB activation and autophagy activation. *Autophagy.* 2018; **14**(8):1347-1358.

4. Kim MJ, Min Y, Son J, et al. AMPKα1 Regulates Lung and Breast Cancer Progression by Regulating TLR4-Mediated TRAF6-BECN1 Signaling Axis. *Cancers (Basel).* 2020; **12**(11):3289.

5. Kim MJ, Choi B, Kim JY, et al. USP8 regulates liver cancer progression via the inhibition of TRAF6-mediated signal for NF-κB activation and autophagy induction by TLR4. *Transl Oncol.* 2022; **15**(1):101250.

6 Son J, Kim MJ, Lee JS, Kim JY, Chun E, Lee KY. Hepatitis B virus X Protein Promotes Liver Cancer Progression through Autophagy Induction in Response to TLR4 Stimulation. *Immune Netw.* 2021; **21**(5):e37.

7 Min Y, Lee S, Kim MJ, Chun E, Lee KY. Ubiquitin-Specific Protease 14 Negatively Regulates Toll-Like Receptor 4-Mediated Signaling and Autophagy Induction by Inhibiting Ubiquitination of TAK1-Binding Protein 2 and Beclin 1. *Front Immunol.* 2017; **8**:1827.

8 Mi Wi S, Park J, Shim JH, Chun E, Lee KY. Ubiquitination of ECSIT is crucial for the activation of p65/p50 NF-κBs in Toll-like receptor 4 signaling. *Mol Biol Cell.* 2015; **26**(1):151-160.

9 Wi SM, Moon G, Kim J, et al. TAK1-ECSIT-TRAF6 complex plays a key role in the TLR4 signal to activate NF-κB. *J Biol Chem.* 2014; **289**(51):35205-35214.

10 Kim MJ, Min Y, Im JS, Son J, Lee JS, Lee KY. p62 is Negatively Implicated in the TRAF6-BECN1 Signaling Axis for Autophagy Activation and Cancer Progression by Toll-Like Receptor 4 (TLR4). *Cells.* 2020; **9**(5):1142.

11 Kim MJ, Min Y, Shim JH, Chun E, Lee KY. CRBN Is a Negative Regulator of Bactericidal Activity and Autophagy Activation Through Inhibiting the Ubiquitination of ECSIT and BECN1. *Front Immunol.* 2019; **10**:2203.

12 Borowicz S, Van Scoyk M, Avasarala S, et al. The soft agar colony formation assay. *J Vis Exp.* 2014; (92):e51998.

13 Park S, Ha YN, Dezhbord M, et al. Suppression of Hepatocyte Nuclear Factor 4 α by Long-term Infection of Hepatitis B Virus Contributes to Tumor Cell Proliferation. *Int J Mol Sci.* 2020; **21**(3):948.

14 Franken NA, Rodermond HM, Stap J, Haveman J, van Bree C. Clonogenic assay of cells in vitro. *Nat Protoc.* 2006; **1**(5):2315-2319.

15 Min Y, Wi SM, Shin D, Chun E, Lee KY. Peroxiredoxin-6 Negatively Regulates Bactericidal Activity and NF-κB Activity by Interrupting TRAF6-ECSIT Complex. *Front Cell Infect Microbiol.* 2017; **7**:94.

16 Kim SY, Jeong S, Jung E, et al. AMP-activated protein kinase-α1 as an activating kinase of TGF-β-activated kinase 1 has a key role in inflammatory signals. *Cell Death Dis.* 2012; **3**(7):e357.

17 Min Y, Wi SM, Kang JA, et al. Cereblon negatively regulates TLR4 signaling through the attenuation of ubiquitination of TRAF6. *Cell Death Dis.* 2016; **7**(7):e2313.

**Supplementary figures and figure legends**

**
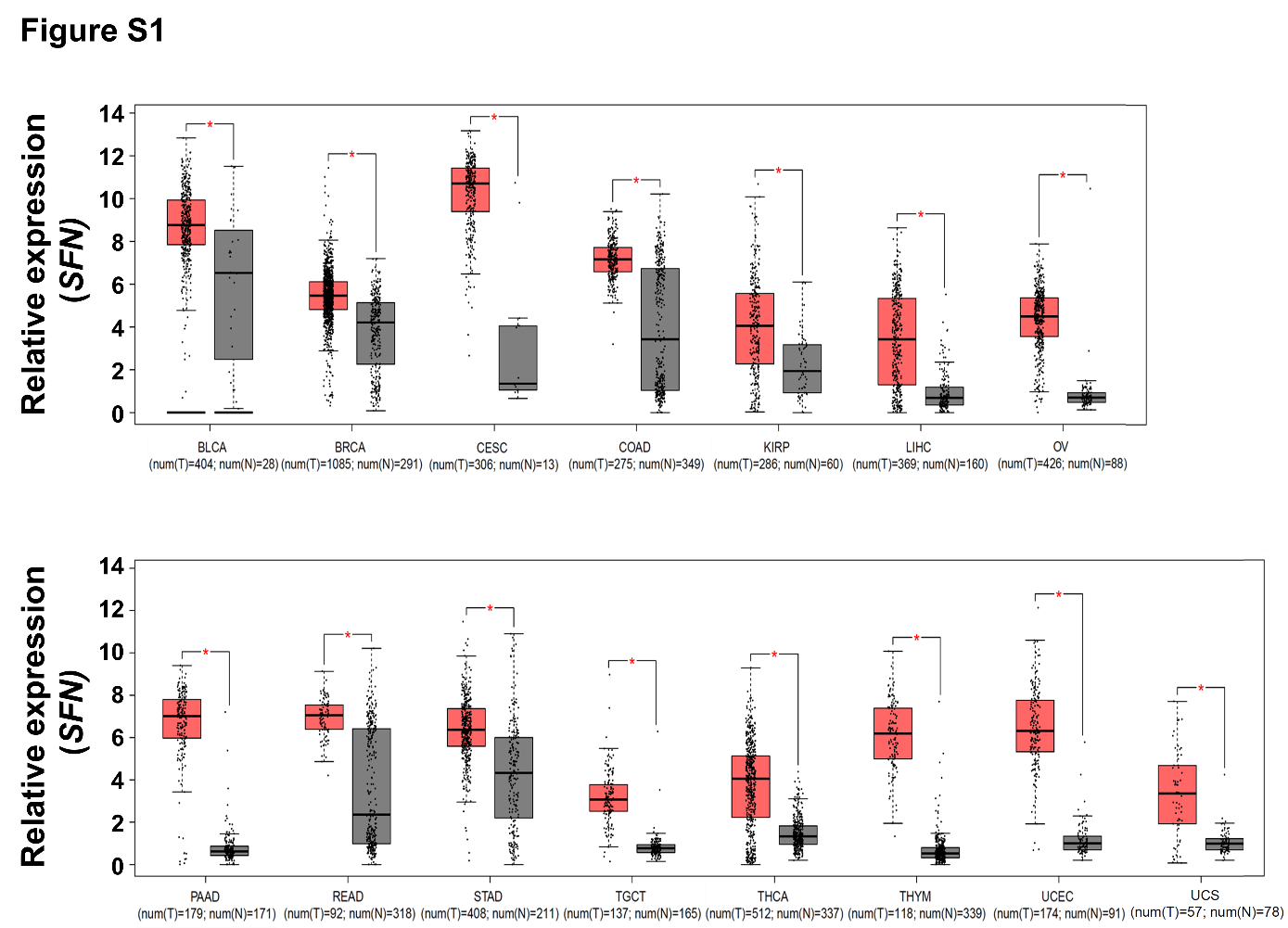
**

**Figure S1. The expression of SFN is upregulated in 15 different cancer types**. The expression of SFN was analyzed in 15-different cancer types using The Cancer Genome Atlas (TCGA) data (<http://gepia.cancer-pku.cn/detail.php?gene=SFN>), as indicated. Median expression levels in each group are indicated by horizontal lines.

**
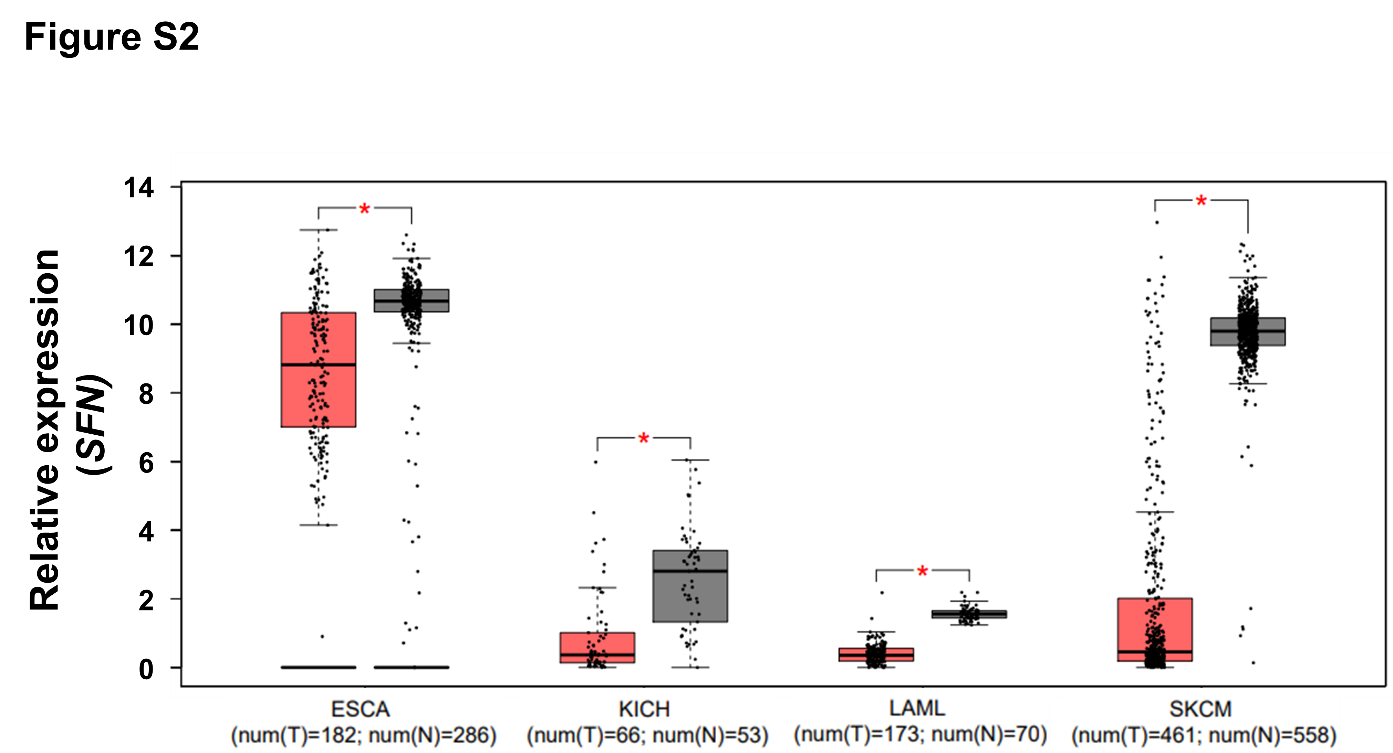
**

**Figure S2. The expression of SFN is downregulated in 4-different cancer types**. The expression of SFN was analyzed in 4-different cancer types using The Cancer Genome Atlas (TCGA) data (<http://gepia.cancer-pku.cn/detail.php?gene=SFN>), as indicated. Median expression levels in each group are indicated by horizontal lines.


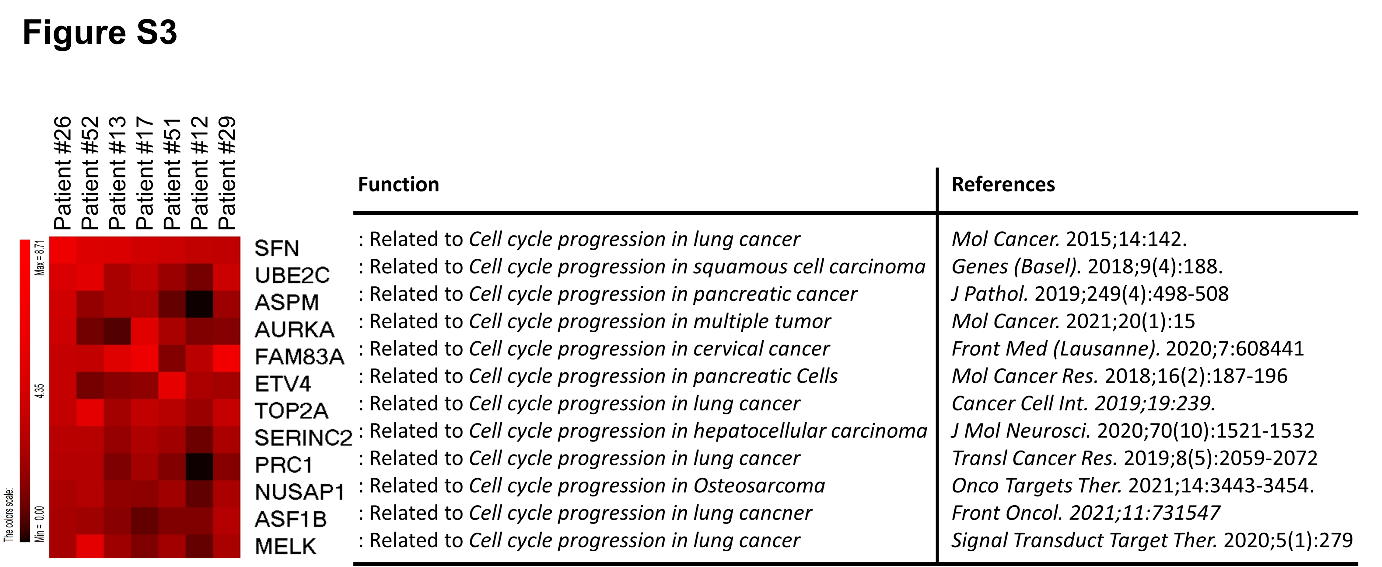


**Figure S3. 12 genes related to lung cancer proliferation in 7 primary LUAD patients with high expression of SFN are associated with cell cycle progression in cancers**. Through the PubMed article search, the function of 12 genes related to lung cancer proliferation (left) were identified (right, function and related references)

**
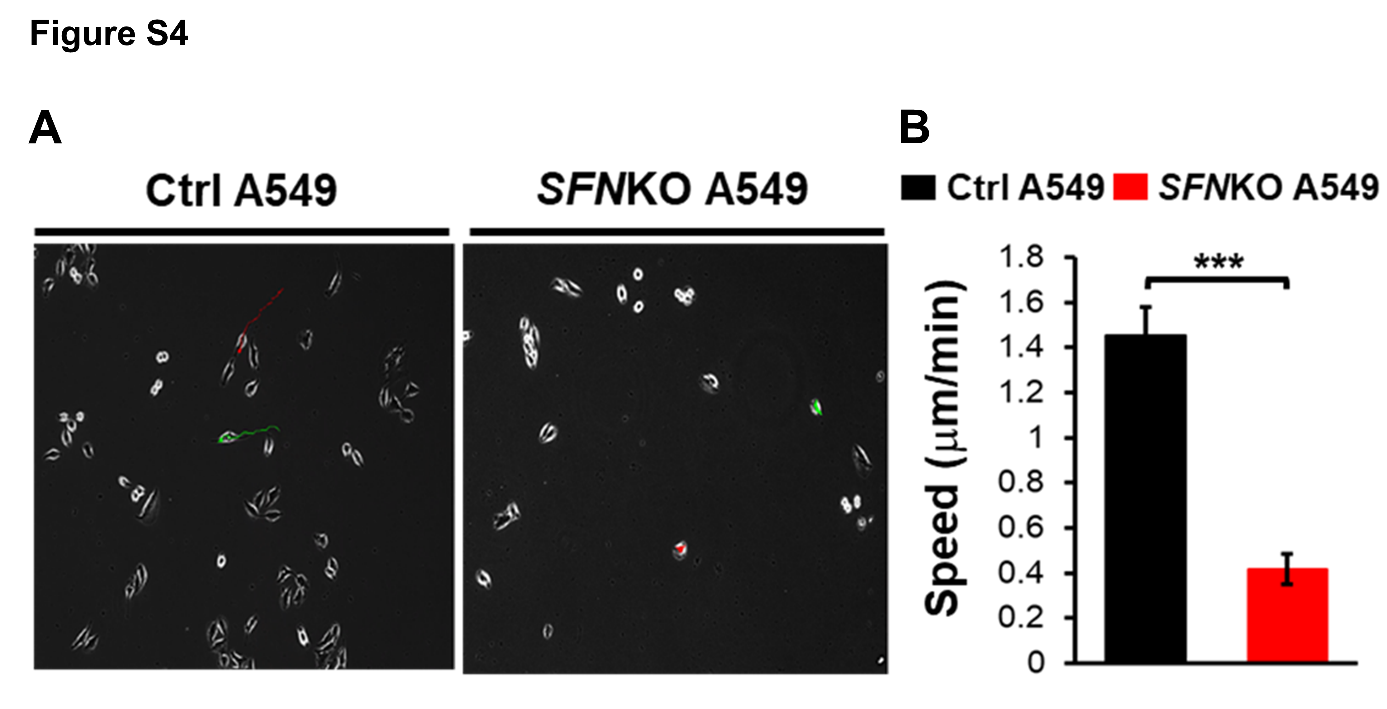
**

**Figure S4. Decrease of single-cell mobility in *SFN*KO A549 lung cancer cells**. (**A** and **B**) Control (Ctrl) A549 and *SFN*KO A549 cells were seeded in a 6-well culture plate and allowed to attach overnight at 37 °C and 5% CO_2_. 5-10 single cells were selected in multiple culture fields, and time-lapse imaging analysis was performed at different time periods using a phase-contrast microscope (**A**). Data analysis was performed following protocols provided by the Bio-protocol ([www.bio-protocol.org/e3586](http://www.bio-protocol.org/e3586)) (**B**). ***p<0.001.

**
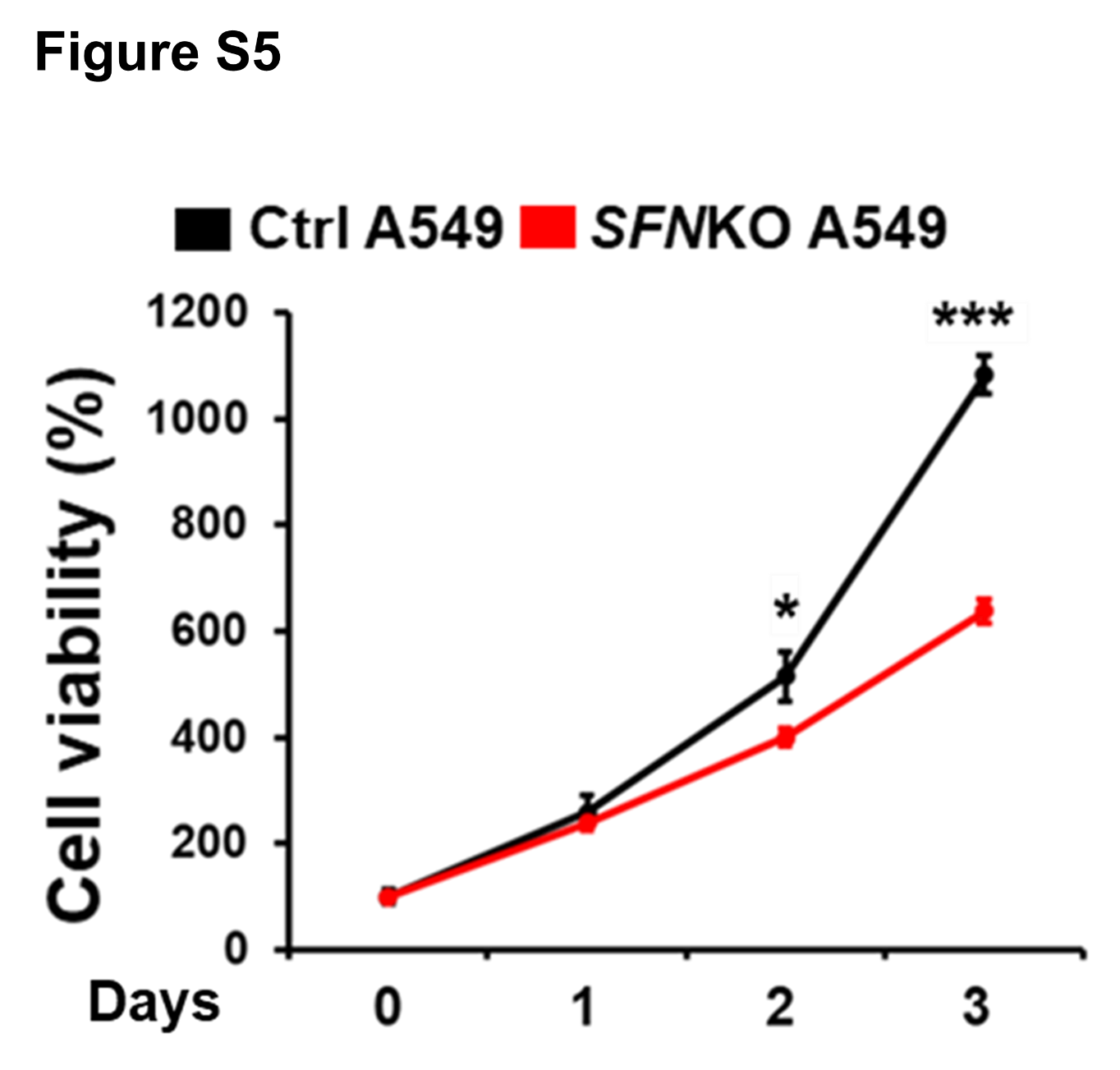
**

**Figure S5. Cell proliferation assay for Ctrl A549 and *SFN*KO A549 cells**. Ctrl A549 and *SFN*KO A549 cells were seeded into a 96-well culture plate at a density of 1000 cells/well and grown in a culture medium supplemented with 10% FBS. Cells viability was measured using an MTT reagent dissolved in PBS (5 mg/ml). On the day when the measurements were taken, the medium was carefully replaced on fresh RPMI + 10% FBS with diluted MTT (1:10, 10% MTT) and incubated. After removing the incubation medium, formazan crystals were dissolved in a 200 μl solution of DMSO. MTT reduction was quantified by measuring the light absorbance at 570 nm using the ELx800 absorbance microplate reader (BioTek Instruments, VT, USA). Each test was repeated at least four times in quadruples. *p<0.05 and ***p<0.001.


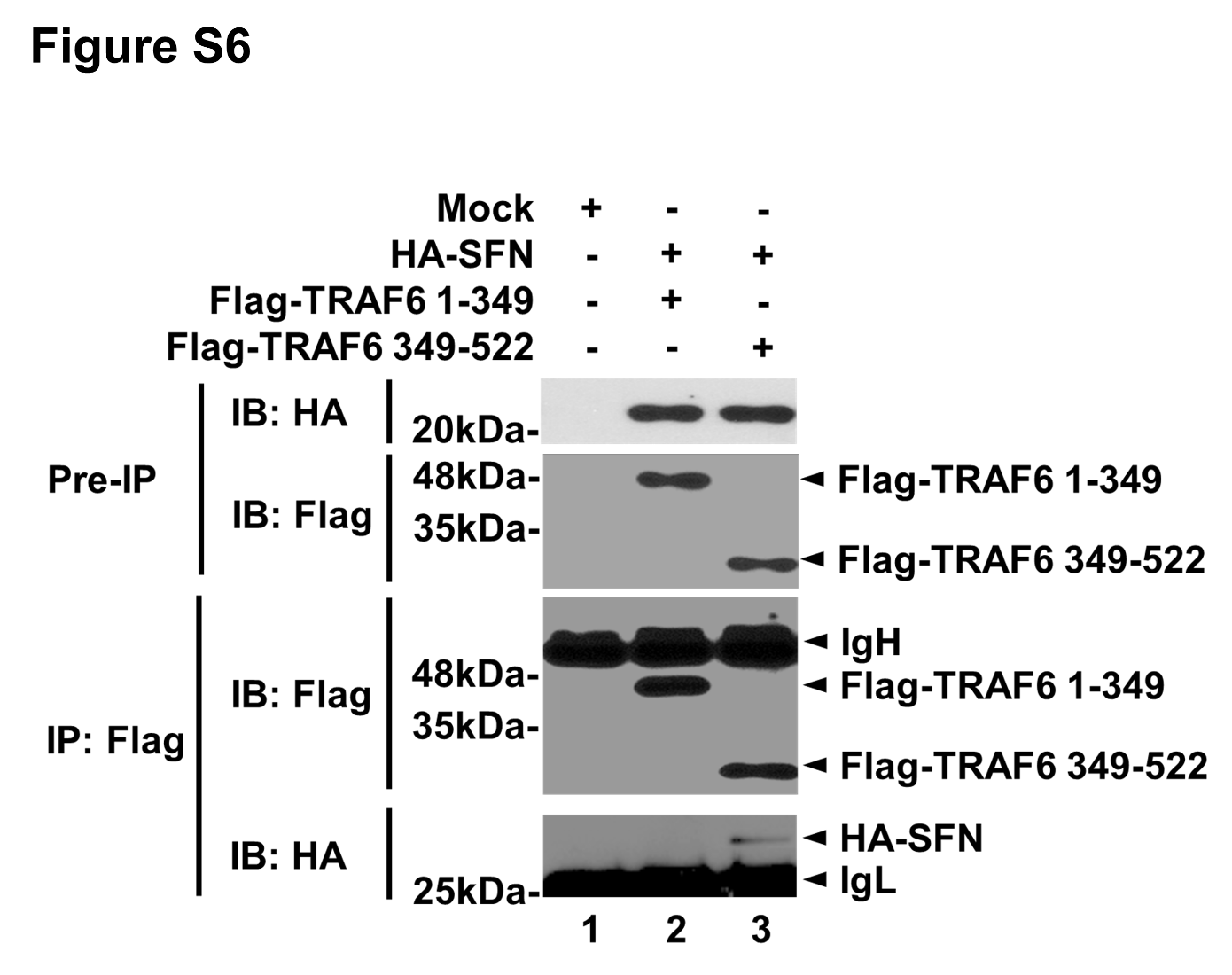


**Figure S6. HA-SFN interacts with Flag-TRAF6 349-522, but not with Flag-TRAF6 1-349**. HEK-293T cells were transfected with mock, HA-SFN, Flag-TRAF6 1-349, or Flag-TRAF6 349-522, as indicated. These cells were then incubated for 38 to 48 hr. After collecting the cells, cell lysates were prepared and immunoprecipitated with anti-Flag antibody. IP complexes were separated by sodium dodecyl sulfate-polyacrylamide gel electrophoresis (SDS-PAGE, 6 – 10%) and immune-probed with anti-HA or anti-Flag antibodies.


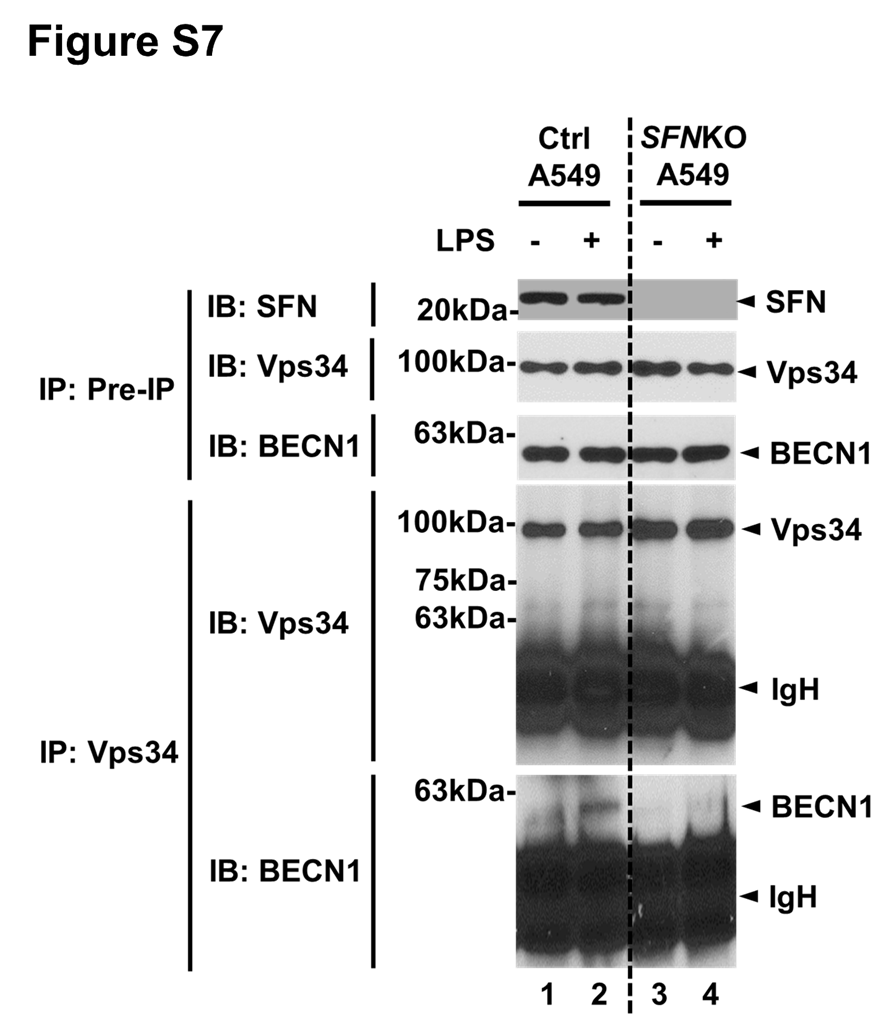


**Figure S7. The endogenous interaction between BECN1 and Vps34 in Ctrl A549 or *SFN*KO A549 cells**. Ctrl A549 and *SFN*KO A549 cells were treated with or without LPS (10 μg/mL) for 60 min. Cell lysates were prepared and immunoprecipitated with anti-Vps34 antibody. IP complexes were separated by sodium dodecyl sulfate-polyacrylamide gel electrophoresis (SDS-PAGE, 6 – 10%) and immune-probed with anti-SFN, or anti-Vps34, or anti-BECN1 antibodies.

**
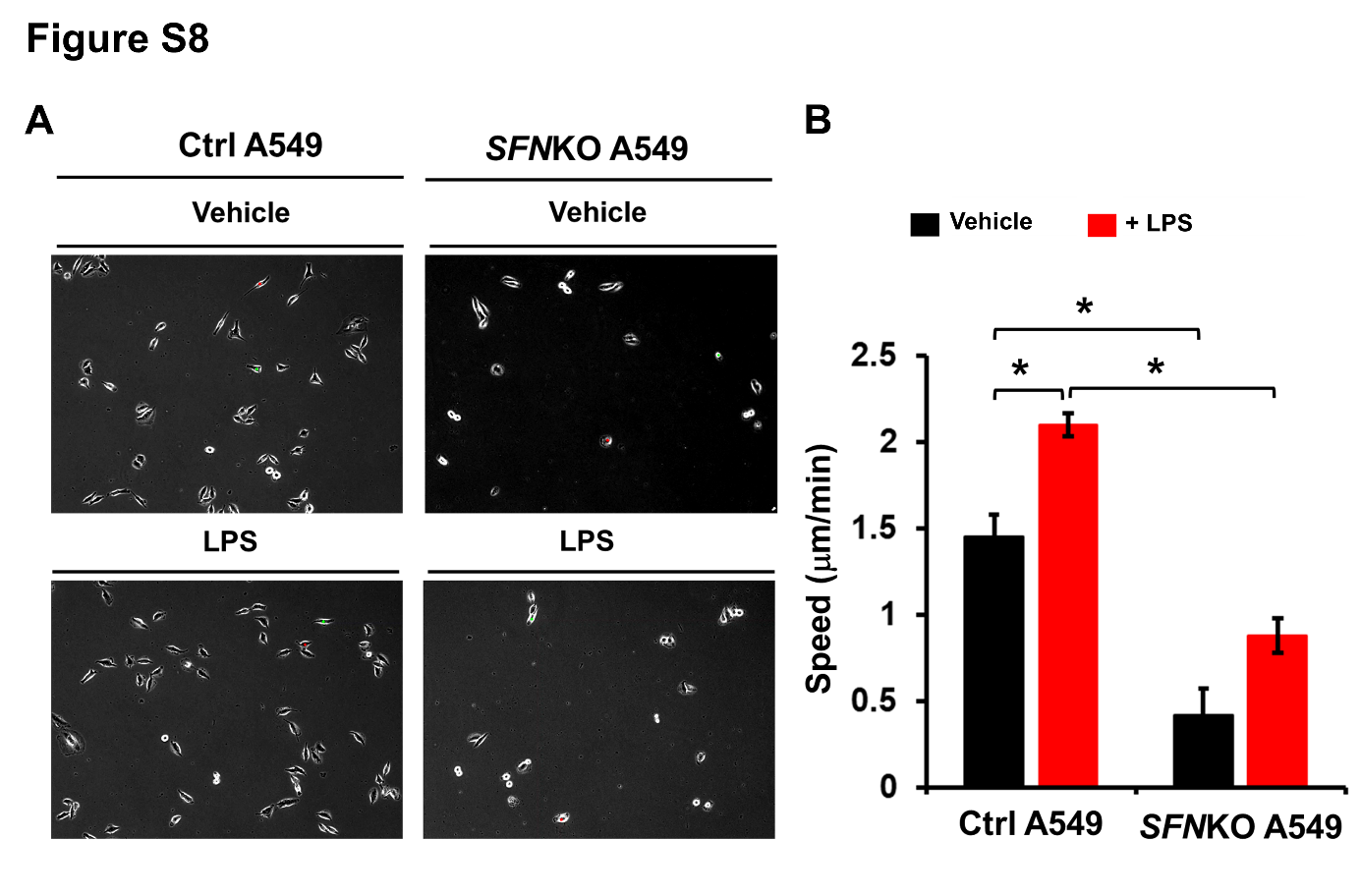
**

**Figure S8. *SFN*KO A549 lung cancer cells exhibit a decrease in cell mobility induced by TLR4 stimulation**. (**A** and **B**) Control (Ctrl) A549 and *SFN*KO A549 cells were seeded into a 6-well culture plate, treated with a vehicle (DMSO, < 0.1%) and LPS (10 μg/mL), and allowed to attach overnight at 37 °C and 5% CO_2_. 5-10 single cells were selected from multiple culture fields, and time-lapse imaging analysis was performed at different times using a phase-contrast microscope (**A**). Data analysis was performed following protocols provided by the Bio-protocol ([www.bio-protocol.org/e3586](http://www.bio-protocol.org/e3586)) (**B**). *p<0.05.


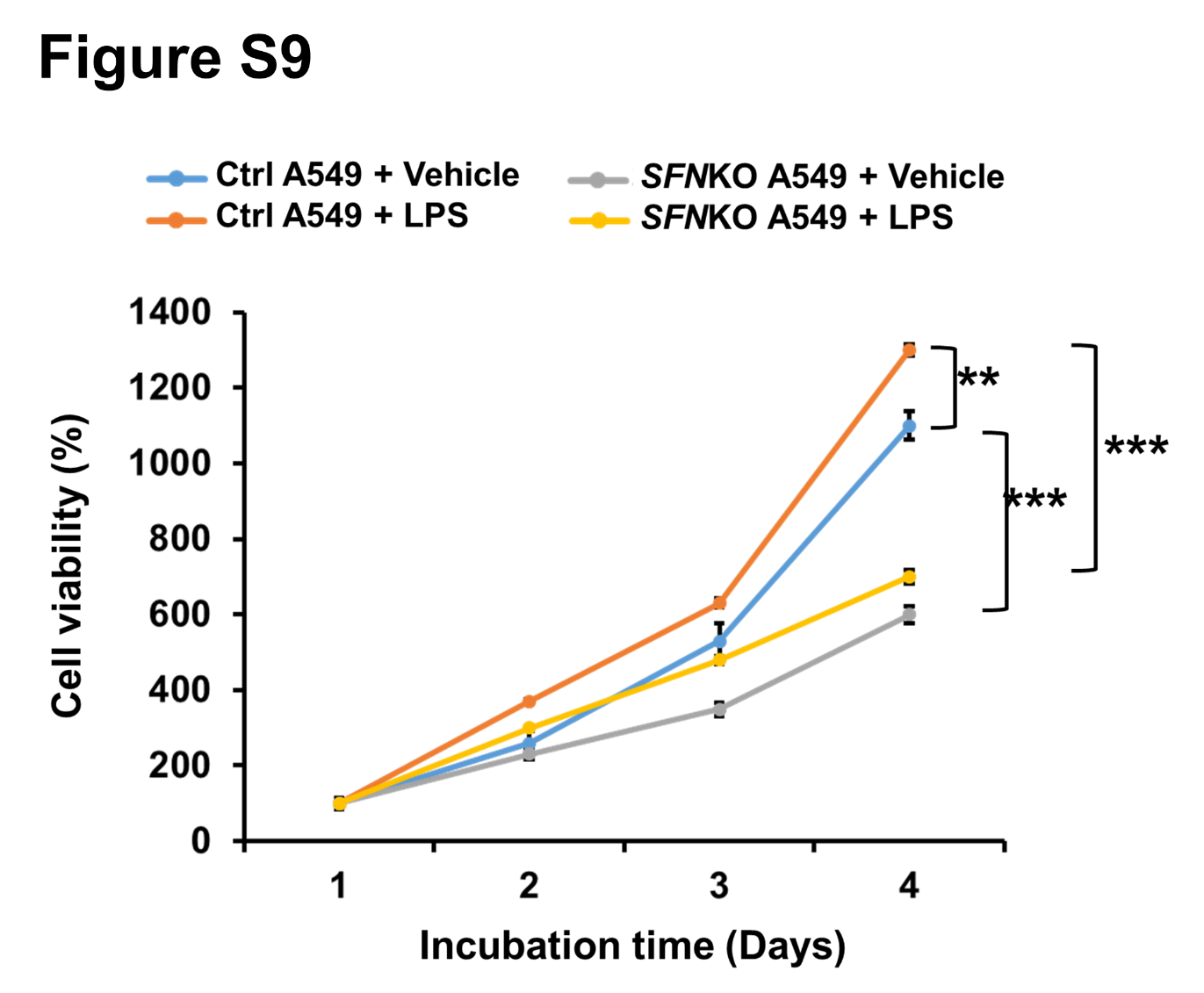


**Figure S9. Cell proliferation assay in Ctrl A549 and *SFN*KO A549 cells treated with LPS**. Ctrl A549 and *SFN*KO A549 cells were seeded in a 96-well culture plate at a density of 1000 cells/well and treated with a vehicle (DMSO, < 0.1%) and LPS (10 μg/mL), and grown in a culture medium supplemented with 10% FBS. Cells viability was measured using an MTT reagent dissolved in PBS (5 mg/ml). On the day when the measurements were taken, the medium was carefully replaced on fresh RPMI + 10% FBS with diluted MTT (1:10, 10% MTT) and incubated. After removing the incubation medium, formazan crystals were dissolved in a 200 μl solution of DMSO. MTT reduction was quantified by measuring the light absorbance at 570 nm using the ELx800 absorbance microplate reader (BioTek Instruments, VT, USA). Each test was repeated at least four times in quadruples. **p<0.01 and ***p<0.001.

**
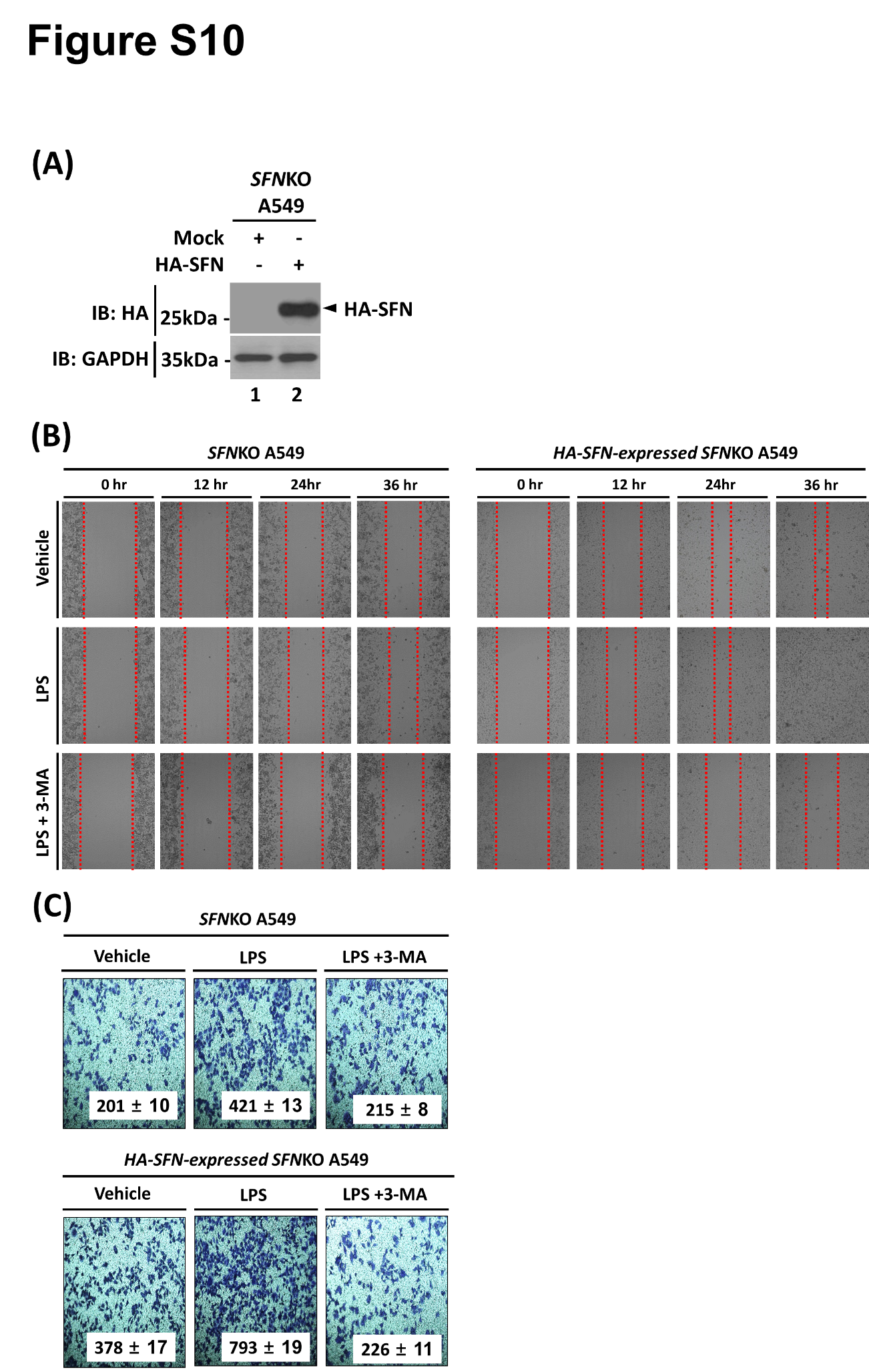
**

**Figure S10. *SFN*KO A549 cells expressed with HA-SFN increases cell migration and invasion in response to LPS.** (**A**) *SFN*KO A549 cells were transiently transfected with mock or HA-SFN vector, and HA-SFN expression was confirmed with western blotting assay with anti-HA or anti-GAPDH as a control. (**B** and **C**) Cell migration (**B**) and invasion (**C**) assay were performed with *SFN*KO A549 cells transfected with mock vector or HA-SFN-expressed *SFN*KO A549 cells, as described in Materials and Methods.
