# Supplementary material for: Stratifin (SFN) regulates lung cancer progression via nucleating the Vps34‐BECN1‐TRAF6 complex for autophagy induction
Source: Clin Transl Med. 2022 Jun 8;12(6):e896. doi: 10.1002/ctm2.896 (PMC9174881; doi:10.1002/ctm2.896)
Supplement: Supplementary file 2 — Supporting information [file CTM2-12-e896-s004.pdf]

Supplementary Table S1. Up-regulated gene lists (500 ea) based on LTT26 withthe most up-regulation of SFN. (LTT, Lung Tumor Tissue; LNT, Lung Normal Tissue)

Gene	LTT26 vs. LNT26	LTT52 vs. LNT52	LTT13 vs. LNT13	LTT17 vs. LNT17	LTT51 vs. LNT51	LTT12 vs. LNT12	LTT29 vs. LNT29
LOC649270	11.25898148	-0.417267483	-0.407945974	0.853272547	1.044757746	1.510176813	-0.392038505
RFX6	9.689885668	-0.561976798	0.12892191	1.273987133	0.885474474	-1.145486286	-0.89182298
FSD1CL	9.666784777	1.889567778	1.648564091	-3.400828958	0.318378938	-4.160181881	0.885609872
PHF19	8.707916533	1.102399077	1.743072395	1.923328076	2.147413807	3.734664011	2.388063636
LOC644507	8.476928274	-0.72214811	5.473299812	0.152886234	-0.802622536	-1.159484639	2.064132408
CRELD1	7.698249723	1.134440424	1.098361831	1.994803115	2.519067717	-0.667821642	0.187527699
MMP9	7.117217255	0.554773509	4.769965	0.511724598	3.594094847	3.871799099	4.069580647
LOC643520	7.064105466	-1.079443827	-1.182271759	-0.940384674	0.590668391	0.642376399	-0.480364796
DEPDC1B	6.97228104	1.716822519	1.69240583	2.266052558	-0.285285155	1.324218451	1.928738453
ZNF483	6.894021503	0.881530753	-3.777238933	0.055889439	-0.076017821	-2.581814406	0.017230176
DDX12	6.893577362	0.700136609	-1.383850717	2.386632825	-0.022102061	2.169553767	1.473380055
SPDEF	6.585322948	5.010246532	5.982202466	11.3230767	5.793175056	2.712432874	5.817852534
FAM153A	6.449185563	0.670243197	1.569051333	-8.725525699	-0.818741692	-1.178865811	0.570649391
BIRC5	6.332643843	1.559579462	1.166098843	1.949230484	0.385679856	2.166181556	2.193946678
CDC2	6.269925788	1.448390246	0.400817008	-2.187505314	-4.667770158	0.924917779	1.572228869
SFN	6.164919562	4.85251918	4.673445344	4.128554909	3.863265966	3.442333083	3.392045174
LOC729454	5.898178089	1.710697894	0.193619706	3.223021625	0.042097646	-1.834244647	2.831198698
LOC648639	5.895986634	0.722891804	1.358616631	0.768988498	-0.402299409	2.018107628	-0.425637475
COLEC12	5.878985907	-0.07803695	-1.180653042	0.526736408	-0.371230643	-1.6120835	-3.480016557
C15ORF37	5.826664604	1.091709346	3.554244684	-1.382654892	-0.172058723	-3.914651671	-0.586179826
LOC646223	5.764901096	-0.234354019	-0.817454984	4.401493825	-0.871631192	-1.796410226	0.720040118
LOC642083	5.726810319	-1.208309723	-0.654937951	0.251252583	5.821695842	-2.582929368	-0.293506838
LGALS7	5.726359846	-1.20272065	5.61807453	-0.538197605	1.267187535	0.29036508	0.609279493
LOC653174	5.715352047	-0.817124773	-1.059959374	0.802332751	0.649619149	0.525266992	-2.478378505
SNORD3C	5.678166421	1.789854254	4.43502116	4.732406039	2.425453795	2.532939956	6.137116605
NXF2	5.666665985	0.5858334	-0.0126516	1.345339499	-0.348511414	-2.060950427	1.516571545
HES6	5.624936107	0.342443488	0.725863209	1.388117881	1.856499892	-0.731626432	0.560540083
LOC728178	5.550781498	1.570400735	2.552706297	1.52217272	0.730746352	11.9260063	3.669158519
ZNF268	5.489542095	1.832035192	-1.630508567	0.139853895	0.098425883	-2.864661748	-6.3400356
CEACAM18	5.443442891	1.043122545	1.065352881	0.324753442	2.437593341	0.645436104	0.0193468
C6ORF27	5.399743495	-1.74531995	0.444144563	1.08449944	0.112661693	-1.840315347	1.881605427
HS.47453	5.398535444	-1.297397453	-0.248187625	-0.860988338	1.38557961	-1.162385444	0.058469903
HS.537432	5.34035728	2.020273693	0.340634651	0.036487973	-1.058001006	-0.541999381	1.424643825
TTK	5.282002689	2.272720925	1.429912875	1.397955308	0.054096649	1.361670449	2.158389384
SNORD3A	5.230300889	1.254870715	3.746173124	3.820800682	1.540005615	2.524994595	5.389801585
CA9	5.222037711	3.435790475	4.072996034	2.766525529	1.425871071	5.940020128	5.190460598
FLJ44006	5.177485657	1.286760405	-1.292423365	1.499911274	3.962355733	1.005505393	1.197108779
DDX11	5.119208634	0.014236098	2.423275041	0.467426435	1.341703676	0.32885802	0.617228856
SNORD3D	5.026604463	1.091762306	3.241769386	3.280032514	1.332214727	1.67362247	5.620424366

LOC100130508	4.975810567	-0.364563843	1.685839996	1.420623879	-0.467394354	0.075028947	0.770709845
PODXL2	4.966751986	1.902649123	1.272888156	3.1297531	2.938579048	2.48323315	1.938690468
LOC390595	4.9520931	0.887929868	0.774896448	-0.716012759	0.772695123	-1.475130597	-1.26154037
LOC100132564	4.949516088	2.054995014	2.217127474	-1.335707826	2.41584651	-0.119890352	3.592503719
LLGL2	4.847588617	0.362943645	-0.338210919	1.356064372	-1.243516543	0.977789752	3.40207749
LYPD1	4.793117987	4.37830619	5.644471205	-0.580954424	0.083737819	0.844300303	5.010018212
HS.140841	4.756202585	1.708580043	0.423231494	1.112076323	-5.20525472	0.424639222	1.095245019
PRDX2	4.73530131	-0.180503873	2.359134603	-1.124903618	-1.336863257	-0.298472184	2.052954607
FLJ35816	4.692057359	-1.835894997	1.613319858	0.488539345	-3.977490858	-0.955225954	-0.593789611
CENPF	4.683105393	0.959725229	0.861944262	1.60039896	-0.623883082	0.498914944	2.327820917
MESP1	4.61410956	4.206080235	2.435161774	0.290160566	2.622981361	3.647646804	4.819346702
HS.566857	4.606530114	-0.074452606	-0.644355721	1.082102034	-0.627503168	-1.160199042	-0.973924241
FGA	4.581592284	6.731016739	-1.04631198	-2.040016528	2.097251083	-3.512057746	-1.420289338
LOC148766	4.579481673	-1.44322781	0.12283832	-0.454486793	-0.249325618	1.177218603	5.718793484
SNX14	4.570653383	-0.194306874	1.407409111	-3.765783999	0.981946292	-0.424775171	-0.87509501
UBE2C	4.548709059	5.160920936	2.548624652	3.380533634	2.109344831	1.223136628	3.867732138
BRUNOL4	4.532122598	-0.00715512	5.085189075	0.857657662	0.735710049	-6.21129471	1.264901191
LOC440456	4.478603348	1.653410688	0.275297809	0.965674678	0.464169279	-1.174901499	1.05464109
SMO	4.470811	-1.424935821	0.275595369	2.000197364	-1.329493363	-3.358939557	2.692732221
GBA2	4.466804522	0.602190067	-2.739339047	-0.407934092	-0.328178162	-0.986429069	-0.272673667
ATPBD4	4.464606134	-0.097601099	0.839377468	0.870447906	0.265689993	-0.714457589	-0.492703877
ISL1	4.454661077	4.734645963	2.949309919	-2.494289525	4.130292216	-1.920560365	0.900227029
HELLS	4.444164935	0.277158909	-1.184463149	0.167654442	-0.274822785	-0.138043783	0.512456436
LOC100133213	4.433450649	-0.634548151	0.843704314	-0.792972973	-0.72145625	-0.945609566	-0.812197432
TMEM145	4.421603653	0.649784334	-0.259217766	0.601215882	1.464199467	-0.827205082	-0.009982407
LOC647042	4.405911939	-2.816721924	-4.761402852	2.351663408	0.115932826	2.9715043	2.661669126
PRR22	4.388232082	-3.375724865	0.403170157	1.551544419	0.400778969	-0.565107412	1.772206694
LOC145837	4.367872488	-0.515111425	-1.300391985	0.841093591	3.680229143	1.773242595	-0.731390236
LOC646663	4.351682588	0.066731795	-0.491909919	-1.379899783	-0.655052193	-0.1200144	0.711296077
ADAM8	4.349781449	3.990753976	2.571794003	2.825692227	5.942973956	2.573674428	0.445048667
UBE2C	4.345633664	3.304003271	2.49807153	2.890197655	2.480303591	1.000710382	3.10375351
HS.543983	4.344062292	1.215806113	0.248968276	1.352184956	-0.610297062	-1.621416505	-0.220169396
LOC283683	4.334708462	0.146353141	-1.483444982	-1.752012464	-1.03185558	-1.014470724	-1.45517018
RGS17	4.315526557	2.529387392	5.069999378	3.014683392	3.025343998	0.821949909	5.47508839
DNAJC12	4.307729925	5.836678615	7.021155766	2.758913807	3.823559489	-1.462475712	2.445229048
HS.555255	4.272456767	-1.375612089	-1.162959207	0.778490448	-5.802558908	-2.510317005	-0.204318591
KRTAP23-1	4.261897143	-0.246180177	-0.079875117	-1.083117565	-0.835970288	-0.886363517	0.010496925
LOC728509	4.257657194	0.249907721	-0.057596474	-1.023484729	-0.185933738	3.765590976	1.072095812
PLUNC	4.255201507	-1.245756699	5.161290348	2.817173977	0.852628984	-2.692569822	0.527581857
AAA1	4.250800448	0.003059581	-0.324141997	-0.690030892	-3.231511586	-0.197796307	0.289834622
PRR15	4.247829726	7.278522123	-0.078997213	2.625710264	0.128563749	-1.820508465	3.362737293
SLC29A4	4.242568233	0.987197482	1.123407476	1.358454709	1.287662939	1.129884762	1.968717138
HPX	4.238036099	-1.045286892	-0.538570816	1.945698699	-1.983996287	0.302735267	0.03261625

CCR2	4.224408218	-0.256782867	-0.637838836	-1.38127187	1.900736628	0.19364203	-2.713405983
RCVRN	4.21425935	-0.081029914	0.660993809	0.474595066	-2.994283131	-0.951479271	-1.725733106
LOC651017	4.186847927	0.796294716	-0.772788834	0.89371486	1.094196596	0.15861265	2.092281406
LOC646993	4.145297267	2.592598253	4.90143763	3.764874342	0.764750147	0.644888499	3.840478609
LOC654342	4.137656934	1.169943119	0.079739447	1.470354921	0.457122725	0.139213022	1.567045241
SSX6	4.130782094	0.81099562	0.632490751	-0.776320478	-1.325606985	-2.396646768	1.911914691
TFAP2A	4.108089018	4.627790586	7.052621751	5.002351089	5.282324765	0.907654043	8.996207929
ASPM	4.107121954	1.923731046	2.561124806	2.683889737	0.912821893	0.012865401	2.176957302
CACNB1	4.101598288	-0.244973925	3.500558932	0.346947822	-0.228081288	-2.574898989	-1.236462076
HS.26579	4.088026648	-0.635919288	-0.074286547	-0.852670049	-0.128355427	-4.893385252	0.871935337
LOC649346	4.042268595	0.293835323	0.440301264	-0.238290565	-1.512678147	-0.647370815	0.475223933
C20ORF103	4.038528404	9.603894514	2.240948377	5.313210207	4.432312543	0.782496266	2.329120605
LOC642393	4.029515046	-0.706188957	0.632317951	1.959655273	1.13776365	1.849578995	1.153067994
HS.436189	3.992718497	2.017716361	-0.511120615	1.716906399	-0.654812764	5.542231178	1.988474929
SMOX	3.988987551	1.156180718	2.496025498	4.133113267	2.305762549	-1.262821135	1.637897528
LOC645963	3.987925384	-1.814225931	1.546533337	1.357678459	0.757492948	-0.32559029	-2.779079328
NOS1AP	3.970330954	2.076797718	-2.491393191	-0.23544007	3.10534305	4.247739401	0.299592616
C20ORF194	3.923892759	0.245039658	-1.34464289	2.265785103	-0.060461488	-0.327134315	0.298020162
LOC100134587	3.917255603	-2.018252583	0.841638476	1.697987669	2.030748601	1.49332358	1.532768258
DTNB	3.908542855	0.311810601	2.411958589	2.753031817	2.099089292	1.447808927	2.577771953
AURKA	3.894845483	1.188626297	0.61542368	5.004398449	2.553099699	1.461907646	1.564821499
NLRP6	3.879743553	-0.050347445	-0.328326268	-1.009434099	1.468738458	-1.593976545	2.553343914
DNMT3B	3.873907795	-0.331725368	-1.614307832	0.672388242	2.694199345	0.437693776	2.104797793
HS.145444	3.869792067	0.650433803	0.878702259	1.454152124	1.152979611	0.277478689	2.285475403
GALNT6	3.84639209	1.370944119	2.42925864	1.668378454	2.662091215	0.89904898	3.690532158
HS.568058	3.844220869	0.172940383	1.055386921	3.520832626	2.294174422	1.495187021	0.86772419
POLR3D	3.842221042	0.583290185	0.445795452	0.775550316	1.65580116	-1.305378685	1.338037643
C15ORF54	3.834018276	3.138362797	2.327143115	0.946330976	3.924358857	0.183243477	0.585515659
KIF15	3.833550019	1.423876314	0.659682778	0.792432943	-2.440172366	0.430209629	2.242920262
LOC649660	3.828734452	-0.86283597	-0.216953136	3.002042137	0.212688143	-0.02076799	1.143018931
LOC653610	3.820001966	1.188843631	2.201736714	2.082119457	2.184575258	-0.053446256	1.300803049
FAM136B	3.818692621	0.326497573	0.583392892	2.939357358	3.750720843	-0.658674693	2.31024266
LOC100130673	3.804201663	-1.421697235	1.215527283	-0.670471333	-0.281666817	0.122577293	9.56664E-06
HS.544145	3.802150807	-0.908988027	-0.05557952	-2.27054122	-0.79716494	-0.516957189	0.219304274
EZH2	3.786390617	0.785256894	0.863287654	1.393499015	1.874804446	-0.132364206	2.205169718
KIFC1	3.782920543	1.079358514	2.204974057	3.5054001	2.685980399	1.344459512	1.973672146
COL7A1	3.782152081	1.257168448	4.765188226	4.092663114	-1.02324861	0.87045124	5.39539823
LOC400986	3.745171196	0.150537649	0.831180204	1.667456474	3.454810811	-0.952072976	3.117773366
UGT1A9	3.731618642	1.217841876	-1.253298858	2.417195327	0.06954513	1.851123979	-0.000672675
PROM2	3.723995096	4.698463214	4.659595786	5.512191244	3.763547643	1.613252639	4.944779298
LOC728648	3.691584	-2.184744884	-0.221396972	4.821997459	0.716019074	1.32415808	1.891695451
USP45	3.682929925	-0.516887243	0.092003673	1.984768332	1.55844092	-0.549380452	0.204885403
SCN9A	3.682880218	0.655520332	0.191236756	1.121465337	0.672934121	-1.283838126	0.550674189

LOC642397	3.681583324	0.219021869	1.10213893	0.284099572	-0.866777081	-0.282579479	3.064729208
RCOR2	3.663442744	-1.21925994	-1.172492973	0.936513918	-0.260080302	-2.148396905	1.019920566
EYA2	3.650709577	-2.047085559	2.104617622	1.97910429	-0.855591919	-1.941713517	3.751729286
LOC729389	3.646867027	-1.073885385	-0.322735799	0.584776909	2.178040642	0.292818961	0.56607741
DPF1	3.635505675	-0.867685382	-0.112246631	0.119302999	0.279308512	-2.436356456	-0.079946588
CCNF	3.624056383	1.730405749	1.361072838	1.180538011	1.441523179	0.140348786	1.187694239
HS.542544	3.616316149	1.352109856	0.671522368	-1.152927842	0.090036982	-4.54038245	2.462320814
PMS1	3.605147805	0.263920422	0.047025727	-0.120616138	0.227680501	-2.512319004	-2.175362326
DPRXP4	3.584025673	0.549201024	-1.99710053	1.054390581	-1.366608313	-0.552448049	7.20635955
FAM83A	3.580316098	3.519880512	4.971655964	6.113093892	1.506793975	3.186924007	6.410733001
ETV4	3.566510957	1.214601166	1.669677921	1.805075982	5.513216095	2.628710681	2.377141533
TRIM16	3.561363367	-2.96291715	0.689724368	0.795314146	0.69545576	0.439764643	-0.510233888
PCDHA6	3.560589535	-0.065608285	-0.688500053	-0.045791934	-1.688180935	-0.827517874	1.367195376
TOP2A	3.532901932	5.278075632	2.387677714	3.440318458	3.063068467	2.112843763	3.60998915
HNRNPCL1	3.51576658	0.035777911	-2.800783271	-0.169803079	0.352776675	-0.863633133	0.268578279
NNAT	3.50321085	0.435454801	-0.579983216	0.309763364	-0.703626702	-3.526376268	0.750702869
HS.550275	3.49559188	1.051513119	0.539807603	-1.94589609	-1.998047346	1.577466889	1.349631532
BRI3BP	3.48714341	0.677947006	-0.176348511	0.620308698	1.240169372	0.035824308	0.303355648
CDC20	3.476446495	3.241937712	1.486520389	3.262726263	1.899185734	1.900325522	3.096089223
LOC649841	3.470713192	0.81569579	0.595775601	2.380582623	1.517550644	-0.569997951	3.607140651
CDH20	3.466193784	4.126912275	-0.245511369	0.059278934	1.198428096	-1.943020819	-2.297312427
HS.545462	3.463728066	-1.197854586	-0.974524419	0.622920015	-0.254555626	-0.532339707	-0.809957354
LOC646762	3.463355227	0.42458053	1.230546917	0.540715715	-0.845860511	-1.501772372	1.05636166
OMG	3.458614343	-0.499973972	4.121394734	-1.055930697	3.506954666	3.069833281	3.759315905
HS.319406	3.439578328	-0.941518828	3.401496283	0.266738297	-1.168968479	-1.931229818	2.49489352
ECM1	3.434226582	-0.356113708	0.193419794	3.138654919	-0.973114047	-0.215994078	0.598423472
ASB7	3.428016137	1.995811701	1.040639663	0.329471343	1.983013843	-3.472972517	1.005374726
ARHGAP22	3.423172782	-0.344418943	-0.345215456	1.449291806	-2.411085031	-1.427547671	-0.4486655
CPT1B	3.410144464	0.642564073	0.526779028	1.041539675	1.49912999	1.888847925	2.375285391
OR6B1	3.409115831	0.736934816	-0.000797338	0.403101856	0.148606743	-1.151886767	0.310509523
LOC202134	3.406569366	-0.65222632	-1.135217142	-1.177524136	0.838079572	0.765069178	-0.961037463
TMEM45B	3.39970648	1.010281561	2.089146209	0.40308255	1.872158498	-0.342679357	3.25253236
LOC648438	3.385284589	0.667605252	0.028928536	-1.435722036	-2.75708247	-1.955328045	1.944913483
LOC645246	3.382633434	0.73046497	0.224442227	-1.36300678	-2.188991494	0.854750274	-0.382450267
LOC100132911	3.371570813	-4.067949867	0.085991514	-0.323562519	1.491376569	-1.948694109	-0.242328189
LOC389816	3.367802277	-0.410883724	1.210200933	3.102141881	1.335541515	1.0670814	5.528747407
TROAP	3.366499071	1.443635655	0.210036474	1.267318886	1.332517794	1.001506831	2.133442549
BCOR	3.366467031	0.375331081	-1.854452944	1.705950802	0.964460509	-0.712089079	-1.279080842
HS.572761	3.365638951	0.31014734	-1.920448253	-0.358834072	-0.76095684	-0.762609293	-0.296706488
SPC24	3.356705566	0.059249306	-0.087269093	0.900712944	0.732226596	0.803802907	1.296242488
LOC400013	3.349041038	-0.89530226	-0.237757942	-0.885969543	1.57977578	-2.630641456	0.325044439
PYY2	3.338987761	-0.0101851	0.517641935	-3.096667858	-3.78313768	-0.508742807	0.239436088
LOC729858	3.328525808	-0.385132466	-0.329080315	1.040518736	0.554225761	-0.913428847	0.246584071

THYN1	3.327729986	-0.274144852	0.077978687	0.222764941	-2.055878505	-0.95293687	2.214531589
RECQL4	3.321980866	-0.003826801	1.085827293	0.857155321	0.589177701	1.016875725	1.474118355
LOC646278	3.320924674	-0.068196955	-0.900470052	-0.458846063	1.08764887	-1.492465231	0.002988495
HS.577295	3.30564048	-0.949845505	-1.136344225	0.25956963	1.682217663	2.096645953	0.81197687
HIST1H2AJ	3.290061794	0.798795355	-3.094989121	0.813133095	-0.79326671	-0.97399661	0.022129524
CSMD2	3.288729285	1.440535217	1.888107499	3.935311104	0.228006298	-2.026990596	4.290696089
CAPN12	3.270842868	1.498558137	2.370684744	1.314747018	1.604808767	0.756851154	1.728175238
TTC9	3.266530381	1.453156599	0.844610781	2.724766211	3.268628855	-1.15039864	1.483919995
LRRC26	3.242151287	-0.098757861	1.412194276	3.31394458	1.199755669	0.046307194	5.667315355
DNMT3B	3.218026066	0.778692225	1.789647766	-0.326230679	0.599827848	0.360553948	-1.435225911
COL1A1	3.203331186	3.166850939	4.559101871	6.29697563	-2.352133294	0.098962867	5.776519541
FLJ40113	3.193745897	1.465686351	0.31562328	0.844085727	1.114747831	0.671442452	0.625399759
CYTH2	3.188728047	-0.227646165	-0.091463958	1.397674884	0.900759406	-1.106122857	1.643172737
HS.473191	3.187166247	-0.928425043	-0.624288202	2.223052495	1.329158433	0.691662287	2.14410286
FGFBP1	3.180553971	4.664429997	5.5797996	4.839287013	2.35121234	-2.039519013	0.775113315
GOLGA8A	3.180211744	0.328325043	0.190197848	1.005775623	-0.256061832	0.682314901	1.0049011
MYO19	3.177221187	0.623864757	-0.750221408	3.032064788	2.110851072	0.711181103	1.285588531
UPK1B	3.177093655	-1.604114134	0.547458413	-1.149961039	-1.595309824	-0.91767502	1.705496604
RNFT2	3.174692184	0.704427269	-0.384663434	0.40877613	-1.838415026	-2.173872149	0.205780111
MMP11	3.165695156	4.64961438	7.265721477	8.42388298	-1.336713107	2.085707617	8.516719357
LOC91431	3.162904859	-0.130847861	-0.227993885	1.772167131	1.556841662	-0.371356812	1.668733564
AUP1	3.161480321	-0.461276496	2.309956949	1.228851299	-2.532187832	-1.467476804	0.115901528
SLC30A4	3.149738178	0.375005795	-1.699958137	1.254029242	-0.025789696	0.128188107	0.167576842
LOC645330	3.147926947	-0.142799435	0.898658634	0.0848267	-0.406965936	-0.774180648	1.164341782
NXF4	3.143203423	1.208629841	-1.28017231	-1.004362631	1.622392091	1.724706231	-0.966531342
LOC401321	3.140421029	-1.369735834	0.168857945	0.659316403	-0.188490534	-0.913864905	0.262868773
MIR1180	3.13828209	1.38656372	1.155830159	-1.111134957	0.710366414	-0.145253786	0.511976274
LOC100129186	3.136362908	6.095313435	3.519320709	-1.806132798	6.647714054	2.479172831	-4.755002576
EPHB3	3.130254591	2.559453151	1.531793202	3.533943014	5.16065952	1.073413119	3.204239537
TFAP2C	3.123215054	2.072378578	1.264630616	3.504314871	3.218541527	-0.08834805	1.561754693
FLJ20674	3.119000629	-0.667310768	-0.529021194	-0.753784103	-2.735569003	-1.569123314	-3.345343901
PROSAPIP1	3.111211987	0.290732401	0.401906576	0.723099732	1.167896328	-1.037103668	0.732570391
HS.252668	3.106488023	-3.598730055	-1.071423279	0.380746229	1.105046663	-1.001668302	0.475134581
LOC100130116	3.097822598	-1.332164122	-1.092065827	1.515783675	-2.008198607	-1.965740973	-1.070316116
SERINC2	3.079592099	3.012280183	1.98446753	2.735891047	2.257841708	1.027929147	2.593333095
SLC5A8	3.079020464	-0.971014933	-0.323381782	0.417259503	0.578554418	0.001672317	0.265986309
UBE2QP2	3.078117356	1.123607871	-0.78702704	-1.150773593	1.431902975	1.111353429	0.004519601
LOC339692	3.075838111	-0.663711393	0.522979023	0.098698817	-0.683322892	-1.871006347	3.461132438
WDR86	3.063761503	0.527924249	4.032227894	4.717676043	1.179509723	-1.211349813	4.661256143
LOC650498	3.058992321	-0.956242123	2.748216587	0.902085643	0.979290053	-2.699550793	0.224116331
C20ORF94	3.056981511	0.224785909	0.387366856	2.318114962	1.316423389	0.082437951	2.38745809
CASP8	3.055750984	-0.44437641	1.234253539	-2.097404799	0.908557263	-4.83462755	-1.432679293
MRVI1	3.043331736	0.809797925	-0.684541149	1.541035987	-0.012443976	-2.061028051	-0.103359264

MIR888	3.026035596	-6.308040994	-3.223694244	-1.833477045	0.039700653	-0.861112446	1.857197307
LOC124220	3.022181288	-0.53733827	0.877086685	1.124245912	1.463331667	-0.55284598	3.198682914
GSDMB	3.016526482	3.142459556	0.660808903	5.864401675	-0.847339337	0.755915491	2.197939066
LOC645367	3.014861718	-0.049714448	-0.96563933	-2.175805794	2.26441933	2.210216068	0.984989324
HS.563922	3.013896967	-0.631036815	0.965232079	0.355048794	-0.238023292	-1.247093002	0.616463965
NFX1	3.012330315	0.754084502	-0.003545383	1.614817608	1.863209459	-0.707022993	1.37696589
LOC440345	3.011404519	-0.945856174	-0.818950496	1.000764892	1.723791858	1.308342827	0.706292251
LOC85389	3.00777317	0.709335495	1.299019401	-0.370305819	-0.694189944	-0.216032513	0.615009452
LOC732450	2.999058925	-0.471631043	-0.797417909	0.004099472	-0.417104722	-0.922071853	0.562933625
LQK1	2.996387888	0.236282724	0.02128164	-0.99819319	-1.054777476	0.394020916	0.250916493
LOC650933	2.988231927	-2.479098565	0.040820234	0.052057779	-0.02587415	-0.076344741	1.376820934
LOC650860	2.980717622	-1.55303261	-2.977631558	-0.667436923	2.181350972	2.433913861	-0.263962568
LOC100129469	2.978866871	-2.2756717	-3.480220096	-1.191427714	-0.985546578	-0.476595126	-1.815743833
CDCA5	2.975188645	1.900106553	1.559711747	2.066138613	2.420515157	0.70534172	2.035413933
PQLC2	2.970498166	0.931195262	-0.272265965	0.611314559	-0.248329063	-4.335212745	1.922429549
SLFN13	2.969204122	0.07914856	1.84293752	3.786465136	3.262329662	1.856729723	1.908175402
PAGE5	2.96528466	-2.1525202	-2.293318942	1.272269614	1.974459736	0.54227147	0.557965268
HIST1H4E	2.961690737	-0.253198281	0.128759228	0.019378659	2.226403998	-1.586839134	0.051020402
ZWINT	2.958281262	3.242940314	0.89533651	-1.92516511	1.402223284	1.147613746	2.522077342
LOC727759	2.943556071	-0.009916031	-0.726620907	0.260893902	-0.628862978	-1.043727737	3.121616211
STX1A	2.939985832	1.587011266	4.024905322	2.177144122	2.795010548	0.859448861	4.665663623
AFG3L1	2.939702773	-2.727332167	0.483084707	-0.232477526	-1.428659282	0.442589825	-0.604337325
LOC646864	2.938939245	-0.02790478	0.423961572	1.426368289	-0.800570214	0.37302699	0.059103604
VSIG1	2.938795658	10.80522344	4.475306424	1.40385069	4.740354923	-2.343361589	0.56479989
PRC1	2.935466289	2.942754325	1.415612726	2.396459098	1.483971211	0.027698078	1.585619277
C1QTNF6	2.933570846	1.04336769	3.223327114	4.751604629	0.687539762	-0.696792441	3.1975692
PDE9A	2.917163093	-2.523145221	-0.369125235	-0.445710686	2.314626492	-0.626614604	0.868148266
FLJ13305	2.907900807	-4.328022609	-0.516072174	1.375137207	-2.210966126	-0.220238438	1.846643133
LOC646774	2.905223979	0.991519156	0.793744523	0.519566998	0.254081454	-0.697837926	-1.233180704
LOC440335	2.901882631	2.321547084	2.92320173	2.290006448	2.834371735	1.602617912	2.167543665
MCF2L	2.888361798	-1.164820313	-0.166745042	-0.971892733	-0.491984305	-2.454109519	0.218511106
HS.561844	2.885087604	0.536113636	0.864007818	-0.4933486	1.568640767	-1.681748933	0.268600975
LOC649722	2.884818125	-1.462538212	-0.097471411	2.069606682	-1.909006636	-1.207395443	2.159841821
GRM4	2.882662611	-0.377293504	0.190240667	0.394208282	2.099373121	-1.593475999	0.613238415
ORC6L	2.876530492	1.358606804	1.907158546	-0.129470725	0.632820378	0.109133822	3.173089793
RPRM	2.875907696	-0.542795898	2.620835964	0.986759825	0.989368166	-2.185738158	-4.521136354
KIAA0101	2.87521159	1.384283207	-0.735494636	0.930598122	1.363288082	1.689928554	0.922013547
TCEB1P3	2.87410519	0.340419445	-0.684580086	-0.063681458	1.919775523	-0.117001789	0.227411729
TACC3	2.865532441	0.111890765	-0.182944772	1.088763698	2.401663979	0.612077776	1.762816736
C6ORF141	2.859007805	3.559386676	3.729727316	1.928414407	4.012445078	2.888559823	1.838866636
LOC100132656	2.857819226	-0.682216168	0.499925783	0.44277828	-2.713032424	-2.939700855	-0.803589264
HS.561915	2.853181071	0.58513279	0.674425542	0.602018506	0.333156895	-0.880331304	1.289294054
ING5	2.849099738	-2.456044795	-1.336391314	0.090786852	-0.189764599	-1.981854683	-1.807760564

HS.435263	2.847881491	0.823024405	2.339335668	1.387022157	3.554094109	0.286036975	2.800045273
SNORD83B	2.846356179	0.657729269	0.950271518	1.085278161	0.467549293	-0.076241059	1.026261485
LOC728034	2.843170527	0.249533275	-0.402936567	2.838831102	1.582540805	-2.230678419	1.304969116
PNPLA3	2.84192018	1.379166093	-0.206122735	1.930065833	-2.274615021	-1.314736476	2.172864366
LOC644629	2.824534521	0.229852532	-2.370430155	-0.388397169	-0.943425448	-2.456156608	0.28156378
DDX27	2.823221961	-0.682188604	-0.226989629	0.775004017	0.473367645	-0.082830433	0.607962309
HS.560542	2.815031873	-2.278608684	-0.511066468	0.103481287	-0.607916082	-1.446738634	-1.836246466
CRYGS	2.809925937	-3.18863204	0.339067264	1.934168734	2.462334004	-0.219592075	3.729516329
GAS2L3	2.808141513	0.340704336	-0.303346928	0.882962574	1.017719589	-1.608721586	0.608314184
UCP3	2.807776245	-1.395627099	0.559614955	3.491440858	-0.566782492	-1.542691518	2.298872109
CAPN12	2.806130158	1.702869179	1.136485069	0.837789218	0.400640875	-0.177514531	1.393470019
ADM2	2.802116979	4.505824475	3.981268735	3.01910678	4.288334039	2.219354194	2.712233988
RNU4-1	2.796328463	-0.545714287	0.698261175	-2.488836146	1.030852302	-1.799679862	1.135588694
LOC100132288	2.787032182	0.879396515	-1.113110922	-1.12642461	-0.405190958	-2.990670692	-0.178516585
ETV4	2.784086857	1.625012114	1.097259629	2.250625035	4.802364517	2.230170481	2.706599203
HS.570192	2.781708224	1.100007631	3.774803354	-0.602288833	-2.527703637	-1.993592265	-1.329832608
TTLL8	2.778518724	-1.033648553	3.910525174	-0.586272482	1.041327184	-0.584838957	-1.1383851
FBXO5	2.77719538	0.190765093	0.146327318	0.531982139	0.058841842	-1.945266398	0.735933918
LOC652736	2.776687521	-0.356809418	-0.017652724	-0.81031922	0.434028335	-1.219449444	-1.090560499
C17ORF53	2.769941526	0.785353926	0.869054505	1.40288046	0.582343557	1.368268767	1.222345065
DMRTA2	2.767181434	-0.081095538	1.313534786	-0.616857566	-0.222157046	2.868530892	0.061690042
GAFA1	2.762137081	1.212238511	-3.689671886	-0.828588736	0.425968782	1.491481713	-3.843370201
LOC731432	2.760868489	0.042215914	-0.809245596	-0.49812165	0.867621401	-0.37927168	-1.090945589
HS.582113	2.76018181	-0.467360996	-0.137260816	0.416588151	0.816789455	1.236872716	0.137298357
MESP2	2.75713907	1.407043036	-0.730227833	-4.112046556	1.544074571	0.283659944	-0.635254268
KCNA6	2.749333873	3.967418599	3.307096904	1.253193927	2.09799769	-0.508639863	-0.498014792
ERGIC1	2.745640301	-0.928007177	-0.420555009	1.115061563	1.052478125	0.500653993	0.489862489
C3ORF57	2.74186222	4.267308606	4.380153591	-4.256859943	1.622951593	-1.210057543	0.22710407
HS.558911	2.740022273	1.327266865	1.239821554	2.814302904	0.504860035	2.34602851	-0.609341178
LOC730173	2.739943	-0.196930698	0.003641261	1.31192595	0.511802615	-1.233255642	1.169808249
UPF3A	2.735224057	-0.738373571	-0.710197093	0.323162791	0.183190559	-1.391500276	0.474949224
PRSS22	2.730346497	0.072489763	3.655248351	4.143381872	4.546972822	-0.490453155	1.994713105
PYCR1	2.727879645	2.503292951	0.934768489	3.516918609	2.358864758	1.884133863	3.486658307
RGL3	2.721640661	-0.422948183	-2.915654036	-0.021098455	0.48924436	-0.48405829	1.363325429
LOC100133760	2.720866489	1.039922236	0.45087147	2.167361161	0.37023617	-2.971642076	1.762553981
TBCD	2.720743821	-0.138215803	-0.88261322	0.491871856	1.654081597	1.208919679	-1.156118031
SDK2	2.720686417	-0.104630206	-0.516149141	-0.586363067	-1.522476447	0.597816303	-0.283327102
SLC13A3	2.718538362	1.178434124	0.122428652	-0.019280226	1.43332875	-1.507264097	-0.390331057
CCNA2	2.718397113	0.72423259	0.444430886	1.266985941	2.78233722	-0.822543511	1.833852034
OR4C45	2.716540986	1.204413091	-1.948113253	-0.565535061	-0.062177523	-2.654539767	-0.159731644
SLC6A8	2.714802211	-2.541012533	1.085023372	6.311582206	0.640925692	-1.193564028	3.65464033
CHTF18	2.714024509	0.45605851	0.035973212	1.764142324	0.454536686	0.951143833	0.400379279
HS.577681	2.713270637	0.762757294	-2.591746282	0.836646853	0.960638537	1.141282486	0.924808017

HS.537695	2.712302125	0.714934068	-0.561657431	1.48542131	-3.95485014	-1.672550833	5.931732026
LIN9	2.706775279	0.617348631	-1.030500645	-0.520741184	-1.548687691	-0.73974178	-0.482634669
LOC100128007	2.705362505	-0.430997665	0.355442338	-0.940647389	2.080909562	0.978946013	0.657232724
PAQR4	2.705210354	0.625241544	1.527961261	1.123297462	0.997346985	-0.451372977	1.947373841
HS.562701	2.704196898	-2.98238607	0.851070678	-0.330424011	-0.055588679	-1.158519225	-0.306338056
PRAMEF15	2.703654112	-0.257242903	-0.609820993	0.094064004	0.999762857	-2.23086194	-1.15813664
FLJ44790	2.698288873	-0.715982173	-0.519869637	1.15716051	0.77822458	-1.787366195	0.254587451
HS.562641	2.697007374	4.215736369	1.505290094	-0.131292918	-2.806594869	-1.070428962	-0.21642702
SLC39A3	2.694463414	-0.486465449	0.737357499	1.520166696	1.636629096	-1.150849702	-0.494385446
LOC441066	2.694070109	0.360023947	-0.869737121	0.655956006	3.23208623	1.590034456	3.008286381
DGKZ	2.692744063	0.148540238	1.128582205	-0.658249019	-0.743358022	-1.644588456	-0.374830317
GPR180	2.691316841	0.493630533	0.908703744	1.691430703	-0.257187171	-1.508848828	0.784620795
FAM178B	2.678847755	4.154988369	2.299896383	0.244248508	0.541925626	0.325274363	1.47296548
TFF3	2.67818128	2.625580149	2.317888201	-2.316943443	2.147933044	-0.169605821	2.230529124
CACNA1I	2.676944882	0.710204898	2.215858049	-1.923670132	0.191037316	-1.87360815	-0.752000615
C16ORF79	2.674342151	-1.593842478	-0.86487151	1.321084435	0.028058774	-0.1573719	0.360210422
C17ORF81	2.671154475	1.883188993	1.292576249	0.228011281	-1.253333897	2.693578004	0.736258819
SLC35C2	2.66931274	-0.140824018	-0.065816891	2.323970843	1.517310561	0.188670928	0.327019038
MGC102966	2.668354191	3.379146456	8.128965079	4.183098843	6.522065046	-2.212091408	0.827063528
FLJ22184	2.664866829	1.668087863	1.43518881	0.68340881	0.911315449	-0.526115485	2.363464154
RN7SK	2.664798261	1.212770485	4.183246927	-9.762998693	-2.87063695	-6.73589012	1.382844367
ADORA1	2.655434318	2.41466902	1.448549771	1.286700511	3.558002693	0.407241377	2.560765177
LOC440030	2.65029461	-1.661119652	0.51321923	0.524673579	0.949184552	-0.371883329	1.195231041
NUF2	2.649046026	3.290761273	4.461532569	1.658315971	-0.677580335	1.444181328	0.45321296
GSDMB	2.6459299	0.68615833	0.467260006	1.305504967	2.29187487	4.026917097	1.291584861
TCL1B	2.64312187	-0.99795809	-1.132198143	0.46766056	0.832293517	1.362742516	1.248375611
MB	2.63819061	1.599191831	-0.723746103	4.873817793	1.526373538	-0.934177017	2.619053735
PAFAH1B3	2.63291619	1.421084746	1.645157137	2.10991228	1.216829765	1.748951111	2.298445059
SNHG7	2.628526012	-1.229597586	-0.758469435	0.721112908	1.083216986	-0.516246839	0.576772802
PPP1R14D	2.627181325	2.446361768	0.946928187	0.750315242	3.15101376	3.054273634	1.231998408
PLEKHN1	2.627165293	1.632897926	0.573500198	2.936858057	1.849352164	0.576997688	1.597910623
POMT2	2.620531985	-0.747341471	-0.111315157	1.471731856	0.444444114	-0.241535064	1.889063236
CEL	2.620117036	-0.86730369	-0.010892589	0.871708037	-0.777046262	-2.697772403	-0.424500432
NUSAP1	2.619274828	2.87256555	1.858529803	1.716175731	2.296581709	0.846712133	2.614595581
LOC388621	2.616692254	0.716454741	3.810378345	-0.605925105	-3.172311013	-0.51206939	0.310183005
HS.568294	2.616322415	1.899688127	0.551647836	-1.817970167	-0.238584407	0.1284187	-0.165399599
FLJ22536	2.615586967	-1.232554288	-0.380773659	1.763754908	-6.473173811	-0.981568626	1.460353481
SSTR2	2.605199235	-1.33395319	-0.455858521	0.773992561	1.125378343	0.027789916	0.102935208
TAF13	2.603952686	-0.249145971	0.171494432	-0.23038812	0.706339576	-3.055676927	0.770933534
FLJ40125	2.600199939	-0.456298497	1.438053683	0.079802115	1.757752983	1.311490353	1.524856109
LOC653524	2.5998761	-0.182966181	0.250106413	1.14166525	-0.817063699	-0.198489391	1.937371411
LOC338799	2.595305644	0.235018594	-1.09900319	-2.823984857	-2.490872375	-1.947742647	0.709281391
SNORD80	2.58999408	1.686049207	1.596097922	0.908889192	0.964921161	-0.778572041	4.264921767

VAC14	2.587909845	-2.026843858	-1.081389047	6.835911637	-6.422947227	-0.768473028	-0.373948612
GDPD5	2.582065777	-3.382459347	-1.76643977	1.425500863	0.786716326	-1.861926666	0.262148813
XRCC3	2.580070973	0.385770627	0.615855901	1.238412035	1.033365144	-0.099690376	0.850408335
LOC728408	2.579420667	-0.607323843	0.1584661	-0.28712642	-0.535401717	1.976403202	1.294571637
LOC642433	2.576352617	1.164718666	-0.146350977	-0.466228709	0.103239291	-0.590556781	-0.268439352
FKBP9L	2.562102606	-1.340969423	-0.720813336	3.800385488	0.51155204	-4.280914313	-1.762242053
SNORD79	2.561848651	-0.26529972	0.262750189	0.178620872	-0.585857654	-0.088040588	-3.425298472
C6ORF223	2.559338294	-0.239716454	1.594316276	1.972964263	-0.103019117	2.266037986	2.090416473
DISP2	2.558254283	-0.087610897	0.343870432	0.45930574	0.872996085	-0.844181569	0.171533647
NPW	2.557923068	-0.387544494	0.859834832	1.756230711	2.421265704	-1.173222946	0.754867342
CRLF1	2.552493884	-0.067583314	2.7596017	1.889738202	1.725964628	-1.121796136	5.111907792
CAMK2B	2.551635044	-2.87100554	-0.418833731	0.486165709	-0.508400943	0.101453728	0.249663753
MRPL10	2.548663013	0.093614586	0.033397941	-1.631197248	-0.198990216	-0.621739383	0.76350586
NSUN5	2.548509136	-1.626531389	-2.93362798	3.160768648	1.541436289	2.180180054	4.864408617
CDC2L2	2.546250765	-0.74652794	-0.496962638	1.06473208	0.970987099	1.350879156	1.219194866
STAG3L3	2.543783531	-1.751666339	-0.437467542	1.142154254	0.928451067	1.211311168	1.461409627
PC	2.540964041	2.859338217	4.777525716	1.423944243	5.919602191	5.501359193	1.042944893
ZNF692	2.538188012	0.080499854	-0.884383472	1.117413268	1.211763907	0.695405304	1.558746889
GGT1	2.535480302	-0.161868204	-2.859270913	-3.072579296	0.34593209	2.448101694	-0.151244447
MGC23284	2.532668708	0.669372074	-1.02232771	0.58299455	1.324769114	-0.696900825	0.301595776
PLEKHG2	2.527982192	-1.635653066	-0.409752072	1.441497291	0.070483959	-1.977389037	0.476428
C13ORF16	2.526069106	1.428998516	0.64733603	1.682293108	1.068364163	-1.788687544	-0.74278114
LOC727773	2.522085636	0.203506107	-0.288316803	-2.177128995	-0.838018355	-2.569612134	-0.457036678
LOC100133673	2.521587459	-0.030418574	-0.027021109	2.193231863	4.600545264	0.923508496	2.099033283
SNORA57	2.519847879	1.224108479	1.216581721	1.678709623	1.746352074	-0.918883679	1.800858329
HS.90866	2.519667536	1.196922014	1.456283278	0.500241806	-1.505600412	-2.815137123	0.150202225
ACR	2.518631357	0.748153467	-0.385301178	2.523332744	2.202955429	-0.472354	10.4207334
TMEM169	2.516783525	0.676427779	0.745254713	0.707889046	-1.330993693	-1.612827005	2.822083415
RECQL5	2.513884906	-0.522982494	-0.126706237	3.554748215	1.227051516	1.227344699	1.852220937
LOC649639	2.513527375	-0.6246889	0.325754175	2.203457835	2.158322005	-0.54060161	0.533780112
GSTZ1	2.511989511	1.084613403	-0.520954676	0.732336118	-0.404347795	-1.173773195	0.571520594
CCDC137	2.509735337	-0.137690821	0.182101825	0.058393793	0.412691236	-0.784130515	1.51505886
MIR939	2.506863542	-0.095104983	-0.009680713	-0.123687229	0.475725929	2.402074655	1.18890257
SNORA80	2.503753943	0.915148409	-0.062553419	0.777836633	2.140793759	0.251491595	0.172699327
HS.568690	2.502308291	3.876326926	4.150882931	2.723763576	1.411190013	-1.433639271	2.530334444
CDKN2A	2.494461659	3.566032174	1.702141648	2.285788226	0.286472942	1.750463772	0.118172886
LOC650463	2.491710928	-1.113445114	0.421438162	4.010311246	-2.469772969	0.085560849	2.5812285
HS.444999	2.489553022	-0.251804536	0.594575489	5.230305844	2.211815418	-1.039392008	1.738662407
LOC646547	2.489000195	-1.133160413	-0.288423635	1.427377982	1.329807378	-0.253943537	0.145724113
LOC653270	2.485248859	1.209181387	0.747831162	0.495905812	-0.554097248	-1.227646613	-0.105512866
LOC652726	2.485156148	-0.582142669	0.393316216	1.718274695	1.578749739	-2.43064512	2.668991121
MELK	2.483768726	5.266143248	2.200463478	1.425773179	2.352545292	0.909090205	2.534795851
ASF1B	2.483382985	2.248433213	1.610446158	0.873984434	1.455707424	1.438644311	2.950812247

LOC648226	2.47962684	2.755317419	-0.970445768	0.337975857	0.197852166	-1.750694547	1.414430751
LOC100130764	2.47727988	0.601798901	-0.281237164	-0.970152653	-2.122743234	-0.261774849	-0.20204055
HOXB6	2.477004473	0.405801724	-0.571855632	0.125102952	1.146928995	-1.86647146	1.382535452
C2ORF3	2.473769423	1.721408156	-0.467280428	-0.37802835	1.537199164	-0.918410353	-0.456073604
LOC728485	2.473096965	-1.061000454	-1.028137513	-0.028043352	3.112528276	1.02898659	1.679787665
PPM1B	2.47207781	-2.576444663	-1.009468964	0.283520065	1.582882312	0.516355741	0.777917583
NEK8	2.467648464	-1.000170376	-0.455063373	0.033423888	1.169430942	1.416352036	0.444725874
RNU1F1	2.466247754	-0.590742701	-1.179756346	-2.977740184	-0.100011371	-3.467517196	-0.069303031
EME1	2.464373645	-1.948510302	0.627528576	-0.383684367	0.236363863	0.332515607	1.468170143
NCDN	2.463040148	0.834515353	-0.966781549	2.125362091	2.147861867	0.315630164	1.368568571
ANXA7	2.461175623	-0.621815565	0.817642179	-1.021700396	-1.588301246	-1.844230013	1.200082227
RBM10	2.45579817	-0.211278238	-1.369027653	-0.961744342	-1.573782427	0.037252384	0.326738074
ATP2A3	2.454654968	-2.032037457	-0.289910864	-1.427558727	0.337567836	-2.064329405	0.146544184
ATF7IP2	2.450238691	0.697877177	0.530730654	1.652281668	2.901604136	-1.130834962	0.23728433
LOC646817	2.449395656	0.397919221	0.145100926	1.702119303	-0.222021253	-1.923009441	0.298886264
HLA-DQB2	2.448523469	4.613497362	5.258973465	2.029251352	3.458825466	1.268938588	0.507435052
C20ORF91	2.446745701	0.501501301	2.581832911	1.869847238	-0.134452859	1.157680572	-6.335343695
LOC728715	2.445830268	-2.69185133	-1.785261344	-0.282831794	-1.773819275	-2.333976182	-1.889221685
FOXM1	2.445505348	1.299728039	1.102711393	5.289498576	0.943858655	1.65380419	2.932369532
NOM1	2.442466638	-1.463924266	-1.520797867	0.800441146	1.777932155	0.097500033	0.692829815
TBC1D3I	2.438128641	-0.870930097	0.612400419	1.371266266	0.266599627	0.905625207	0.708210711
LOC100131669	2.438017777	1.726429698	-0.744827677	1.002731752	0.941884902	-1.858606143	-2.032050499
HS.541685	2.43650754	-1.166079073	-0.455744632	0.884006563	0.556285641	-0.26934804	0.329715634
LOC100129094	2.434742986	-1.544370871	-0.478175218	0.867858623	0.690755518	0.298461038	0.532480358
DKFZP564N2472	2.432813537	-0.823666983	0.011417962	0.123104673	0.447257794	-0.814848091	-0.221981317
SPG21	2.432664507	6.19307524	0.587985461	-1.055276452	-1.065582689	-0.509914398	2.32649069
HS.568157	2.425674129	0.488041804	0.665780492	-0.244789451	-1.826597129	-3.73411607	-0.101665729
PCDHA9	2.421581028	2.237911235	1.815257956	0.195052943	-0.573915673	0.013986066	-0.323351498
SNORD59B	2.418459864	1.701629874	1.48692998	0.550341185	0.660788171	0.803003377	3.7215678
HS.127078	2.417846198	2.484977734	-3.143943187	-1.2481996	0.070115813	-0.516202469	-1.081711918
LOC387941	2.417512569	-0.079296304	1.62151225	1.485934955	-0.416160865	-0.20515104	0.314028612
SAPS2	2.416959983	0.867965319	-0.189858486	0.635847353	1.832385239	-0.118167493	-0.054499388
ALG1L	2.415454755	1.63198296	3.660895636	4.910252902	3.150248247	3.009022873	2.937616542
PABPC1L	2.41145571	1.709810961	1.820841238	3.351940511	0.979502084	1.830371748	1.349047418
AADAT	2.410399575	-0.192524495	-0.881518803	0.651546255	-0.140040777	0.471836202	0.763549557
PRIMA1	2.409290691	0.334246931	1.575176641	-0.133531953	-1.178122193	-0.465292601	0.583069711
LOC132203	2.40900936	0.316783842	1.097219672	-2.426745546	1.923523298	-0.849445444	-0.617127973
LOC202134	2.405459819	2.486887698	1.530456514	-0.745696769	-2.580546523	-1.520721747	-4.685146872
LOC642960	2.405213206	0.892126966	-0.017794498	0.168495067	-1.18692351	-2.162844492	1.28002025
PAK1IP1	2.402750949	0.34152818	0.906579605	1.310625385	1.294618988	-2.191426086	0.898836317
STAG3L2	2.402671754	-1.171372927	-0.740134051	0.714086254	0.6503052	1.093435348	0.961350219
SLC25A20	2.402329953	-0.980527911	-0.673076188	0.474657039	0.3956267	0.333183143	0.344654293
HS.554507	2.402232629	-0.585417347	0.514776347	0.254823191	1.30465586	-0.011327793	1.191627796

MGC33948	2.397014716	0.987763307	-0.119279655	2.161305278	-0.799699209	0.637082962	-1.787029782
DSCR6	2.396532154	-2.738928988	-1.558251699	2.232574662	1.356511513	-0.400194631	-5.302980723
LOC653569	2.395402262	-2.608721319	0.425048876	0.67414381	-0.934827934	6.113356575	-7.051030289
LOC647630	2.391486554	-0.364970766	1.09948586	-0.038510261	0.909885382	-0.307709632	-2.990308298
LOC728093	2.389716532	0.070833065	0.474941076	1.581108876	-1.601654952	-2.04809168	1.307356217
LOC653800	2.38872452	-1.937697206	-1.719909707	-0.766833913	0.196362834	-0.445080242	-0.199720841
LOC440518	2.383712967	0.251513068	-0.432616936	1.390501473	0.960206072	-0.797042435	-0.455765741
C1ORF117	2.38007701	2.152422517	2.152130788	1.333739958	0.682678669	-3.532115493	0.331601889
LOC642787	2.378699255	-1.659164462	-1.056841351	1.630015451	1.671916549	0.428321172	5.340620404
PIGX	2.378637215	-1.175083179	-0.591678332	0.790005002	0.843014868	-0.019162255	0.184917004
POL3S	2.375762451	-0.204276933	0.225890452	2.557792358	-0.102746706	-1.518767525	1.050657487
RASL10A	2.374999603	-3.099169005	0.876509813	0.483375877	0.899711098	-3.469920284	-1.1642368
CHRDL2	2.371655974	0.227879635	0.132665877	1.316138843	1.020325887	-1.77266411	0.635322544
SYTL2	2.371385459	0.333250414	0.991998203	1.259572269	0.607186854	-2.602559653	1.066869288
IMPG2	2.366362095	-0.522404307	-0.535442669	3.318665626	-0.215123406	-0.746050649	-3.257062924
LOC728953	2.360859667	-0.744343726	-0.386695809	0.634218458	0.523043372	0.816070221	0.456829564
IL18BP	2.360404253	-0.573067713	-0.285617743	-0.209466854	0.748777238	-1.296345727	0.33383711
LOC100128591	2.358201119	-2.347840933	-1.462254561	0.830507607	0.99233898	0.708259856	1.335847506
PLXNB3	2.356808102	1.60863374	1.731376866	0.926463807	0.706002988	0.610126972	2.763078245
SIX4	2.356090835	1.904677919	5.700046756	5.091261123	3.122647293	1.617357294	4.395083919
RMRP	2.355332016	-0.096540484	-1.321836014	-3.876368691	-1.742895949	-6.567987583	2.016203877
METTL2B	2.354807151	-2.677564181	0.079562772	0.184987536	0.550908673	0.16954534	0.548166544
LOC649712	2.350485935	0.512005508	0.736416435	0.007349567	-1.126502509	-0.083562111	-0.618339548
SDK1	2.350014514	-0.497193097	0.79424962	-1.506417789	2.53665234	0.108587524	1.648392142
LOC100132428	2.347359006	-0.526320783	-0.462196927	1.62523103	1.016487279	0.104549966	0.478078062
LOC158301	2.346670857	-1.395752282	-0.452303074	1.031877605	1.07517659	0.150323208	0.82102658
PPP2R4	2.345114702	-1.615620713	-0.130745388	3.4396462	2.207742485	-5.834795995	2.111326077
RFX4	2.342180047	-1.28028747	-0.89808313	0.485185753	0.758751443	-0.340330947	0.138544556
METTL11A	2.339873309	-0.050097104	1.721605022	2.355478237	1.097339412	-2.174868659	1.968578451
TPM3	2.33971403	0.028175518	-0.338420539	-0.004425578	-0.360625814	-1.745181221	-0.170880562
ANKRD44	2.339151485	-1.507836661	-1.086399527	0.869134373	0.490453094	-0.160793288	0.12589132
LOC647568	2.332785227	1.529471754	0.803642236	-0.71489124	0.599496116	-0.332632813	2.440190711
LOC100130952	2.332348042	-0.878255767	0.080527733	0.779786722	-0.130917436	0.199868299	-0.053975203
LOC731789	2.328625847	-1.112819086	-0.263280409	0.817107702	0.858628494	0.373508629	0.525844325
ERI2	2.32699798	2.217407871	1.575227055	0.058209464	0.404754035	-2.688863395	0.623736817
DNHL1	2.326546252	-3.041872671	-1.614909306	0.737365156	1.645924454	-0.378886676	0.74363779
LOC100128510	2.325241042	-0.716685951	-0.563950675	0.855176616	0.772302055	0.091606887	0.697650553
AGPAT2	2.321839859	1.261928117	0.157527943	0.541216907	0.6601927	-2.128394512	0.396453475
RAP1GDS1	2.320730991	0.276070142	-0.435723286	-1.161769073	0.833312619	-3.689007438	-1.586471858
LOC730240	2.317037928	-2.205051119	0.241842777	0.272623962	0.304630281	-1.229227457	0.771903722
LOC727899	2.314226602	-1.217919934	-0.942622144	-1.33948289	2.561293949	-0.637422535	0.130100932
KIAA0773	2.312418087	-0.761492898	-0.226432074	-1.278613081	-2.051235602	-0.333225325	2.626522192
HS.98588	2.311208324	0.436922249	0.599637369	0.891551698	-0.935224035	-1.444259508	-0.75528034

MBTD1	2.308019747	-0.875482489	-0.730256337	1.008708555	0.806393054	0.725262289	1.206142245
CUEDC1	2.306889723	0.666582854	0.273646456	0.368585077	-0.250166507	-0.771117176	0.533303732
FCAR	2.305258911	-1.338539462	-0.826533075	0.628922099	0.829212802	-0.398379346	-0.134528021
CTHRC1	2.30281367	3.323530099	4.357768129	4.864850207	-1.807074276	-2.297252055	4.732781651
RNU4-2	2.302313116	-0.684135868	0.429931045	-2.007668048	0.855614786	-1.88881105	0.889793359
FAM21C	2.301214149	4.708874843	-0.55247026	-1.324084997	-4.150559628	0.460431029	-0.07706631
LOC100133019	2.299491338	2.177565717	2.393178209	1.111522236	1.939485885	0.151525515	0.510812626
ANKRD52	2.29028681	-1.442801787	-1.414538562	1.143902316	-0.2188244	-2.446357855	2.394947022
POU2F1	2.289283483	-0.479617	-0.645905719	0.764863721	0.688372628	0.55907449	0.544670213
TCF7	2.286037222	0.410574502	0.613328533	-0.248948985	-0.741977412	-1.304867942	0.781739955
PTP4A2	2.286005201	-0.88922712	-0.248571788	1.302198418	1.058966026	0.971650702	0.458593742
C12ORF11	2.284926575	0.631620881	-0.144563735	-0.628281772	-0.070633644	-1.445176837	1.277168029
ZFP14	2.28250898	-1.027051648	-1.259580807	-2.012942803	0.140283871	-2.028984674	0.431486728
FGL1	2.281315597	3.310800644	2.550610576	-0.193166054	4.358039324	0.267930075	5.685234247
CSE1L	2.278724275	-0.691480014	-0.685346875	1.049693759	1.171456882	-0.527688839	0.63838392
LOC388122	2.276813076	0.387195921	0.266766947	0.595829813	0.439409988	-2.478155993	-1.037595669
HOOK1	2.276406063	0.772444444	0.852357511	1.782403555	1.899325554	-0.112097308	1.910851521
TTC14	2.274710472	-0.645212268	-0.21371004	1.772469585	0.378564846	-1.424577813	-0.135704916
TMEM132A	2.27443927	-0.216194127	2.125826715	3.551721592	1.143392091	-1.733237843	2.777674832
LOC100133120	2.267078908	-0.242109543	-0.018727046	-0.563539957	-7.479564132	6.048541299	0.634234794
CACYBP	2.266304501	-2.105949818	-1.737543792	0.607876148	1.402051173	0.68567048	-0.284798742
SLC30A4	2.264185264	-0.762909629	-3.175121363	3.275435844	1.498166058	0.059582533	0.212431968
MCM2	2.262690593	1.813313436	0.701004229	1.084150698	3.09301386	0.833150319	1.900500344
TRAF2	2.261306335	-0.801633383	-0.624627186	1.288640234	1.00103945	0.171232392	1.057810897
MBTD1	2.258763565	-1.36299558	-0.483189713	0.379487586	0.796174585	-0.549259346	0.309823605
LOC729780	2.25718082	-0.489867529	-0.108460327	0.88570567	1.503805534	-0.052126141	-0.093847421
LCN12	2.254984152	0.048436002	1.834335066	1.868151472	-0.397100212	-1.18685093	1.097576724
HIST2H2AA3	2.254967887	0.954293964	1.392877182	1.049580687	2.235167847	-0.590860219	0.576365826
ZNF493	2.254295182	-0.853567088	0.122618863	1.583272018	1.517330133	-0.04989102	1.31158035
TAF13	2.252739165	-0.255071591	0.31245224	0.275163695	1.455530031	-3.261794858	1.168571537
SNORA65	2.252669756	-1.239941969	-0.327733506	0.014613801	0.37937296	-1.596620723	1.016330869
