# Supplementary material for: Stratifin (SFN) regulates lung cancer progression via nucleating the Vps34‐BECN1‐TRAF6 complex for autophagy induction
Source: Clin Transl Med. 2022 Jun 8;12(6):e896. doi: 10.1002/ctm2.896 (PMC9174881; doi:10.1002/ctm2.896)
Supplement: Supplementary file 3 — Supporting information [file CTM2-12-e896-s002.pdf]

Supplementary Table S2. Down-regulated gene lists (500 ea) based on LTT26 withthe most up-regulation of SFN. (LTT, Lung Tumor Tissue; LNT, Lung Normal Tissue)

Gene	LTT26 vs. LNT26	LTT52 vs. LNT52	LTT13 vs. LNT13	LTT17 vs. LNT17	LTT51 vs. LNT51	LTT12 vs. LNT12	LTT29 vs. LNT29
FCN3	-15.53550572	-14.70765367	-6.912602532	-6.556919006	-2.701738745	-6.650156822	-7.94517162
TIRAP	-11.59299129	0.317260132	0.318797248	0.748787205	-0.351858878	-0.809243876	-0.180485408
CTSG	-11.43677293	-3.296129667	-4.096112621	-7.817343683	-1.508341037	-2.600648527	-3.034011233
GIMAP1	-11.36877816	-3.72433824	-2.629367528	-2.564895553	-2.755234968	-6.626820898	-3.658742441
SVEP1	-11.30247091	-4.95000131	-2.467614417	-0.731471343	-4.762806996	-7.354731598	-2.564299147
ZNF571	-11.22083092	-0.078770077	0.151100115	-1.089439443	-0.433528018	-2.940462708	-0.76097219
ALDH1A1	-11.15339745	-2.421959705	-1.437525147	-2.912895167	-4.252011543	-1.632470262	-1.437152718
ABLIM1	-10.9539386	-1.648513042	-3.776593802	-1.335536469	-2.576515784	-4.376606988	-2.678925391
RAB5A	-10.94869652	-0.160867542	0.479512072	-0.126096459	-0.392931841	-3.181112009	-0.294410474
FCER1G	-10.5723862	-0.754587731	0.083170051	-1.160960994	-1.429658457	-2.480640633	-1.306754356
B3GNT6	-10.39526724	-1.020851173	-1.529892313	-1.996235476	-0.300882437	-2.032911728	-1.011510375
C14ORF128	-10.39126281	-1.275443414	-0.641343205	1.653004653	-1.998215245	-2.082282787	-0.412720168
WTAP	-10.32681689	0.055096023	-0.038770333	-0.769031553	-1.300043536	-3.408994844	-1.193922103
COLEC12	-10.24338499	0.588236347	-0.171617557	-0.816275049	0.60823775	-5.606262503	-1.032828198
DMRT2	-10.24251335	0.930337715	-3.566674876	-1.535416459	-1.280504659	-4.756863883	-2.914404543
TMEM1	-10.21196825	-0.515439043	-0.751970744	0.355965081	-0.336885414	-4.817179162	-0.901579856
C10ORF32	-10.13825152	-0.463842298	-0.666022126	-1.439037126	-0.771612398	-4.384796087	-1.290322435
SQLE	-10.11155624	-0.184935568	-0.987225303	0.673440028	-0.977828286	-2.573898218	-0.908410646
HIF1A	-10.09441834	3.233404236	1.377395189	1.331497573	0.701335786	-2.691124215	0.36625494
KCNE2	-10.08183574	0.698744097	1.470217106	-0.44614396	0.532757082	-0.032437098	0.187390206
H2AFV	-9.914080112	-1.565837447	-1.151041461	0.038270312	-1.224591417	-3.441078745	-1.187796655
C4BPA	-9.765806775	2.703385606	-0.824635666	-8.766079411	2.091495105	-5.565714179	-2.80411322
HS.545536	-9.740049168	2.433014156	-1.214335038	0.24882726	-1.768263571	-0.896992955	-0.219802904
SLC13A1	-9.702242086	-0.97170461	-2.459069081	-0.421906351	2.109322575	-2.38028646	-2.54627699
FABP4	-9.672257756	-9.0433568	-3.182454473	-4.338508089	-11.26139897	-11.2048116	-5.82509879
PDZRN4	-9.617798164	2.019637644	-0.869513457	0.165288918	0.848712772	0.532134161	1.515607409
SCGB1A1	-9.600357233	-9.522362978	1.318735397	-4.958967175	-4.535933799	-4.325165709	-7.918136105
MGAT4B	-9.525420954	0.671757385	-0.322939023	-1.494006508	-0.492544741	-4.195902792	-1.731049688
SIRT2	-9.517229102	-2.198578348	-0.997980591	-1.378623734	0.220835965	-4.033727219	-1.62472884
TNNC1	-9.496628208	-4.92956863	-7.871486018	-4.157616938	-4.028635071	-5.100787719	-5.683237768
LOC729217	-9.458276934	0.933499155	0.115485975	-1.23382589	-0.075522377	-3.374817037	-0.796120513
C11ORF1	-9.427866277	2.445172844	0.869981588	-0.488715456	1.17873578	-1.306389836	0.098818224
SRPX	-9.335327926	-5.453189604	-2.72246376	-1.500933212	-1.874009011	-7.694840395	-2.260590811
GPRC5A	-9.296498678	-1.181633477	-1.683190175	-1.959271853	-1.373161225	-6.339202492	-1.140312649
IL17D	-9.169210225	-4.44273211	-1.307468096	0.07430156	-2.403940474	-7.496588723	-1.010892256
EFEMP1	-9.096071157	-2.435453001	-2.129775102	-4.234724633	-1.287842182	-5.342822722	-2.746951008
FLJ32063	-8.991892073	0.460863632	3.85018769	0.097484024	0.418908701	-0.028959135	0.296093842
EEF1B2	-8.987321936	-0.104248906	-0.633748102	-2.067706939	-0.332107914	-3.74228897	-0.459528188
LOC402057	-8.959082812	0.658359343	-1.173572649	-5.445570531	0.523511132	-3.992582459	-0.654578885

CLDN18	-8.954657688	-6.826927695	-10.2432978	-11.30237197	-5.792124635	-7.79102021	-11.59059848
PLA2G1B	-8.876110782	-4.015420253	-6.8896056	-6.292919754	-5.082108446	-3.651687688	-12.34633382
FOLR1	-8.854634616	1.070964267	-1.239055346	-0.295607175	0.044414785	-0.912275387	-1.504716512
PDPK1	-8.811101531	-0.32430849	-0.489895963	-1.674161231	-0.308330088	-4.393129625	-1.558120507
CCL13	-8.796470829	0.387806941	1.136172179	1.201388683	1.348365785	0.298429421	0.193337673
METRNL	-8.772129382	-1.675764267	-1.126549325	-1.730001159	-1.029224171	-2.646748245	-5.079474878
EAF1	-8.761811749	-0.061946506	-0.319227024	-0.161445562	0.044017385	-3.364239851	-0.566337003
CUGBP2	-8.75854479	0.163793729	-1.116270483	-1.578376743	-1.438210292	-2.761303708	-2.453979979
LOC729920	-8.750931802	-4.001299691	-0.219232998	-0.922341109	-1.807238329	-3.170792453	-0.049321919
OSGIN2	-8.696313542	-0.73515511	-1.764768253	-1.940334051	-1.836111643	-5.184013604	-1.970079935
LIFR	-8.666640735	-5.733364342	-3.268498376	-2.989170645	-2.052742959	-3.963865305	-3.306137429
DHX29	-8.64481609	0.904956034	-0.343928405	-1.01807213	-0.11502384	-1.744275561	-0.047790016
CD48	-8.634584428	-0.722987859	-1.67420027	-2.766719911	0.134293116	-2.651987139	-3.332669872
CAV2	-8.597376793	-2.505432878	-3.627918831	-2.866389459	-4.886623551	-7.061679642	-4.430826583
CD9	-8.586532342	-0.617351	-0.22516866	-2.288581983	-1.296898436	-4.330023742	-0.241281594
LOC649143	-8.462155993	1.434107538	0.028530315	-1.45246016	0.80496463	-1.7194645	-2.917475369
ZBTB11	-8.416468234	0.421816321	-0.137993351	1.274229025	0.882263341	-3.90906098	-0.289566589
LOC391475	-8.350517092	0.520799036	-0.312746556	-0.271814379	-0.165183839	-1.305575562	1.998993468
LOC647081	-8.339295004	0.353819786	-0.352585195	-0.571783649	-1.103071364	-3.489344774	-0.004801313
ABI3BP	-8.312197874	-2.536619981	-3.797949201	-3.880681413	-4.446063233	-6.516260177	-4.606137154
CD37	-8.277461762	-1.571032196	-0.506802057	-1.432092818	-0.937050899	-2.439426375	-2.557643933
SEPP1	-8.25401476	-0.089662952	-1.292647074	-4.427269481	-1.987732963	-3.648460853	-1.938383195
KIF23	-8.22216732	0.816966094	1.551465142	1.770423882	-1.465069855	-0.70749404	0.172113393
CD74	-8.202431355	0.671178918	0.324422134	-0.484026233	-0.616044527	-1.829628812	-2.542746791
MMP7	-8.138502735	4.403569416	3.649809862	2.462493109	3.185737606	1.212373298	5.769193544
JAZF1	-8.115306974	-0.829798308	-0.483293367	-0.996666856	0.185628747	-2.631071222	-1.134425706
FAM190B	-8.111429154	-1.042209732	-0.842750665	-1.80932657	-2.091001221	-4.650449602	-0.920658361
PLAC8	-8.106040809	-2.93055226	-0.598645824	-0.8530585	-0.111603891	-5.602964648	-5.131558594
SPARCL1	-8.092991261	-3.811864122	-1.67222768	-2.345521916	-3.445086171	-5.809878101	-3.18548353
GOT1L1	-8.089942635	-1.087229234	-0.798808794	-0.901516012	-0.36854243	-0.645118233	0.110416899
HBA1	-8.08707375	-4.013613085	-6.116588182	-5.197952214	-3.452996836	-6.622965187	-4.799035767
TSPAN3	-8.078752147	0.856675635	0.11793111	-2.317487235	0.708011178	-2.46415869	-0.261367797
SORCS3	-8.044244748	-0.85093931	2.707294534	0.214056229	1.20922099	-0.344603108	1.504284988
HNRPK	-7.999579548	-0.091072874	0.130337658	-0.482222227	-0.279516306	-3.226780208	-0.913701538
TAPBP	-7.967056876	0.685332373	-0.688402448	0.46777593	0.558030427	-2.591321353	-1.016050752
TSPAN7	-7.95488947	-3.313729776	-3.580986538	-4.921571368	-4.769883315	-5.301209747	-4.936214364
MTMR14	-7.921442581	-0.836156255	-0.277699588	-0.565159534	-1.130940847	-1.596497724	-0.250414339
CYBRD1	-7.896070607	-2.454679994	-1.718979204	-1.929464439	-1.484555191	-5.263117803	-1.676102585
LOC392871	-7.894386982	0.788917008	0.960540768	-1.542064646	1.222369416	-4.217195533	0.971645171
CA2	-7.881844203	-2.505066224	-0.657659711	-6.159887949	-2.809384182	-3.608849637	-4.200952557
GLRX2	-7.881560526	0.60912794	-0.434005054	-0.758425327	-0.469758771	-1.493006511	0.384347935
TAP2	-7.870876252	-0.861407211	-0.898402541	0.579326115	0.235491114	-2.224280292	-1.451641458
LOC400759	-7.846693437	-1.459190046	-0.802235269	-0.472002232	-1.141851251	-2.753918098	-3.538122756

DIXDC1	-7.826657502	-4.608942953	-3.003932715	-3.009049132	-1.672399346	-5.839698028	-2.48749615
HLA-DQA1	-7.824467369	1.974980272	0.48641751	-1.366282858	0.359825226	-1.089430797	-2.697351372
PLEKHB2	-7.820554575	-0.303689203	-0.263721548	-0.758196136	-1.088444218	-3.450832839	-1.19800092
CPB2	-7.818888214	-2.064273282	-6.147541842	-3.776837939	-4.957216236	-9.253859085	-6.28026811
PGC	-7.790394126	-4.841614362	-7.971170909	-7.914210443	-4.562704156	-0.055884482	-8.846305459
NPNT	-7.78070911	-4.270258996	-3.859162015	-5.78924412	-4.56039666	-7.093993232	-3.191638637
MORN2	-7.757052693	2.522479024	0.441834625	-0.474112248	-1.075780037	-0.472405511	1.499818827
RPS24	-7.740009143	1.262994893	0.021627873	-0.749443266	0.952076902	-3.585057119	-0.144631081
TIMP3	-7.723548809	-3.233431842	-1.971615152	-0.769188323	-3.504809286	-5.509567142	-0.936067171
LOC441019	-7.722319644	-2.01077857	0.480248169	-0.95734784	-1.061723639	-3.15329224	-1.178125662
CAV1	-7.70745259	-4.591019835	-3.852811393	-4.103528653	-5.092138312	-6.572711348	-3.909760644
BMPR1A	-7.701211934	-0.065203553	-0.792567746	-0.410601652	-1.219574524	-4.886372157	-0.472721558
OLR1	-7.66613925	0.358759231	0.083166729	-0.949917355	-1.945892402	-4.38858417	-3.20588886
ZNF45	-7.659906865	-0.447434919	-1.067669616	-0.934698533	-0.268323076	-3.149548334	-0.438344902
IDO1	-7.64213673	-2.754612383	2.930316916	-0.555581934	1.452712178	-0.909173589	0.080690019
IFIT5	-7.616839788	-1.628786724	-1.067748611	0.188173604	-0.530208371	-3.50212774	-5.006551842
KLHL6	-7.612555187	-0.951682907	1.062476167	-0.943203726	-2.044003165	-3.612168937	-1.703346466
TRIM37	-7.610614432	-0.314793133	-1.620370611	1.301672041	-1.372622758	-1.876122574	0.020658178
LOC647444	-7.572129527	0.300166424	3.62794493	13.2315581	-0.999854788	9.206849601	-0.557684019
LOC729559	-7.555879596	-0.214691864	0.218416457	-0.45295899	-0.71271815	-1.640664534	0.014733987
NFASC	-7.555832128	-2.264571621	1.19273061	-0.649919918	-1.368187044	-1.126133222	-0.039582751
SLPI	-7.553561157	-1.249221741	-1.359472533	-2.067196576	-1.326995684	-6.567641937	-1.263378546
HS.434989	-7.51792954	3.320221135	0.155338116	0.511718927	2.149591989	-0.701665497	-0.960700814
EGFL6	-7.490538854	-3.334442434	-0.514795155	-1.564796284	-3.293949931	-4.080952767	-1.055909724
SEC23IP	-7.484463876	0.413079196	-0.120772174	0.499704773	-0.066571221	-1.791573125	-0.054691353
HS.146882	-7.471731987	-0.012649436	0.116912198	0.340077875	-0.450738931	-3.477540714	-3.26473015
WBSCR16	-7.453732189	-3.082247235	-0.91657678	6.369943635	-0.134114816	-2.446393008	0.419739813
LOC131185	-7.452484544	-0.540454585	-1.13292985	0.366885714	0.838321045	-0.041286642	0.369310333
FLJ27354	-7.443554518	-1.406491891	-0.388223941	1.34337805	-1.401730249	-4.827368786	-1.026052804
HS.571297	-7.430266964	-0.860703773	-0.24863224	2.651709375	-1.301245746	-1.588262321	-0.465594251
HS.356079	-7.421613528	0.203207348	-0.257137704	-0.470487027	-1.37686677	-2.37472168	-0.151397888
RAB43	-7.4056486	-0.202131051	-0.086946994	-0.120141584	1.014875041	-3.357850369	-0.168063617
FCGR1A	-7.404747066	-1.235824588	0.392464638	1.175025831	-0.624975227	-9.560289435	-0.917883533
SELS	-7.390026672	0.083194833	-0.128487108	-1.213171352	-0.221396091	-2.078967937	-0.161802069
LOC649389	-7.381269626	-0.492127455	1.421113931	0.408038816	-0.486803558	-0.533658001	-0.007000023
LOC401286	-7.377209852	-6.461778741	-5.640118183	-5.388508892	-5.764963455	-5.245065678	-5.39823581
CASP1	-7.364013751	0.188932901	-0.448058838	-1.28888222	0.367521536	-1.901168441	-0.816596805
C10ORF118	-7.357307813	-0.571003897	-0.796907338	-2.055523174	-0.245214396	-2.644305664	-0.257120452
DCN	-7.344656125	-3.502195154	-0.959819807	-2.096095521	-4.789358793	-5.894809834	-1.209213851
TEK	-7.332053683	-4.789501887	-3.19532302	-4.030126163	-2.265796386	-6.790049157	-4.539311317
IRS2	-7.330024571	-0.192009922	-0.778714668	-1.568325833	-0.33722764	-5.351477952	-1.889410884
VWA1	-7.327909003	1.401571586	0.666443605	1.16306937	1.215034952	-9.873401944	-0.183723505
MYADM	-7.300643372	-2.371585551	-1.117037939	-0.068586804	-3.127978603	-6.373020709	-3.298125038

C20ORF12	-7.299803557	-3.347336593	-0.658325908	4.067450263	-1.695881492	-1.299631237	-0.183609308
MSLN	-7.257249842	-3.91187543	-5.896265383	0.524027837	-1.776330866	-6.554955194	-0.679338005
LOC644544	-7.232242929	0.086194406	0.289942174	-1.643216825	0.107988524	-2.106655515	-0.863106248
ACADVL	-7.213617092	-1.105868401	0.040603582	-0.929052726	-0.643299745	-4.672419114	-1.357537424
C20ORF85	-7.213476619	-3.657168879	3.305315294	1.129652585	-2.700489958	-2.427487658	-1.396776295
LOC652633	-7.201735323	2.910790169	-1.493524184	-0.275596658	0.400993377	-0.944790026	0.375644427
KIF2A	-7.185612191	1.442136638	0.293496262	0.413774453	1.014301154	-1.092946422	0.549370745
SFTPA2B	-7.175492139	0.252858761	-2.860491337	-7.410353722	-0.087450203	-6.131765268	-4.255656608
CCDC101	-7.174217831	-2.55049511	-0.732863578	0.22360229	0.266897258	-1.326512435	-1.33495718
SPARCL1	-7.17223506	-3.947199873	-2.158874583	-2.504998862	-3.899382093	-5.592235779	-3.044409309
ITGA3	-7.165741885	-0.413528853	-0.497583385	-0.132915573	0.039729518	-1.331719484	-0.595171485
TGFBR2	-7.150905146	-1.619245384	-1.795359737	-2.018198095	-2.216208122	-4.772538328	-1.818772787
MS4A7	-7.143855854	-2.129485054	-0.288426145	-1.911031442	-2.515236607	-6.063343547	-3.313141671
KBTBD10	-7.123987362	0.161609519	-1.239631408	-1.32390706	2.067675988	-2.884482645	0.530744955
FPR3	-7.122668955	0.435902108	0.930409926	1.299093374	-1.090696329	-2.881041418	-1.374737784
UQCC	-7.11857686	-0.44714809	-1.176070044	-0.787740046	-0.597530913	-1.58247545	-0.755350261
TMEM16A	-7.113483314	-2.492271958	-1.759491705	-1.735419297	-1.232368884	-4.476378353	-2.036577423
ARAP2	-7.107259768	-0.884974659	-0.87950411	-0.721013634	-3.039562442	-2.496474076	-1.604514244
LYZ	-7.070503245	-0.456295441	0.293656768	-2.480111874	-2.004741316	-4.598067894	-3.010521584
SEPP1	-7.047651363	-0.220502205	-2.101211952	-1.531861464	-1.845595475	-3.468894331	-1.494191797
RRAS2	-7.043964435	0.106890308	0.105935501	0.323659772	0.490194072	-3.209350779	-0.910516012
SMAGP	-7.017430519	-0.820491514	-0.574010141	-1.02419074	1.27801129	-4.293031754	0.095509868
ENPP2	-7.015852557	-2.47357648	-1.864554387	-3.675638233	-3.50195526	-4.526909446	-2.357896391
LIPA	-7.013005308	-0.550949578	0.24397675	-1.873142904	-0.993220191	-3.029874362	-1.867123377
DPYD	-7.011897263	0.789326172	1.343733871	-2.731466169	0.740732106	-3.045125377	-0.904009646
KPNA3	-7.00029641	-0.290717296	-0.001409201	0.226230311	-0.970230445	-4.4433883	-0.646277461
RCL1	-6.998750221	-1.305758362	-1.828128704	-2.192774291	-1.040776244	-3.727284617	-1.981375428
DBC1	-6.985323168	1.593563633	4.410672947	-2.47141159	7.041080283	-0.858021583	-2.559336888
CFD	-6.9810252	-3.979348108	-1.862915011	-3.282186918	-3.252203287	-5.314031186	-4.250769575
SLC39A8	-6.965864938	-3.064207538	-2.068896131	-3.892609673	0.431357339	-4.26879231	-3.167968371
LCN9	-6.956481689	-0.570292643	0.47267437	-0.881758528	0.463975186	-1.79715408	1.118864337
FCN3	-6.954730048	-6.388785591	-5.208001048	-5.470247053	-2.277755852	-6.381135812	-6.907072273
CD151	-6.93478561	0.496802143	-0.287323862	0.185127988	-0.665312225	-3.164996797	-0.14691222
DSCR3	-6.927044031	-0.547831752	-1.25779526	-1.776063574	-0.626544102	-3.606595863	-1.691854437
C1ORF116	-6.926508233	0.365262392	-1.817091545	-0.118306941	-0.384648927	-1.923009547	-1.221795246
CYP4B1	-6.915518999	-4.424390324	-6.26159771	-7.105780889	-4.501106003	-4.892946121	-4.593905816
SLC25A4	-6.910652489	0.243015321	-0.427222834	-1.061902421	0.30508959	-2.300022628	-0.856095139
FCHO2	-6.902767344	0.722168276	0.106660435	0.353527013	0.727619404	-2.83144346	-0.764182436
LOC652815	-6.902270449	-1.127834129	-0.717736527	-0.802504988	-1.923446486	-2.783113405	-0.24662014
LOC648659	-6.900227393	0.08989168	-0.185154176	-0.101868719	0.786448683	-3.899960535	-0.759368689
MAP6	-6.899341334	-0.053210756	0.265207031	-0.481538527	-0.6063466	-6.110723941	-1.305343985
ANKRD37	-6.896658746	-1.863402396	-0.473578005	-0.675103678	0.75004096	-2.702160754	-2.071021937
VLDLR	-6.87690372	-2.715466753	-1.851023523	-0.151842965	-4.927530027	-3.092048263	-1.057698523

EI24	-6.872801294	0.044522937	-0.35798087	-0.125395303	0.726353143	-2.995819008	0.373639235
TMEM165	-6.86423581	1.701156905	1.543902368	-0.156394883	0.882465035	-2.995579338	0.60866422
HSD17B11	-6.860340695	0.605513572	0.505636766	-1.96381124	-0.079968762	-3.203816544	-2.045423287
SULT1A2	-6.827663192	-0.840582389	-2.513928363	-1.52684833	-0.943189681	-2.006015177	-1.356292811
ARSK	-6.824001212	0.287074852	-1.234044242	-0.977487713	-0.798064487	-1.495090857	-0.07628034
LOC728044	-6.821376079	-0.115092298	1.281342511	-0.399222743	2.336163666	-0.832937782	0.203225187
ITFG1	-6.820965866	0.697892483	0.31702274	-0.747510339	0.048695377	-2.8727934	-0.381394635
GPR177	-6.816294011	0.023939942	-1.188698341	-0.467449832	-0.055306212	-0.953818682	-0.132802638
TGFBR2	-6.805728461	-1.755740831	-1.626351879	-1.867414944	-1.761231354	-5.131583138	-2.288498136
CLEC4A	-6.798265222	0.393620337	-0.091917031	0.201785594	1.696211531	-2.373214706	-2.202836472
CST6	-6.795247926	-1.805923549	-0.758898177	1.438331036	-2.32608385	-9.005565692	1.659903118
C1QA	-6.786829611	-1.85277355	-0.49989001	-0.937635071	-2.209304796	-3.680610443	-1.620453954
NME7	-6.773068285	0.623737187	-0.459655317	-0.525155488	-0.678412669	-2.492965413	-0.379057754
IGJ	-6.771238621	-1.955700977	1.620985441	-6.477226523	-1.546342859	1.553346108	-1.821716493
C2ORF32	-6.750545598	-2.196033327	-1.448051767	-1.486399735	-3.334391371	-4.891786467	-1.032473706
LOC401845	-6.747221917	1.161065887	2.631111565	-2.732689149	-1.400239712	3.307569147	-0.14027199
LYVE1	-6.746871114	-8.097296232	-5.796305953	-6.744074144	-5.325250508	-7.179925895	-4.557047269
GPIHBP1	-6.739674093	-8.561895997	-6.439485311	-6.338002521	-4.727993759	-8.272173022	-11.94644988
HOPX	-6.738632497	-1.217004042	-0.852895217	-1.773905827	0.182778186	-6.014448784	-2.847975729
HPGD	-6.736953187	-3.28248377	-0.635601607	-2.204970457	2.934209379	-6.745419057	-3.545513181
HS6ST3	-6.72945815	0.741906611	1.55055477	-0.703022093	-0.664874629	-0.071232306	-0.557761985
GNG10	-6.715509895	0.270568225	0.107594912	-1.363965499	-0.039412736	-2.749040732	0.259408834
TMEM100	-6.714430482	-7.67735189	-5.422396903	-5.799820672	-4.455405255	-8.056520314	-7.196101767
HBB	-6.71207092	-3.071337461	-4.527558104	-4.694668732	-3.18175744	-5.404055012	-4.255755044
C17ORF58	-6.708820171	-0.555922129	-0.070568395	-1.076833733	0.499283526	-2.05014775	-0.55538035
C1ORF201	-6.705722903	-0.602364731	-1.229953357	0.080614402	-2.462871942	-1.254565665	2.388677776
COL12A1	-6.694752225	-1.991863523	1.036694856	2.710146082	-2.715520259	-4.240737304	2.713523923
C10ORF73	-6.694665494	-1.703487294	-1.121849165	-1.861105185	-0.674642355	-1.512491701	-0.478442447
C13ORF23	-6.669467323	0.214109544	-0.121715445	0.246956268	-0.007967501	-3.72449484	-1.252615365
CYP4B1	-6.660433503	-5.51842969	-6.029220797	-4.854561297	-4.735529	-4.132111705	-3.882814553
AGER	-6.6558329	-7.771408053	-8.532155084	-7.087769046	-4.872072815	-8.195611498	-8.589642051
UBXN2B	-6.655568118	0.244263764	-0.083799695	-0.199941958	-0.603170155	-2.81450562	-1.200609049
MGAT3	-6.652538581	-4.577524278	-3.8086069	-3.943244834	-2.508190174	-6.956459761	-4.936483079
LOC100132804	-6.651373674	0.145753116	0.128308123	-2.094727782	-0.5123153	-3.461051816	-0.315718277
LOC100133678	-6.648314977	1.909806959	0.784993154	-1.246530565	0.249659476	-1.265000104	-2.381135359
CRTAP	-6.640263574	-0.905380942	-1.061008166	-1.462265717	-0.930013295	-3.935147301	-1.613850129
LOC653463	-6.637455078	-4.603832182	-5.029638059	-4.347235454	-4.951926098	-5.465026119	-5.269309333
SMPDL3A	-6.625760911	1.770045754	0.987590323	-0.767418983	0.243104137	-2.514589856	0.34904302
DUOX1	-6.620911644	-2.702630478	-3.043347233	-3.338246409	-3.98259545	-0.034643281	-5.832155813
NME7	-6.615884786	0.880301043	-0.505747392	-0.198735715	-0.559137515	-2.294507198	-0.877463937
LRRN3	-6.591021624	-4.668407727	-3.354411111	-3.067933004	-3.519258718	-6.036591927	-2.822026961
NDUFV3	-6.564023858	0.575037591	-0.362259677	-0.812325049	-0.5669391	-2.905219283	-1.484505303
LOC730820	-6.558408361	0.109197085	0.112004207	-1.308533662	-1.072264414	-3.550007916	-1.550348573

SLK	-6.54620552	-0.403042844	0.421831385	-0.879823282	-0.040027309	-4.453486586	-0.995585724
CLIC3	-6.545893268	-1.981066897	-2.746936777	-1.625697117	-3.413728822	-5.748481594	-3.62927299
NUDT21	-6.544963956	-0.078360255	-0.693477257	-1.156479781	-1.31473734	-2.592995643	0.05922467
RPRD1B	-6.540617301	-0.747686209	-0.749929594	-0.280162257	0.084206619	-2.504880264	-0.526350154
RAP1GDS1	-6.532771673	0.352739358	0.147054012	0.697359696	0.100230887	-3.003634681	-0.052426614
C12ORF40	-6.531525242	#NUM!	-4.762477521	-1.306893263	1.882993468	0.542554149	-0.401952056
LOC149620	-6.526711191	-6.56481255	-5.905642491	-6.944578113	-3.585061953	-0.58891115	-2.910125874
HBA2	-6.523064861	-3.68251144	-4.471291154	-4.930629662	-3.39033204	-5.022235646	-3.662824762
PPIL6	-6.513083935	-1.867712285	0.030101611	0.594440894	-3.361132294	-2.466281673	-2.754546337
BCL7B	-6.504993239	-1.373063955	-1.412583301	-0.727000947	-0.247844727	-1.096782484	-0.651656509
LPL	-6.498754625	-3.235724111	-0.784301819	-1.82553133	-3.301997304	-6.975151251	-4.025825186
BAI1	-6.498444846	1.096902147	6.909138476	-2.805954842	1.767263179	0.742342323	0.524905336
RNU105C	-6.496066299	#NUM!	-0.846874443	1.661337211	-3.667850022	-2.375333306	0.006664375
LOC649555	-6.489146652	0.764623184	-0.079093024	-0.604339828	-0.001435124	-2.913136453	0.596234175
GKN2	-6.473420451	-5.923526152	-6.888591112	-5.730134246	-3.611186769	-9.457253465	-7.844306511
LOC729646	-6.465325941	0.904353738	0.684454829	-0.915055052	1.044478798	-2.67234747	0.462463433
HS.537004	-6.461211849	-0.728712073	-0.860931555	-0.724347303	-2.630632817	-3.617654	0.02049992
PQLC3	-6.458139907	-0.348013425	0.303350959	-0.953191589	-1.043201014	-2.911859806	0.147713906
MFAP4	-6.456057596	-4.566177965	-2.080276482	-2.800908235	-4.092475985	-5.822325789	-1.879043854
SRGN	-6.454811893	-1.500492787	-0.605307891	-2.997439066	-1.560948825	-4.077213786	-2.61581008
LAMP3	-6.45436144	-0.438494144	-3.443519791	-4.928671464	-1.653783739	-3.270320206	-5.870941283
SLC11A1	-6.443943582	-2.248664781	0.000680375	-0.833715918	-2.240328523	-5.944978706	-2.277381015
FHL1	-6.440297777	-5.945572623	-3.671703235	-4.106607705	-4.351380552	-6.626670878	-4.194343675
FEZ1	-6.439814597	-4.521427343	-2.487457633	-1.498758991	-4.015111138	-5.910675552	-2.06318416
TRIM5	-6.422466915	0.184290633	0.032998432	-0.392161196	0.164232661	-2.616126585	-0.049796345
HS.233165	-6.414066358	0.878536765	-1.37008113	0.242989794	#NUM!	-2.507897835	0.559580933
CCDC132	-6.412374841	0.029311525	-0.071185077	-1.020520091	-0.194258186	-2.786214464	0.503675304
FAM50B	-6.401667854	-0.330150746	0.169323081	-1.247192547	-0.061592443	-2.278852384	-0.621826121
MGAT4A	-6.399185427	0.59165871	1.082843999	-1.5604742	0.584148653	-3.450539533	-0.399679521
ARL3	-6.391103517	-2.592090017	-1.814180429	-0.599111765	-4.861454402	-9.080397495	-1.515424242
TXNDC5	-6.388613542	-0.262127322	0.747448706	-1.614325772	0.915848759	-1.96606684	-0.231444866
IL7R	-6.387165137	-3.238398416	-1.866712874	-0.008925118	-2.172105488	-3.585360168	-1.763806216
PRPF38B	-6.38659994	-0.660709027	-0.674653204	-0.182409144	-0.315419118	-2.587624397	-0.283410414
TLR7	-6.384004087	-0.709770507	-0.498188128	-0.515946725	-0.421199284	-4.199616078	-2.119955257
AGR3	-6.383952171	1.256253551	2.497663245	0.916272031	0.908700873	-3.007420571	0.419529171
HS.171169	-6.382015192	-0.332324784	2.016394056	0.055891532	-0.60447277	-0.305849036	0.822457864
FAM107A	-6.378830913	-7.268039417	-4.241351547	-8.231245117	-4.341290992	-9.254423081	-10.41379811
PECR	-6.37432983	-0.715657406	-1.675744871	-0.046905265	-0.167687536	-3.662580215	-1.153008847
LOC643873	-6.370178557	0.141485558	-0.783692437	-1.897770809	-0.668235716	-3.768145411	-0.885392582
PTPN13	-6.370060789	-0.200256363	-2.025881587	-1.957243532	0.232886775	-1.956058567	-0.811438845
MIR1225	-6.36481335	#NUM!	0.079534252	-1.992888458	-3.683846684	-1.098213497	-0.459890229
ZNF555	-6.36307906	0.573380137	-0.220469084	0.966925931	-1.554282557	-1.126660033	1.163847327
IGFBP6	-6.362681292	0.022525008	-2.49355597	-1.336611489	-2.557042087	-4.683035226	-1.757737572

LOC728843	-6.358396297	0.314054499	-0.749931708	-1.957426832	-0.455030639	-4.357785368	0.466117647
LOC100130746	-6.351642781	-0.3102833	-0.572733023	0.169445677	-0.524183929	-3.299009465	-0.789383502
RTN1	-6.350808722	-2.579281769	-0.744890535	-0.937912186	-0.568054594	-3.56635283	-4.472840139
TMX4	-6.344235154	-0.626982129	-0.115970276	-1.89619871	1.244988791	-1.7249441	-0.570362346
HS.583661	-6.338617859	0.628793182	1.659458281	0.936599689	0.03073004	-2.139389238	-1.287420103
CYFIP1	-6.336615699	-0.156835502	-0.3756215	-0.918178743	-0.159909695	-2.951098852	-0.415617654
LEPREL1	-6.3358107	1.294080363	-3.607033863	-2.865059283	-1.541871381	-3.786367816	-5.221297289
CHMP5	-6.334940271	-0.414780703	-0.535774376	-1.603960053	-0.822769586	-2.941679213	-0.401758568
AGER	-6.331796452	-5.265713157	-6.252789767	-7.082932864	-5.048365326	-7.725187331	-6.497402085
PGCP	-6.316838381	-0.351771605	-0.35778018	-1.085227935	-0.190307832	-2.998844406	-0.912971921
LOC728782	-6.307339752	0.21371745	-0.220118364	-2.093843947	-0.627459268	-3.930768113	-0.885510099
IL7R	-6.303954713	-3.30216909	-1.954064585	-0.369253328	-2.399211333	-3.876094259	-2.317619661
HLA-DPA1	-6.302360335	1.187423283	-0.101851746	-1.11105395	-0.132444457	-1.334335505	-2.430645491
MARCO	-6.30000109	-3.134384391	0.045644376	-1.185821888	-3.008295423	-5.146023402	-4.331442232
TSC22D1	-6.298966352	-1.332559036	-0.366419665	-2.717622361	-0.52937214	-6.285054199	-2.977184855
LOC100133055	-6.27368335	0.883085973	-0.929032045	1.553293209	-0.194828544	-2.375469617	-1.679797441
LACTB	-6.269487345	-0.076288502	0.2915961	-0.284869212	-0.31122386	-2.005056924	-0.613276627
CD163	-6.268741582	-2.102873632	-0.54011539	-1.961530984	-2.049862752	-3.565173657	-2.252296418
PPP2CB	-6.264807537	-0.172962338	-0.446722932	-0.981714652	-0.594125635	-4.770692725	-1.667721939
LOC100132060	-6.260921266	-1.617677958	-1.53267213	-1.811424027	-1.778318486	-4.090885772	-0.870251922
CCL23	-6.254855031	-1.299696145	-0.842176377	-3.30984615	-2.310202358	-5.675604625	-5.098213533
GPR34	-6.248029944	0.077128293	0.864674148	-0.764446361	0.029842556	-3.407978053	-0.820748003
ANXA3	-6.239376057	-2.596756723	-2.706636134	-1.884060849	-2.525150103	-6.421276011	-2.771028214
TCF21	-6.236978551	-6.103235648	-4.040678934	-4.31010639	-5.33977864	-7.333840235	-4.841306001
SFRS3	-6.236931427	-0.072572865	-0.559573087	-0.535968234	-0.299526917	-3.36039246	-0.019640728
HSD17B11	-6.236904857	0.577680007	0.525182772	-1.386504642	-0.434687776	-3.049548324	-1.940360095
XRCC5	-6.234833739	-0.022280977	0.094866638	-0.481276944	-0.276692444	-2.650472384	0.525347525
HLA-DRB4	-6.233128552	1.023296957	0.106464571	-1.307584349	0.376067051	-1.252177342	-2.612646424
LRRC59	-6.230131594	1.029272763	0.597217562	1.255045956	1.032719742	-2.090388842	0.125080047
RHOT1	-6.229302831	-0.684910105	-0.753938578	-1.239020978	-0.894261106	-2.739678088	-1.166420831
GRIN3B	-6.227735382	-2.449219359	2.141568603	1.382711958	-0.739532484	-2.790714717	-0.977394448
SP3	-6.225502267	1.458363286	-1.342108025	-2.105393999	-1.0949364	1.490669508	-0.101749544
CDC42	-6.224485383	0.894359335	-0.102490346	-2.826814684	-0.697478843	-3.360793259	-1.035816906
ZDHHC3	-6.221347837	0.202336265	-0.353148736	-0.885531674	-0.373515547	-3.098773519	-0.517677213
LOC100134384	-6.211616777	1.154439073	0.642497588	0.768051216	0.030115854	1.126964197	0.684574505
SPOCK2	-6.205156334	-3.82474658	-3.653316613	-4.052504594	-3.574674971	-4.766409957	-4.489532489
RRM2B	-6.191210732	2.062746451	0.272101928	1.231514323	0.460435321	-3.189178399	-0.145512549
FTO	-6.18923404	-0.248830741	0.046437451	-0.3862842	-0.416333646	-2.880889322	-0.158353933
OAS2	-6.186873222	-0.760764082	0.180217892	-0.268560169	-0.338000284	-2.129856	-1.285157897
DHRS12	-6.180839851	-0.772449328	-0.792241249	-0.809880746	-1.380881802	-1.403504522	0.102402868
DKFZP547K054	-6.175375325	#NUM!	-2.586317821	-1.73635791	1.12759392	-4.298942744	-0.095450416
KLF9	-6.171346943	-2.493445325	-1.709564371	-1.776545444	-1.958880019	-5.179704451	-3.122158275
TGFBR3	-6.167153153	-3.82839226	-3.480538151	-2.780800029	-1.636953818	-3.89553637	-5.704406903

IRX2	-6.165920701	0.343694671	-1.278691699	-1.465839597	-0.076680547	-0.99160296	-1.166150927
TM6SF1	-6.164847066	-0.830133041	-0.287647349	-2.417829716	-1.1703506	-5.85683987	-3.509036552
LOC389286	-6.157905451	-0.182958255	-0.535513653	-0.803907159	-1.11420172	1.451985044	0.890338477
FNTA	-6.148241189	0.209472807	-0.677676016	-0.796143533	-0.701576266	-3.262082861	-0.928176546
KITLG	-6.142874513	-0.707243843	-1.660655452	-3.456166613	-0.559282225	-4.514160256	-0.708827616
SFTPB	-6.139124571	0.905515115	0.313177091	-5.563510479	-0.542764382	-2.263346679	-5.949665555
RTKN	-6.133391079	0.756517212	#NUM!	-1.229018102	1.618972458	0.757001241	-0.140890091
OSCAR	-6.126718102	-2.780441969	-0.651919699	-1.777477616	-2.164222273	-3.827618672	-3.289387175
ICAM4	-6.122688631	-0.823477304	-2.375293806	1.268038349	-1.288405158	-4.281324301	-3.296020665
EPC1	-6.118718404	-1.368650686	-1.495090068	-1.832644533	-1.094279225	-3.436230387	-0.868232472
LOC255275	-6.114267221	-0.996908693	2.375013623	-0.752679935	-0.352933312	-2.828152673	0.653140901
CBR4	-6.112383601	-0.190694306	-0.291664704	0.317434315	0.305726098	-2.486039262	0.02905435
RNF144B	-6.101932353	-2.898124701	-2.32058036	-2.686819814	-1.989969892	-3.868825732	-0.501626804
AKAP11	-6.100287857	-1.031447328	-0.917010669	-0.972412574	-0.997806567	-4.841797279	-1.235609246
FAM150B	-6.099017411	-4.754403652	-3.225620453	-3.950610143	-5.933693577	-6.320483871	-4.9017398
COBL	-6.095920939	-1.408330219	-2.094321056	-1.079190557	-1.914080252	-3.268574525	-1.564251947
APP	-6.088573893	-0.570145341	-1.350458578	-0.651376058	-1.779959519	-3.38021183	-1.392170983
MYLK	-6.08114633	-2.902969601	-1.424161365	-0.851729119	-5.331429264	-5.97944347	-0.92283626
HS.539195	-6.077389651	1.029854675	0.335537133	0.759692758	0.245627712	3.394467112	0.162142872
VTA1	-6.072865085	-0.681338681	-0.394528879	-0.099377738	-0.991795972	-3.226197824	-0.294116528
LOC100128016	-6.070286877	-0.151963462	-0.883974347	0.842321647	-1.369654501	-2.374785356	-1.155519744
MAPKAP1	-6.065009933	0.192742679	-0.113043129	-0.460707301	-0.045176861	-2.816673088	-0.520325414
CHPT1	-6.059939088	-2.12941457	-1.622800041	-2.017118291	-0.531715921	-4.198885257	-1.824239076
ALOX5AP	-6.058326226	-0.840343319	-0.342754038	-1.193351048	-0.430506273	-3.158701624	-2.220186533
MAOA	-6.056934001	0.052678296	-1.407856289	-3.399968835	-0.869786979	-2.917981439	-2.879238968
NIPSNAP3A	-6.056834343	-0.656866144	-0.193340044	-0.046637035	-0.644413945	-3.182061417	-0.843912134
RSC1A1	-6.044422695	0.534852016	-1.285246373	1.133640311	1.751284627	-2.551806874	-1.709753844
LOC727821	-6.042924645	-0.337425396	-0.694343582	-1.697898231	-0.063979336	-3.614103861	-0.703556
CYBB	-6.0322945	-1.012553376	0.11056023	-0.849444218	-0.755352412	-3.074846661	-1.418044852
HS.551438	-6.028415274	-0.125459485	-0.591533927	-2.197508665	0.047176692	-2.593927626	-1.453088105
C21ORF37	-6.028069125	0.892819903	0.377309653	-0.752175706	-0.054840696	-2.651509232	0.983581072
ITM2B	-6.027572824	0.096014269	0.021345484	-1.009042183	-0.404122787	-3.357930832	-1.108511514
TMEM77	-6.021980567	-0.752773648	-0.193754077	-0.091703903	-0.653538979	-2.647188118	-0.529036781
HS.241559	-6.02053375	1.023847864	1.120866503	-0.163951579	-1.098752966	0.447230906	0.297806463
ARRDC2	-6.01587784	-0.88323242	-0.506151009	-1.510553185	1.124661933	-2.375530327	-1.421017869
WRB	-6.007201529	0.352805881	-0.464692715	-0.360251271	-0.385839594	-3.22508104	-0.66142014
DNM1L	-6.006772911	-0.252952161	-0.440267026	-0.96490925	-0.874992522	-3.503756414	-0.691108517
CITED2	-6.002464526	-0.449389133	-2.056623663	-2.775128279	0.191153292	-4.495443231	-2.294554288
TMPRSS3	-6.000110225	1.973266923	0.241981579	-0.536983022	-0.206513561	-0.667467646	0.170555849
HS.133009	-5.997556222	-4.806812442	-0.100092361	-2.199381187	0.292336824	-2.266881209	0.2228884
C14ORF144	-5.996942388	-0.388785097	-0.324952185	0.017517156	-0.26368076	1.340461213	-0.450545228
SFT2D3	-5.996287358	-1.607896489	-0.5475914	-0.219315958	0.703066646	-3.336444835	0.276680556
MIR1255B2	-5.980475688	0.036202252	0.206815914	4.208986307	-1.979016216	-1.451169503	-2.122111373

ZFAND6	-5.980394288	1.69491614	0.418751914	-1.284497495	-0.175763251	-3.18889466	-0.236514507
PIK3R1	-5.976405812	-5.488480727	-2.137480753	-5.230647512	-2.010135567	-3.639009446	-3.242544465
PPP2R2D	-5.972609383	-0.417115667	-0.885755509	-3.018934981	-1.056460567	-3.818354438	-2.428799103
HS.496187	-5.972114431	0.04507385	-1.263116131	-0.271091236	0.132905324	-0.137734903	-0.581892131
ATP2C1	-5.971324726	-0.196402811	-0.340098087	-1.157669661	-1.675106185	-3.585015387	-1.011020896
KIF18B	-5.969761782	0.531836236	1.438220212	0.003224732	-5.854049748	-1.402706056	0.427910408
LOC727900	-5.967010336	-0.571286524	-1.926583821	-0.540025187	-0.64004372	-2.274026272	-1.411987522
HS.545519	-5.966659609	0.265260869	-0.825596378	-0.58090773	0.864923782	-1.794366733	-0.049752966
SCGB3A1	-5.961820552	-0.200304738	1.705168052	-4.069830684	2.059274268	-1.609368538	1.453170231
MRE11A	-5.960424463	0.629547216	1.480680166	-0.400650682	0.05535219	-1.587420071	-0.193217593
SFTA2	-5.956180717	0.461507304	-0.018370318	-0.701917073	0.682377178	-1.543842422	-0.312020862
LOC728216	-5.952155331	-0.013806978	1.430981027	-1.090597671	2.926549727	-0.821098334	0.2872079
TMSB4X	-5.948267449	-1.352505028	-0.753914362	-0.340849776	-0.742058413	-3.25588904	-0.784385587
XKRY	-5.935960922	0.992302341	0.663322691	-1.077738568	-0.452432032	-3.016177837	-0.041114393
PSMD6	-5.93203698	0.294474321	-0.009527404	-0.22872862	-0.508166809	-2.997409952	-0.282789454
MAD2L1BP	-5.924395144	0.028954421	-0.08727343	-0.638269823	0.793072464	-1.355716292	-0.455998351
LOC649839	-5.917843308	0.654958586	0.371966168	-0.551393916	1.954254743	-2.602934971	0.610483555
C1ORF41	-5.91189835	1.235882706	0.235692521	-0.171928035	0.386619899	-0.666744699	0.421413612
NCKAP1	-5.906895346	0.036620965	0.661074682	-0.686076988	-1.017183411	-3.665169681	-0.489290559
LOC390748	-5.905848799	1.57431368	-0.626036937	0.106666888	-1.141231682	3.923173169	1.853704139
SFTA1P	-5.902697361	-1.851677512	-1.074682274	-3.269993338	-0.215991864	-3.557386716	-4.110803845
SRI	-5.901047733	-0.786648135	-0.768378633	-1.846839384	-0.239272776	-1.377020102	0.47766057
ZNF366	-5.900339756	-3.471673348	-1.854745742	-3.055448534	-2.172217244	-5.837951681	-3.300333623
TGFA	-5.896864634	3.630662375	2.741203029	0.807797313	0.645576339	-2.574216932	1.388567147
THOC3	-5.896472011	1.097307177	0.076004154	-0.808562495	0.168400073	-1.624349325	-0.459327627
CA4	-5.895077575	-7.191511612	-5.229630492	-6.858324019	-5.32007113	-6.906451089	-6.528139867
MMRN1	-5.89308858	-7.801575357	-4.208617221	-4.559249819	-6.859308842	-6.011800361	-4.243808589
PPAP2B	-5.891230127	-1.785019105	-1.551644714	-3.453837864	-1.800412782	-4.433035115	-2.452674666
LOC100131891	-5.891000142	0.674273023	-0.857313996	-1.212852666	5.653239067	-0.496163878	0.59857103
LOC730415	-5.888871915	0.79062668	0.171048942	-0.822400544	0.485822296	-0.944602192	-2.251287275
CSNK2A1	-5.876680383	-0.066081867	0.333133889	-0.962596128	-0.124357644	-1.94997717	-0.033432239
SERPINA1	-5.871255337	2.927831904	2.219907781	-0.748729218	1.083384284	-5.056454796	-0.269164871
CLEC12A	-5.859974254	-1.363046588	-0.032493217	-1.327933552	-3.404662664	-4.344067232	-3.245890228
MASTL	-5.854522883	0.622362749	-0.338975379	2.177544323	0.120914975	-1.399333896	-0.601408547
ALOX5	-5.852442582	-0.269121325	0.410938764	-0.844660073	-0.818143776	-3.323332726	-2.266374474
CENTB2	-5.844157186	-1.007136694	-0.278738877	-0.839375915	-0.780874053	-3.239231311	-0.831666692
HBEGF	-5.844022343	-2.318434209	-1.782259023	-2.852622321	-0.671909458	-5.756640332	-5.838401049
TLR8	-5.83240587	-2.151347838	-0.028443838	-1.74827087	-2.118956012	-4.500979556	-1.727943575
NOL4	-5.830682345	-2.925116458	-0.093919712	5.162811485	1.291933788	-0.097018061	-3.933050013
UTS2D	-5.824621826	0.280567488	-1.473385491	#NUM!	3.716685834	0.471807679	2.177484302
LOC653082	-5.823160674	-3.563722027	-1.959346431	-0.357574257	-2.862949526	-0.128108603	-0.405667794
RNF8	-5.810974197	-0.82757642	-0.590483176	-0.818017423	-0.440463502	-3.590041946	-0.677542814
B2M	-5.809577109	0.104707049	-0.350656534	-0.945839677	-0.971957138	-1.200597671	-0.896124375

TBC1D9	-5.808659788	-1.337479914	-1.439974675	-0.922849079	-1.593191692	-4.229361696	-1.520803304
BCKDHB	-5.805655455	0.194470535	0.32192678	-0.095530161	-0.924332605	-2.103323894	-0.867995881
SNX24	-5.80543368	-0.164669229	-0.569164419	-0.258696905	-0.713375831	-2.995640503	-1.564461251
LOC645946	-5.804941614	-1.09041322	-0.140519647	0.257981943	-0.460232833	3.907492994	1.065315806
GATA3	-5.803452661	-2.317341019	-2.444600872	0.152068335	-0.143824663	-1.822420345	-2.639490213
LOC441073	-5.801267432	0.127131799	-0.322639017	-2.649695716	-0.094081131	-3.383169329	-0.540141129
HS.105102	-5.79527886	-0.256647206	-0.373969517	-0.75641881	1.469934284	0.833824355	0.946606582
HLA-DRA	-5.79227036	0.626913338	0.305885266	-1.139557901	0.095852231	-1.150264825	-1.830358814
OR5L2	-5.792126175	-0.059912105	1.196896282	-0.173641495	0.527855262	0.209504674	1.317973477
PLK2	-5.790076818	-0.478479386	-1.317694815	-0.274687731	1.867025743	-3.368320338	-0.399445655
ETFB	-5.78323595	-0.937081707	-1.207509868	-1.180144818	0.117996088	-1.614428947	-0.701686832
VAMP7	-5.782487011	0.126119809	0.43781296	-0.227990289	-0.128382329	-3.146402226	-0.895362479
LOC128192	-5.78037246	0.363053975	-0.526802552	0.329777573	-0.189461489	-2.835927779	0.69087197
SHROOM3	-5.779621291	-0.131565868	-1.733876889	-0.512174255	0.142739655	-4.651800744	-0.526597235
LOC439994	-5.773672994	-1.396231473	-0.410198345	0.181976627	-0.69574748	-6.296722664	-1.930385358
ACTR3B	-5.7727131	-0.180915171	1.67103947	0.35146143	0.268278472	3.166474591	3.476803835
UBXN11	-5.768529116	-0.997749654	0.610555843	0.397267031	-0.311976496	-1.772033305	-0.713123582
CRYAB	-5.759337626	-3.854723615	-3.230544855	-1.791943976	-4.812947016	-5.411516617	-1.478254686
APOBEC3G	-5.751445684	-0.747243503	-0.196998053	-0.666103779	-0.244135129	-1.604734785	-1.012240749
IP6K2	-5.733182407	-0.553727606	0.186125582	-1.210266341	-0.711362406	-1.650945325	0.824612948
EIF1AX	-5.730295114	-0.532926476	-1.259037208	-0.189348768	-0.117009418	-3.672416337	-1.327839038
CD97	-5.73015524	-0.678890679	-1.057300812	-1.136525959	-0.362819802	-3.734278129	-2.805494867
SLCO2B1	-5.729191147	-1.299076278	-0.386977887	-0.592307935	-1.06765696	-4.118447046	-1.404752946
CD2AP	-5.72708013	0.7495152	0.441709406	-0.514238785	0.663383066	-3.004912645	0.41841045
TMEM188	-5.722010486	0.150589806	0.152918014	-1.378994131	-0.365947156	-3.357428703	-0.672019973
HS.242774	-5.720542454	#NUM!	-2.232373864	0.131916435	0.447615455	0.83974082	0.026275937
ING1	-5.716949327	-0.094367088	-0.176128968	-0.305188758	-0.829889921	-3.759859153	-1.211827653
HS.573763	-5.715372796	-0.420532118	0.251528478	-0.067061347	0.022581092	0.775077378	-0.559014099
DNAJB4	-5.714089534	-0.162241069	-0.706580319	-0.682451849	-2.306837704	-6.066659654	-2.070366454
S100A4	-5.71372842	-1.135293138	-1.000967463	-0.518250561	-1.314879263	-3.774682123	-0.94243121
ENPP2	-5.708458912	-2.841040827	-1.699764589	-4.168257457	-3.578209731	-4.783895842	-2.387306309
ADORA2B	-5.700873373	1.002786076	0.237186966	0.00335775	2.126241109	-1.397970436	1.878438333
ARF4	-5.697093151	1.432442538	0.705565199	-0.198708519	0.48650013	-2.549976256	0.491815102
SHE	-5.694299945	-2.496994892	-1.456081663	-2.812458794	0.781275288	-5.740644682	-2.09216797
FCER1A	-5.691236103	2.546956403	-0.116978576	-0.860235521	2.441152017	-3.069074941	-2.142163184
SDHD	-5.68948361	0.776524877	0.154952458	-1.226383098	0.288010394	-3.69959132	-0.853010694
FMO3	-5.686602267	-2.653811992	-0.873180775	-2.203607065	-3.739754659	-4.468614893	-0.308998751
TSC22D3	-5.686356487	-2.621380181	-1.903492648	-2.126476013	-1.306445142	-3.751087835	-2.347078986
CEBPA	-5.680338674	-0.37623257	-1.159147729	-1.190809762	-0.121964601	-1.831128077	-1.310331687
CTSH	-5.675402167	2.262387487	-0.843679568	-0.429733583	0.966306572	-0.282262711	-3.243342483
UTP14C	-5.674777427	-0.01355249	-0.277491818	-0.537262409	-0.304881471	-4.859743399	-0.542004637
MSRB3	-5.670260256	-3.95473042	-1.592102756	-1.247155952	-3.865935831	-7.040246868	-1.365322706
AHCYL2	-5.664164493	0.17834307	-0.252875142	-2.557411502	1.470626496	-0.138823348	-1.947011521

CD93	-5.663422155	-3.291200791	-2.391584139	-1.927522798	-2.788210228	-5.614548973	-2.624051658
SRGN	-5.65494633	-1.595670237	-0.798796509	-3.046227078	-1.670090954	-3.649447739	-2.851905691
ALPL	-5.649105909	1.999193418	-1.481343519	-5.33551427	1.111059922	-5.240650922	-4.079709917
CALCRL	-5.646230907	-4.055130506	-2.805014569	#NUM!	-3.623893425	-7.225861869	-3.248973418
HLA-DRB6	-5.643168709	0.91878663	0.303631573	-0.715307577	0.203120102	-0.782003506	-2.345065771
TYROBP	-5.639850713	-0.585969198	0.271983369	-0.914093222	-1.12795206	-2.700201164	-2.059999742
PGAM1	-5.639758546	0.076093481	-0.260429417	-0.927634823	-0.172710536	-2.9684681	-0.244823799
ST7	-5.63958858	-0.958744782	-0.319643985	-1.137625126	-0.20082618	-1.24905911	-0.171248812
CREG1	-5.638643618	0.55810311	-0.229568679	-1.025533116	-0.571647683	-1.389182667	-1.54580021
UBL3	-5.63627298	-0.73651478	-0.63752097	-0.684523414	-1.233375002	-4.798123744	-1.818821557
STAC3	-5.633606325	-1.594822791	-0.972279128	-4.839244556	1.188146441	-3.730197474	-3.41259141
LOC285016	-5.633421287	-4.812429686	-3.257231525	-8.481412803	-5.972125425	-11.57193393	-5.658242537
BTBD3	-5.626954023	-1.216142251	-1.31453516	-1.746846197	-0.819508138	-4.10858279	-1.467939976
PRMT3	-5.621983059	1.1725196	0.910644324	0.421322904	0.992578229	-2.046835486	0.275507348
LOC652614	-5.614830978	-0.480193727	-0.252825569	-0.236259786	-0.272383492	0.044731965	-1.499925069
HS.566669	-5.608310099	0.917258197	3.046155232	0.695126608	-0.538358051	-0.831219829	0.389112511
MTUS1	-5.603394026	0.648492848	0.994050375	-3.129983658	0.522434303	-2.429542822	0.057046449
CCL14	-5.598048667	-6.743660274	-5.117544804	-5.674626375	-4.370924884	-5.723228086	-4.580884973
BBS9	-5.597542805	-0.15303023	0.150679672	0.255220096	-0.239097874	-1.292800917	0.275588175
LOC441481	-5.59452091	1.431987782	1.568833174	0.46825588	0.642849968	-1.909792718	0.192361828
SLC46A2	-5.590218217	1.031354378	-0.571337716	-3.458889612	2.421055239	-6.492476229	-5.444490815
IRS1	-5.588983642	-1.330657932	-0.51771187	0.891091175	-0.019928634	-2.984235828	0.731351965
HLA-DRB4	-5.582913969	0.522116547	0.616409128	-1.119561259	0.391801833	-1.654565713	-2.140370061
GBP4	-5.581852971	-2.920391717	-2.379215423	-0.843444559	-2.763841922	-3.083065172	-4.707497669
C1ORF116	-5.580475935	-0.319447173	-3.214340751	-2.662389246	-1.213849326	-4.019762264	-3.234161072
PDIA6	-5.577484974	2.256448859	1.005096351	0.886979451	1.400598149	-0.895999866	0.519844627
EFCAB1	-5.573464129	-4.959550644	2.296856585	-2.057100316	-4.355487174	-2.793413179	-2.470984749
CXCL12	-5.572410467	-0.292306253	-1.43521649	0.317190856	-2.414106483	-5.986445807	-1.951522092
C4ORF31	-5.571855169	-0.193635181	-0.82385377	-2.21710399	0.235110244	-4.368774746	-1.604797217
SIK1	-5.562243713	-3.072065439	-1.460995301	-0.691918824	-2.065892215	-5.21176493	-3.929730118
C19ORF33	-5.562125671	-1.133422664	-0.478589044	0.834504843	-0.156128103	-3.203926186	1.041928366
MAP3K7	-5.551120355	-1.0187006	-0.441486713	0.03591406	-2.46815256	-5.756999215	-1.854867282
CD97	-5.550994004	-1.012814585	-1.272718118	-1.071384721	-0.436705214	-3.428508992	-3.240953241
LOC399942	-5.54368919	-0.343773231	-0.543099632	-1.788588939	-0.210394262	-2.925353145	-0.291762023
NR4A2	-5.543524401	-0.413035265	-0.470249514	-4.242841062	2.977078425	-6.618266811	-5.304061282
NTRK2	-5.543082913	2.51816353	#NUM!	-2.040256828	1.02728363	-1.380955806	1.766602591
CD86	-5.539590505	0.10599197	-0.365940678	-0.96929029	-1.599261005	-3.62649195	-3.134269866
TMEM17	-5.536242496	0.409902229	-0.492923324	-0.10514466	-1.223638447	-2.916707542	1.425258315
LOC440928	-5.531763779	-1.804276749	2.477722122	0.892361986	1.533740024	-3.061688055	1.670010967
PPM1D	-5.531266006	-1.444360219	-0.765315337	-1.926760607	-1.237111485	-3.877363996	-2.563349317
DEFB128	-5.529394821	0.472297588	0.273466662	1.285749961	0.051073686	-0.543917183	-0.625644952
CPVL	-5.528507852	-0.077983366	0.101592872	0.059220872	-0.307995215	-1.083014827	-2.878941011
DCTD	-5.528091733	0.215025909	-0.245702816	-0.886982133	0.871749168	-2.252225462	-0.020962629

APP	-5.527963813	-0.645682127	-1.359696312	-0.765400098	-1.526928974	-2.526120452	-0.871313292
44089	-5.527446007	-0.130738477	0.007554862	-0.55062696	0.071293297	-3.068138411	-0.370188301
IL15	-5.524710331	-0.580824258	-1.049069854	0.199729443	-1.024135039	-2.271459096	-0.059220358
HS.62314	-5.52393251	-1.140933204	-0.799032352	-1.126341772	-0.347505611	-4.361050027	-1.283460827
EPAS1	-5.523820084	-3.54492153	-2.5858621	-4.279373785	-2.772840352	-5.401628769	-3.628111635
RAB43	-5.521864194	-0.892149916	1.003815554	0.904515555	2.251349667	-1.765277922	-1.369364329
LOC100129773	-5.519723967	3.795903723	0.145707131	0.596016625	0.201631379	0.355563905	-0.283979559
CDK6	-5.51804945	-2.826277208	-1.607947192	-0.899292146	-1.246695131	-5.530473511	-1.315571474
PPARG	-5.517810588	-2.21983258	0.081017582	-1.165500828	-2.37168879	-6.213005412	-2.413502695
LGR5	-5.516124047	0.646595	-2.16619231	0.389976829	1.462228734	-0.411255901	1.344331654
PORCN	-5.511559342	-0.294929346	-0.464557761	0.850373721	-0.201198704	-0.418812854	0.664122456
GK	-5.51148608	-0.873000581	1.048315361	-1.482664637	2.915352183	-1.47518968	-2.150745751
CH25H	-5.511442192	1.274532141	0.059312836	-3.555394665	1.689439232	-5.085298595	-3.410784649
CLEC2B	-5.510823247	-1.345538958	-0.551585144	-0.562687787	-0.179818433	-3.990742297	-1.294268135
FBLN1	-5.509414265	-4.195887346	-1.262579203	-1.082838526	-4.173514486	-4.078352863	-0.87351262
DKFZP779M0652	-5.509373212	-1.019406186	0.943558984	-1.147766134	1.552043029	-1.38776498	-1.525584529
LOC389156	-5.506624245	0.26933208	-0.729758709	-2.222249365	-0.459420579	-3.938909648	-1.029290893
LOC100130511	-5.50320987	1.370966838	0.049862767	0.141177162	-0.008726804	-1.759938776	0.705338155
CD52	-5.50052344	-1.810667718	-0.858161847	-2.356194959	-2.119601726	-2.518594012	-2.630824662
C11ORF73	-5.496629579	1.030445784	0.05355788	0.32045809	-0.038549621	-2.968066047	-0.235639595
RNASEH2C	-5.494195357	-0.541176945	-0.400388893	0.519404221	1.990306938	-0.342729642	0.285622738
LOC100127894	-5.493615531	-0.717772521	0.378131821	1.223314426	-0.173273241	-1.404647446	0.321961632
ACOT9	-5.492185059	-1.021251823	-0.078377351	1.028551583	-0.586057156	-2.295699749	-0.409759831
LYZL1	-5.490549766	1.501885541	-0.068089607	0.259897693	-1.521441777	-0.29428225	-5.767882317
FLJ21986	-5.488833412	-2.924464226	-1.522262849	-1.329152186	-2.200244612	-5.594568071	-2.333955506
CASP1	-5.480059997	0.270365647	-0.446487829	-0.998390502	0.379876631	-1.91839897	-0.799147337
TMEM209	-5.476993892	0.299207227	0.221443905	-0.748515283	0.322185563	-1.977007831	-0.028496693
LOC100130351	-5.476803229	#NUM!	1.853496072	-3.657024861	0.033843473	-0.054251185	#NUM!
S100A4	-5.470414093	-0.976103097	-1.094293667	-0.737739313	-1.398940453	-4.043170726	-0.78518741
KRTAP10-8	-5.469857226	-0.967204264	0.59083432	-2.435540073	-0.596893985	-1.423058612	0.458751516
NKX2-2	-5.469067421	1.210928261	2.742415373	-2.056580731	6.432315681	-0.576193281	1.154639696
