# Supplementary material for: Stratifin (SFN) regulates lung cancer progression via nucleating the Vps34‐BECN1‐TRAF6 complex for autophagy induction
Source: Clin Transl Med. 2022 Jun 8;12(6):e896. doi: 10.1002/ctm2.896 (PMC9174881; doi:10.1002/ctm2.896)
Supplement: Supplementary file 4 — Supporting information [file CTM2-12-e896-s006.pdf]

Supplementary Table S3. Up-regulated genes in 7-tested LUAD patients. (LTT, Lung Tumor; Tissue LNT, Lung Normal Tissue)

Gene	LTT26 vs. LNT26	LTT52 vs. LNT52	LTT13 vs. LNT13	LTT17 vs. LNT17	LTT51 vs. LNT51	LTT12 vs. LNT12	LTT29 vs. LNT29
PHF19	8.707916533	1.102399077	1.743072395	1.923328076	2.147413807	3.734664011	2.388063636
MMP9	7.117217255	0.554773509	4.769965	0.511724598	3.594094847	3.871799099	4.069580647
SPDEF	6.585322948	5.010246532	5.982202466	11.3230767	5.793175056	2.712432874	5.817852534
BIRC5	6.332643843	1.559579462	1.166098843	1.949230484	0.385679856	2.166181556	2.193946678
SFN	6.164919562	4.85251918	4.673445344	4.128554909	3.863265966	3.442333083	3.392045174
SNORD3C	5.678166421	1.789854254	4.43502116	4.732406039	2.425453795	2.532939956	6.137116605
LOC728178	5.550781498	1.570400735	2.552706297	1.52217272	0.730746352	11.9260063	3.669158519
CEACAM18	5.443442891	1.043122545	1.065352881	0.324753442	2.437593341	0.645436104	0.0193468
TTK	5.282002689	2.272720925	1.429912875	1.397955308	0.054096649	1.361670449	2.158389384
SNORD3A	5.230300889	1.254870715	3.746173124	3.820800682	1.540005615	2.524994595	5.389801585
CA9	5.222037711	3.435790475	4.072996034	2.766525529	1.425871071	5.940020128	5.190460598
DDX11	5.119208634	0.014236098	2.423275041	0.467426435	1.341703676	0.32885802	0.617228856
SNORD3D	5.026604463	1.091762306	3.241769386	3.280032514	1.332214727	1.67362247	5.620424366
PODXL2	4.966751986	1.902649123	1.272888156	3.1297531	2.938579048	2.48323315	1.938690468
MESP1	4.61410956	4.206080235	2.435161774	0.290160566	2.622981361	3.647646804	4.819346702
UBE2C	4.548709059	5.160920936	2.548624652	3.380533634	2.109344831	1.223136628	3.867732138
ADAM8	4.349781449	3.990753976	2.571794003	2.825692227	5.942973956	2.573674428	0.445048667
UBE2C	4.345633664	3.304003271	2.49807153	2.890197655	2.480303591	1.000710382	3.10375351
RGS17	4.315526557	2.529387392	5.069999378	3.014683392	3.025343998	0.821949909	5.47508839
SLC29A4	4.242568233	0.987197482	1.123407476	1.358454709	1.287662939	1.129884762	1.968717138
LOC646993	4.145297267	2.592598253	4.90143763	3.764874342	0.764750147	0.644888499	3.840478609
LOC654342	4.137656934	1.169943119	0.079739447	1.470354921	0.457122725	0.139213022	1.567045241
TFAP2A	4.108089018	4.627790586	7.052621751	5.002351089	5.282324765	0.907654043	8.996207929
ASPM	4.107121954	1.923731046	2.561124806	2.683889737	0.912821893	0.012865401	2.176957302
C20ORF103	4.038528404	9.603894514	2.240948377	5.313210207	4.432312543	0.782496266	2.329120605
DTNB	3.908542855	0.311810601	2.411958589	2.753031817	2.099089292	1.447808927	2.577771953
AURKA	3.894845483	1.188626297	0.61542368	5.004398449	2.553099699	1.461907646	1.564821499
HS.145444	3.869792067	0.650433803	0.878702259	1.454152124	1.152979611	0.277478689	2.285475403
GALNT6	3.84639209	1.370944119	2.42925864	1.668378454	2.662091215	0.89904898	3.690532158
HS.568058	3.844220869	0.172940383	1.055386921	3.520832626	2.294174422	1.495187021	0.86772419
C15ORF54	3.834018276	3.138362797	2.327143115	0.946330976	3.924358857	0.183243477	0.585515659
KIFC1	3.782920543	1.079358514	2.204974057	3.5054001	2.685980399	1.344459512	1.973672146
PROM2	3.723995096	4.698463214	4.659595786	5.512191244	3.763547643	1.613252639	4.944779298
CCNF	3.624056383	1.730405749	1.361072838	1.180538011	1.441523179	0.140348786	1.187694239
FAM83A	3.580316098	3.519880512	4.971655964	6.113093892	1.506793975	3.186924007	6.410733001
ETV4	3.566510957	1.214601166	1.669677921	1.805075982	5.513216095	2.628710681	2.377141533
TOP2A	3.532901932	5.278075632	2.387677714	3.440318458	3.063068467	2.112843763	3.60998915
CDC20	3.476446495	3.241937712	1.486520389	3.262726263	1.899185734	1.900325522	3.096089223
CPT1B	3.410144464	0.642564073	0.526779028	1.041539675	1.49912999	1.888847925	2.375285391
TROAP	3.366499071	1.443635655	0.210036474	1.267318886	1.332517794	1.001506831	2.133442549

CAPN12	3.270842868	1.498558137	2.370684744	1.314747018	1.604808767	0.756851154	1.728175238
FLJ40113	3.193745897	1.465686351	0.31562328	0.844085727	1.114747831	0.671442452	0.625399759
EPHB3	3.130254591	2.559453151	1.531793202	3.533943014	5.16065952	1.073413119	3.204239537
SERINC2	3.079592099	3.012280183	1.98446753	2.735891047	2.257841708	1.027929147	2.593333095
C20ORF94	3.056981511	0.224785909	0.387366856	2.318114962	1.316423389	0.082437951	2.38745809
CDCA5	2.975188645	1.900106553	1.559711747	2.066138613	2.420515157	0.70534172	2.035413933
SLFN13	2.969204122	0.07914856	1.84293752	3.786465136	3.262329662	1.856729723	1.908175402
STX1A	2.939985832	1.587011266	4.024905322	2.177144122	2.795010548	0.859448861	4.665663623
PRC1	2.935466289	2.942754325	1.415612726	2.396459098	1.483971211	0.027698078	1.585619277
LOC440335	2.901882631	2.321547084	2.92320173	2.290006448	2.834371735	1.602617912	2.167543665
C6ORF141	2.859007805	3.559386676	3.729727316	1.928414407	4.012445078	2.888559823	1.838866636
HS.435263	2.847881491	0.823024405	2.339335668	1.387022157	3.554094109	0.286036975	2.800045273
ADM2	2.802116979	4.505824475	3.981268735	3.01910678	4.288334039	2.219354194	2.712233988
ETV4	2.784086857	1.625012114	1.097259629	2.250625035	4.802364517	2.230170481	2.706599203
C17ORF53	2.769941526	0.785353926	0.869054505	1.40288046	0.582343557	1.368268767	1.222345065
PYCR1	2.727879645	2.503292951	0.934768489	3.516918609	2.358864758	1.884133863	3.486658307
CHTF18	2.714024509	0.45605851	0.035973212	1.764142324	0.454536686	0.951143833	0.400379279
FAM178B	2.678847755	4.154988369	2.299896383	0.244248508	0.541925626	0.325274363	1.47296548
ADORA1	2.655434318	2.41466902	1.448549771	1.286700511	3.558002693	0.407241377	2.560765177
GSDMB	2.6459299	0.68615833	0.467260006	1.305504967	2.29187487	4.026917097	1.291584861
PAFAH1B3	2.63291619	1.421084746	1.645157137	2.10991228	1.216829765	1.748951111	2.298445059
PPP1R14D	2.627181325	2.446361768	0.946928187	0.750315242	3.15101376	3.054273634	1.231998408
PLEKHN1	2.627165293	1.632897926	0.573500198	2.936858057	1.849352164	0.576997688	1.597910623
NUSAP1	2.619274828	2.87256555	1.858529803	1.716175731	2.296581709	0.846712133	2.614595581
PC	2.540964041	2.859338217	4.777525716	1.423944243	5.919602191	5.501359193	1.042944893
CDKN2A	2.494461659	3.566032174	1.702141648	2.285788226	0.286472942	1.750463772	0.118172886
MELK	2.483768726	5.266143248	2.200463478	1.425773179	2.352545292	0.909090205	2.534795851
ASF1B	2.483382985	2.248433213	1.610446158	0.873984434	1.455707424	1.438644311	2.950812247
HLA-DQB2	2.448523469	4.613497362	5.258973465	2.029251352	3.458825466	1.268938588	0.507435052
FOXM1	2.445505348	1.299728039	1.102711393	5.289498576	0.943858655	1.65380419	2.932369532
SNORD59B	2.418459864	1.701629874	1.48692998	0.550341185	0.660788171	0.803003377	3.7215678
ALG1L	2.415454755	1.63198296	3.660895636	4.910252902	3.150248247	3.009022873	2.937616542
PABPC1L	2.41145571	1.709810961	1.820841238	3.351940511	0.979502084	1.830371748	1.349047418
PLXNB3	2.356808102	1.60863374	1.731376866	0.926463807	0.706002988	0.610126972	2.763078245
SIX4	2.356090835	1.904677919	5.700046756	5.091261123	3.122647293	1.617357294	4.395083919
LOC100133019	2.299491338	2.177565717	2.393178209	1.111522236	1.939485885	0.151525515	0.510812626
MCM2	2.262690593	1.813313436	0.701004229	1.084150698	3.09301386	0.833150319	1.900500344
