# Supplementary material for: Stratifin (SFN) regulates lung cancer progression via nucleating the Vps34‐BECN1‐TRAF6 complex for autophagy induction
Source: Clin Transl Med. 2022 Jun 8;12(6):e896. doi: 10.1002/ctm2.896 (PMC9174881; doi:10.1002/ctm2.896)
Supplement: Supplementary file 5 — Supporting information [file CTM2-12-e896-s009.pdf]

Supplementary Table S4. Down-regulated genes in 7-tested LUAD patients. (LTT, Lung Tumor Tissue; LNT, Lung Normal Tissue)

Gene	LTT26 vs. LNT26	LTT52 vs. LNT52	LTT13 vs. LNT13	LTT17 vs. LNT17	LTT51 vs. LNT51	LTT12 vs. LNT12	LTT29 vs. LNT29
FCN3	-15.53550572	-14.70765367	-6.912602532	-6.556919006	-2.701738745	-6.650156822	-7.94517162
CTSG	-11.43677293	-3.296129667	-4.096112621	-7.817343683	-1.508341037	-2.600648527	-3.034011233
GIMAP1	-11.36877816	-3.72433824	-2.629367528	-2.564895553	-2.755234968	-6.626820898	-3.658742441
SVEP1	-11.30247091	-4.95000131	-2.467614417	-0.731471343	-4.762806996	-7.354731598	-2.564299147
ALDH1A1	-11.15339745	-2.421959705	-1.437525147	-2.912895167	-4.252011543	-1.632470262	-1.437152718
ABLIM1	-10.9539386	-1.648513042	-3.776593802	-1.335536469	-2.576515784	-4.376606988	-2.678925391
B3GNT6	-10.39526724	-1.020851173	-1.529892313	-1.996235476	-0.300882437	-2.032911728	-1.011510375
C14ORF128	-10.39126281	-1.275443414	-0.641343205	1.653004653	-1.998215245	-2.082282787	-0.412720168
C10ORF32	-10.13825152	-0.463842298	-0.666022126	-1.439037126	-0.771612398	-4.384796087	-1.290322435
FABP4	-9.672257756	-9.0433568	-3.182454473	-4.338508089	-11.26139897	-11.2048116	-5.82509879
TNNC1	-9.496628208	-4.92956863	-7.871486018	-4.157616938	-4.028635071	-5.100787719	-5.683237768
SRPX	-9.335327926	-5.453189604	-2.72246376	-1.500933212	-1.874009011	-7.694840395	-2.260590811
GPRC5A	-9.296498678	-1.181633477	-1.683190175	-1.959271853	-1.373161225	-6.339202492	-1.140312649
EFEMP1	-9.096071157	-2.435453001	-2.129775102	-4.234724633	-1.287842182	-5.342822722	-2.746951008
EEF1B2	-8.987321936	-0.104248906	-0.633748102	-2.067706939	-0.332107914	-3.74228897	-0.459528188
CLDN18	-8.954657688	-6.826927695	-10.2432978	-11.30237197	-5.792124635	-7.79102021	-11.59059848
PLA2G1B	-8.876110782	-4.015420253	-6.8896056	-6.292919754	-5.082108446	-3.651687688	-12.34633382
PDPK1	-8.811101531	-0.32430849	-0.489895963	-1.674161231	-0.308330088	-4.393129625	-1.558120507
METRNL	-8.772129382	-1.675764267	-1.126549325	-1.730001159	-1.029224171	-2.646748245	-5.079474878
LOC729920	-8.750931802	-4.001299691	-0.219232998	-0.922341109	-1.807238329	-3.170792453	-0.049321919
OSGIN2	-8.696313542	-0.73515511	-1.764768253	-1.940334051	-1.836111643	-5.184013604	-1.970079935
LIFR	-8.666640735	-5.733364342	-3.268498376	-2.989170645	-2.052742959	-3.963865305	-3.306137429
CAV2	-8.597376793	-2.505432878	-3.627918831	-2.866389459	-4.886623551	-7.061679642	-4.430826583
CD9	-8.586532342	-0.617351	-0.22516866	-2.288581983	-1.296898436	-4.330023742	-0.241281594
ABI3BP	-8.312197874	-2.536619981	-3.797949201	-3.880681413	-4.446063233	-6.516260177	-4.606137154
CD37	-8.277461762	-1.571032196	-0.506802057	-1.432092818	-0.937050899	-2.439426375	-2.557643933
SEPP1	-8.25401476	-0.089662952	-1.292647074	-4.427269481	-1.987732963	-3.648460853	-1.938383195
FAM190B	-8.111429154	-1.042209732	-0.842750665	-1.80932657	-2.091001221	-4.650449602	-0.920658361
PLAC8	-8.106040809	-2.93055226	-0.598645824	-0.8530585	-0.111603891	-5.602964648	-5.131558594
SPARCL1	-8.092991261	-3.811864122	-1.67222768	-2.345521916	-3.445086171	-5.809878101	-3.18548353
HBA1	-8.08707375	-4.013613085	-6.116588182	-5.197952214	-3.452996836	-6.622965187	-4.799035767
TSPAN7	-7.95488947	-3.313729776	-3.580986538	-4.921571368	-4.769883315	-5.301209747	-4.936214364
MTMR14	-7.921442581	-0.836156255	-0.277699588	-0.565159534	-1.130940847	-1.596497724	-0.250414339
CYBRD1	-7.896070607	-2.454679994	-1.718979204	-1.929464439	-1.484555191	-5.263117803	-1.676102585
CA2	-7.881844203	-2.505066224	-0.657659711	-6.159887949	-2.809384182	-3.608849637	-4.200952557
LOC400759	-7.846693437	-1.459190046	-0.802235269	-0.472002232	-1.141851251	-2.753918098	-3.538122756
DIXDC1	-7.826657502	-4.608942953	-3.003932715	-3.009049132	-1.672399346	-5.839698028	-2.48749615
PLEKHB2	-7.820554575	-0.303689203	-0.263721548	-0.758196136	-1.088444218	-3.450832839	-1.19800092
CPB2	-7.818888214	-2.064273282	-6.147541842	-3.776837939	-4.957216236	-9.253859085	-6.28026811
PGC	-7.790394126	-4.841614362	-7.971170909	-7.914210443	-4.562704156	-0.055884482	-8.846305459

NPNT	-7.78070911	-4.270258996	-3.859162015	-5.78924412	-4.56039666	-7.093993232	-3.191638637
TIMP3	-7.723548809	-3.233431842	-1.971615152	-0.769188323	-3.504809286	-5.509567142	-0.936067171
CAV1	-7.70745259	-4.591019835	-3.852811393	-4.103528653	-5.092138312	-6.572711348	-3.909760644
BMPR1A	-7.701211934	-0.065203553	-0.792567746	-0.410601652	-1.219574524	-4.886372157	-0.472721558
ZNF45	-7.659906865	-0.447434919	-1.067669616	-0.934698533	-0.268323076	-3.149548334	-0.438344902
SLPI	-7.553561157	-1.249221741	-1.359472533	-2.067196576	-1.326995684	-6.567641937	-1.263378546
EGFL6	-7.490538854	-3.334442434	-0.514795155	-1.564796284	-3.293949931	-4.080952767	-1.055909724
LOC401286	-7.377209852	-6.461778741	-5.640118183	-5.388508892	-5.764963455	-5.245065678	-5.39823581
C10ORF118	-7.357307813	-0.571003897	-0.796907338	-2.055523174	-0.245214396	-2.644305664	-0.257120452
DCN	-7.344656125	-3.502195154	-0.959819807	-2.096095521	-4.789358793	-5.894809834	-1.209213851
TEK	-7.332053683	-4.789501887	-3.19532302	-4.030126163	-2.265796386	-6.790049157	-4.539311317
IRS2	-7.330024571	-0.192009922	-0.778714668	-1.568325833	-0.33722764	-5.351477952	-1.889410884
MYADM	-7.300643372	-2.371585551	-1.117037939	-0.068586804	-3.127978603	-6.373020709	-3.298125038
SPARCL1	-7.17223506	-3.947199873	-2.158874583	-2.504998862	-3.899382093	-5.592235779	-3.044409309
TGFBR2	-7.150905146	-1.619245384	-1.795359737	-2.018198095	-2.216208122	-4.772538328	-1.818772787
MS4A7	-7.143855854	-2.129485054	-0.288426145	-1.911031442	-2.515236607	-6.063343547	-3.313141671
UQCC	-7.11857686	-0.44714809	-1.176070044	-0.787740046	-0.597530913	-1.58247545	-0.755350261
TMEM16A	-7.113483314	-2.492271958	-1.759491705	-1.735419297	-1.232368884	-4.476378353	-2.036577423
ARAP2	-7.107259768	-0.884974659	-0.87950411	-0.721013634	-3.039562442	-2.496474076	-1.604514244
SEPP1	-7.047651363	-0.220502205	-2.101211952	-1.531861464	-1.845595475	-3.468894331	-1.494191797
ENPP2	-7.015852557	-2.47357648	-1.864554387	-3.675638233	-3.50195526	-4.526909446	-2.357896391
RCL1	-6.998750221	-1.305758362	-1.828128704	-2.192774291	-1.040776244	-3.727284617	-1.981375428
CFD	-6.9810252	-3.979348108	-1.862915011	-3.282186918	-3.252203287	-5.314031186	-4.250769575
FCN3	-6.954730048	-6.388785591	-5.208001048	-5.470247053	-2.277755852	-6.381135812	-6.907072273
DSCR3	-6.927044031	-0.547831752	-1.25779526	-1.776063574	-0.626544102	-3.606595863	-1.691854437
CYP4B1	-6.915518999	-4.424390324	-6.26159771	-7.105780889	-4.501106003	-4.892946121	-4.593905816
LOC652815	-6.902270449	-1.127834129	-0.717736527	-0.802504988	-1.923446486	-2.783113405	-0.24662014
VLDLR	-6.87690372	-2.715466753	-1.851023523	-0.151842965	-4.927530027	-3.092048263	-1.057698523
SULT1A2	-6.827663192	-0.840582389	-2.513928363	-1.52684833	-0.943189681	-2.006015177	-1.356292811
TGFBR2	-6.805728461	-1.755740831	-1.626351879	-1.867414944	-1.761231354	-5.131583138	-2.288498136
C1QA	-6.786829611	-1.85277355	-0.49989001	-0.937635071	-2.209304796	-3.680610443	-1.620453954
C2ORF32	-6.750545598	-2.196033327	-1.448051767	-1.486399735	-3.334391371	-4.891786467	-1.032473706
LYVE1	-6.746871114	-8.097296232	-5.796305953	-6.744074144	-5.325250508	-7.179925895	-4.557047269
GPIHBP1	-6.739674093	-8.561895997	-6.439485311	-6.338002521	-4.727993759	-8.272173022	-11.94644988
TMEM100	-6.714430482	-7.67735189	-5.422396903	-5.799820672	-4.455405255	-8.056520314	-7.196101767
HBB	-6.71207092	-3.071337461	-4.527558104	-4.694668732	-3.18175744	-5.404055012	-4.255755044
C10ORF73	-6.694665494	-1.703487294	-1.121849165	-1.861105185	-0.674642355	-1.512491701	-0.478442447
CYP4B1	-6.660433503	-5.51842969	-6.029220797	-4.854561297	-4.735529	-4.132111705	-3.882814553
AGER	-6.6558329	-7.771408053	-8.532155084	-7.087769046	-4.872072815	-8.195611498	-8.589642051
MGAT3	-6.652538581	-4.577524278	-3.8086069	-3.943244834	-2.508190174	-6.956459761	-4.936483079
CRTAP	-6.640263574	-0.905380942	-1.061008166	-1.462265717	-0.930013295	-3.935147301	-1.613850129
LOC653463	-6.637455078	-4.603832182	-5.029638059	-4.347235454	-4.951926098	-5.465026119	-5.269309333
DUOX1	-6.620911644	-2.702630478	-3.043347233	-3.338246409	-3.98259545	-0.034643281	-5.832155813
LRRN3	-6.591021624	-4.668407727	-3.354411111	-3.067933004	-3.519258718	-6.036591927	-2.822026961

CLIC3	-6.545893268	-1.981066897	-2.746936777	-1.625697117	-3.413728822	-5.748481594	-3.62927299
LOC149620	-6.526711191	-6.56481255	-5.905642491	-6.944578113	-3.585061953	-0.58891115	-2.910125874
HBA2	-6.523064861	-3.68251144	-4.471291154	-4.930629662	-3.39033204	-5.022235646	-3.662824762
BCL7B	-6.504993239	-1.373063955	-1.412583301	-0.727000947	-0.247844727	-1.096782484	-0.651656509
LPL	-6.498754625	-3.235724111	-0.784301819	-1.82553133	-3.301997304	-6.975151251	-4.025825186
GKN2	-6.473420451	-5.923526152	-6.888591112	-5.730134246	-3.611186769	-9.457253465	-7.844306511
MFAP4	-6.456057596	-4.566177965	-2.080276482	-2.800908235	-4.092475985	-5.822325789	-1.879043854
SRGN	-6.454811893	-1.500492787	-0.605307891	-2.997439066	-1.560948825	-4.077213786	-2.61581008
LAMP3	-6.45436144	-0.438494144	-3.443519791	-4.928671464	-1.653783739	-3.270320206	-5.870941283
FHL1	-6.440297777	-5.945572623	-3.671703235	-4.106607705	-4.351380552	-6.626670878	-4.194343675
FEZ1	-6.439814597	-4.521427343	-2.487457633	-1.498758991	-4.015111138	-5.910675552	-2.06318416
ARL3	-6.391103517	-2.592090017	-1.814180429	-0.599111765	-4.861454402	-9.080397495	-1.515424242
IL7R	-6.387165137	-3.238398416	-1.866712874	-0.008925118	-2.172105488	-3.585360168	-1.763806216
PRPF38B	-6.38659994	-0.660709027	-0.674653204	-0.182409144	-0.315419118	-2.587624397	-0.283410414
TLR7	-6.384004087	-0.709770507	-0.498188128	-0.515946725	-0.421199284	-4.199616078	-2.119955257
FAM107A	-6.378830913	-7.268039417	-4.241351547	-8.231245117	-4.341290992	-9.254423081	-10.41379811
PECR	-6.37432983	-0.715657406	-1.675744871	-0.046905265	-0.167687536	-3.662580215	-1.153008847
RTN1	-6.350808722	-2.579281769	-0.744890535	-0.937912186	-0.568054594	-3.56635283	-4.472840139
CYFIP1	-6.336615699	-0.156835502	-0.3756215	-0.918178743	-0.159909695	-2.951098852	-0.415617654
CHMP5	-6.334940271	-0.414780703	-0.535774376	-1.603960053	-0.822769586	-2.941679213	-0.401758568
AGER	-6.331796452	-5.265713157	-6.252789767	-7.082932864	-5.048365326	-7.725187331	-6.497402085
PGCP	-6.316838381	-0.351771605	-0.35778018	-1.085227935	-0.190307832	-2.998844406	-0.912971921
IL7R	-6.303954713	-3.30216909	-1.954064585	-0.369253328	-2.399211333	-3.876094259	-2.317619661
TSC22D1	-6.298966352	-1.332559036	-0.366419665	-2.717622361	-0.52937214	-6.285054199	-2.977184855
CD163	-6.268741582	-2.102873632	-0.54011539	-1.961530984	-2.049862752	-3.565173657	-2.252296418
PPP2CB	-6.264807537	-0.172962338	-0.446722932	-0.981714652	-0.594125635	-4.770692725	-1.667721939
LOC100132060	-6.260921266	-1.617677958	-1.53267213	-1.811424027	-1.778318486	-4.090885772	-0.870251922
CCL23	-6.254855031	-1.299696145	-0.842176377	-3.30984615	-2.310202358	-5.675604625	-5.098213533
ANXA3	-6.239376057	-2.596756723	-2.706636134	-1.884060849	-2.525150103	-6.421276011	-2.771028214
TCF21	-6.236978551	-6.103235648	-4.040678934	-4.31010639	-5.33977864	-7.333840235	-4.841306001
SFRS3	-6.236931427	-0.072572865	-0.559573087	-0.535968234	-0.299526917	-3.36039246	-0.019640728
RHOT1	-6.229302831	-0.684910105	-0.753938578	-1.239020978	-0.894261106	-2.739678088	-1.166420831
SPOCK2	-6.205156334	-3.82474658	-3.653316613	-4.052504594	-3.574674971	-4.766409957	-4.489532489
KLF9	-6.171346943	-2.493445325	-1.709564371	-1.776545444	-1.958880019	-5.179704451	-3.122158275
TGFBR3	-6.167153153	-3.82839226	-3.480538151	-2.780800029	-1.636953818	-3.89553637	-5.704406903
TM6SF1	-6.164847066	-0.830133041	-0.287647349	-2.417829716	-1.1703506	-5.85683987	-3.509036552
KITLG	-6.142874513	-0.707243843	-1.660655452	-3.456166613	-0.559282225	-4.514160256	-0.708827616
OSCAR	-6.126718102	-2.780441969	-0.651919699	-1.777477616	-2.164222273	-3.827618672	-3.289387175
EPC1	-6.118718404	-1.368650686	-1.495090068	-1.832644533	-1.094279225	-3.436230387	-0.868232472
RNF144B	-6.101932353	-2.898124701	-2.32058036	-2.686819814	-1.989969892	-3.868825732	-0.501626804
AKAP11	-6.100287857	-1.031447328	-0.917010669	-0.972412574	-0.997806567	-4.841797279	-1.235609246
FAM150B	-6.099017411	-4.754403652	-3.225620453	-3.950610143	-5.933693577	-6.320483871	-4.9017398
COBL	-6.095920939	-1.408330219	-2.094321056	-1.079190557	-1.914080252	-3.268574525	-1.564251947
APP	-6.088573893	-0.570145341	-1.350458578	-0.651376058	-1.779959519	-3.38021183	-1.392170983

MYLK	-6.08114633	-2.902969601	-1.424161365	-0.851729119	-5.331429264	-5.97944347	-0.92283626
VTA1	-6.072865085	-0.681338681	-0.394528879	-0.099377738	-0.991795972	-3.226197824	-0.294116528
CHPT1	-6.059939088	-2.12941457	-1.622800041	-2.017118291	-0.531715921	-4.198885257	-1.824239076
ALOX5AP	-6.058326226	-0.840343319	-0.342754038	-1.193351048	-0.430506273	-3.158701624	-2.220186533
MAOA	-6.056934001	0.052678296	-1.407856289	-3.399968835	-0.869786979	-2.917981439	-2.879238968
NIPSNAP3A	-6.056834343	-0.656866144	-0.193340044	-0.046637035	-0.644413945	-3.182061417	-0.843912134
LOC727821	-6.042924645	-0.337425396	-0.694343582	-1.697898231	-0.063979336	-3.614103861	-0.703556
TMEM77	-6.021980567	-0.752773648	-0.193754077	-0.091703903	-0.653538979	-2.647188118	-0.529036781
DNM1L	-6.006772911	-0.252952161	-0.440267026	-0.96490925	-0.874992522	-3.503756414	-0.691108517
PIK3R1	-5.976405812	-5.488480727	-2.137480753	-5.230647512	-2.010135567	-3.639009446	-3.242544465
PPP2R2D	-5.972609383	-0.417115667	-0.885755509	-3.018934981	-1.056460567	-3.818354438	-2.428799103
ATP2C1	-5.971324726	-0.196402811	-0.340098087	-1.157669661	-1.675106185	-3.585015387	-1.011020896
LOC727900	-5.967010336	-0.571286524	-1.926583821	-0.540025187	-0.64004372	-2.274026272	-1.411987522
TMSB4X	-5.948267449	-1.352505028	-0.753914362	-0.340849776	-0.742058413	-3.25588904	-0.784385587
SFTA1P	-5.902697361	-1.851677512	-1.074682274	-3.269993338	-0.215991864	-3.557386716	-4.110803845
ZNF366	-5.900339756	-3.471673348	-1.854745742	-3.055448534	-2.172217244	-5.837951681	-3.300333623
CA4	-5.895077575	-7.191511612	-5.229630492	-6.858324019	-5.32007113	-6.906451089	-6.528139867
MMRN1	-5.89308858	-7.801575357	-4.208617221	-4.559249819	-6.859308842	-6.011800361	-4.243808589
PPAP2B	-5.891230127	-1.785019105	-1.551644714	-3.453837864	-1.800412782	-4.433035115	-2.452674666
CLEC12A	-5.859974254	-1.363046588	-0.032493217	-1.327933552	-3.404662664	-4.344067232	-3.245890228
CENTB2	-5.844157186	-1.007136694	-0.278738877	-0.839375915	-0.780874053	-3.239231311	-0.831666692
HBEGF	-5.844022343	-2.318434209	-1.782259023	-2.852622321	-0.671909458	-5.756640332	-5.838401049
TLR8	-5.83240587	-2.151347838	-0.028443838	-1.74827087	-2.118956012	-4.500979556	-1.727943575
LOC653082	-5.823160674	-3.563722027	-1.959346431	-0.357574257	-2.862949526	-0.128108603	-0.405667794
RNF8	-5.810974197	-0.82757642	-0.590483176	-0.818017423	-0.440463502	-3.590041946	-0.677542814
B2M	-5.809577109	0.104707049	-0.350656534	-0.945839677	-0.971957138	-1.200597671	-0.896124375
TBC1D9	-5.808659788	-1.337479914	-1.439974675	-0.922849079	-1.593191692	-4.229361696	-1.520803304
SNX24	-5.80543368	-0.164669229	-0.569164419	-0.258696905	-0.713375831	-2.995640503	-1.564461251
CRYAB	-5.759337626	-3.854723615	-3.230544855	-1.791943976	-4.812947016	-5.411516617	-1.478254686
APOBEC3G	-5.751445684	-0.747243503	-0.196998053	-0.666103779	-0.244135129	-1.604734785	-1.012240749
EIF1AX	-5.730295114	-0.532926476	-1.259037208	-0.189348768	-0.117009418	-3.672416337	-1.327839038
CD97	-5.73015524	-0.678890679	-1.057300812	-1.136525959	-0.362819802	-3.734278129	-2.805494867
SLCO2B1	-5.729191147	-1.299076278	-0.386977887	-0.592307935	-1.06765696	-4.118447046	-1.404752946
ING1	-5.716949327	-0.094367088	-0.176128968	-0.305188758	-0.829889921	-3.759859153	-1.211827653
DNAJB4	-5.714089534	-0.162241069	-0.706580319	-0.682451849	-2.306837704	-6.066659654	-2.070366454
S100A4	-5.71372842	-1.135293138	-1.000967463	-0.518250561	-1.314879263	-3.774682123	-0.94243121
ENPP2	-5.708458912	-2.841040827	-1.699764589	-4.168257457	-3.578209731	-4.783895842	-2.387306309
FMO3	-5.686602267	-2.653811992	-0.873180775	-2.203607065	-3.739754659	-4.468614893	-0.308998751
TSC22D3	-5.686356487	-2.621380181	-1.903492648	-2.126476013	-1.306445142	-3.751087835	-2.347078986
CEBPA	-5.680338674	-0.37623257	-1.159147729	-1.190809762	-0.121964601	-1.831128077	-1.310331687
UTP14C	-5.674777427	-0.01355249	-0.277491818	-0.537262409	-0.304881471	-4.859743399	-0.542004637
MSRB3	-5.670260256	-3.95473042	-1.592102756	-1.247155952	-3.865935831	-7.040246868	-1.365322706
CD93	-5.663422155	-3.291200791	-2.391584139	-1.927522798	-2.788210228	-5.614548973	-2.624051658
SRGN	-5.65494633	-1.595670237	-0.798796509	-3.046227078	-1.670090954	-3.649447739	-2.851905691

ST7	-5.63958858	-0.958744782	-0.319643985	-1.137625126	-0.20082618	-1.24905911	-0.171248812
UBL3	-5.63627298	-0.73651478	-0.63752097	-0.684523414	-1.233375002	-4.798123744	-1.818821557
LOC285016	-5.633421287	-4.812429686	-3.257231525	-8.481412803	-5.972125425	-11.57193393	-5.658242537
BTBD3	-5.626954023	-1.216142251	-1.31453516	-1.746846197	-0.819508138	-4.10858279	-1.467939976
CCL14	-5.598048667	-6.743660274	-5.117544804	-5.674626375	-4.370924884	-5.723228086	-4.580884973
GBP4	-5.581852971	-2.920391717	-2.379215423	-0.843444559	-2.763841922	-3.083065172	-4.707497669
C1ORF116	-5.580475935	-0.319447173	-3.214340751	-2.662389246	-1.213849326	-4.019762264	-3.234161072
SIK1	-5.562243713	-3.072065439	-1.460995301	-0.691918824	-2.065892215	-5.21176493	-3.929730118
CD97	-5.550994004	-1.012814585	-1.272718118	-1.071384721	-0.436705214	-3.428508992	-3.240953241
LOC399942	-5.54368919	-0.343773231	-0.543099632	-1.788588939	-0.210394262	-2.925353145	-0.291762023
PPM1D	-5.531266006	-1.444360219	-0.765315337	-1.926760607	-1.237111485	-3.877363996	-2.563349317
APP	-5.527963813	-0.645682127	-1.359696312	-0.765400098	-1.526928974	-2.526120452	-0.871313292
HS.62314	-5.52393251	-1.140933204	-0.799032352	-1.126341772	-0.347505611	-4.361050027	-1.283460827
EPAS1	-5.523820084	-3.54492153	-2.5858621	-4.279373785	-2.772840352	-5.401628769	-3.628111635
CDK6	-5.51804945	-2.826277208	-1.607947192	-0.899292146	-1.246695131	-5.530473511	-1.315571474
CLEC2B	-5.510823247	-1.345538958	-0.551585144	-0.562687787	-0.179818433	-3.990742297	-1.294268135
FBLN1	-5.509414265	-4.195887346	-1.262579203	-1.082838526	-4.173514486	-4.078352863	-0.87351262
CD52	-5.50052344	-1.810667718	-0.858161847	-2.356194959	-2.119601726	-2.518594012	-2.630824662
FLJ21986	-5.488833412	-2.924464226	-1.522262849	-1.329152186	-2.200244612	-5.594568071	-2.333955506
S100A4	-5.470414093	-0.976103097	-1.094293667	-0.737739313	-1.398940453	-4.043170726	-0.78518741
