# Supplementary material for: Stratifin (SFN) regulates lung cancer progression via nucleating the Vps34‐BECN1‐TRAF6 complex for autophagy induction
Source: Clin Transl Med. 2022 Jun 8;12(6):e896. doi: 10.1002/ctm2.896 (PMC9174881; doi:10.1002/ctm2.896)
Supplement: Supplementary file 6 — Supporting information [file CTM2-12-e896-s001.pdf]

Supplementary Table S5. Up-regulated genes related to cancer proliferation in lung tumor tissues

(LTT, Lung Tumor Tissue; LNT, Lung Normal Tissue)

Gene	LTT26 vs. LNT26	LTT52 vs. LNT52	LTT13 vs. LNT13	LTT17 vs. LNT17	LTT51 vs. LNT51	LTT12 vs. LNT12	LTT29 vs. LNT29
SFN[1,*]	6.164919562	4.85251918	4.673445344	4.128554909	3.863265966	3.442333083	3.392045174
UBE2C[2,*]	4.548709059	5.160920936	2.548624652	3.380533634	2.109344831	1.223136628	3.867732138
ASPM[3*]	4.107121954	1.923731046	2.561124806	2.683889737	0.912821893	0.012865401	2.176957302
AURKA[4*]	3.894845483	1.188626297	0.61542368	5.004398449	2.553099699	1.461907646	1.564821499
FAM83A[5*]	3.580316098	3.519880512	4.971655964	6.113093892	1.506793975	3.186924007	6.410733001
ETV4[6*]	3.566510957	1.214601166	1.669677921	1.805075982	5.513216095	2.628710681	2.377141533
TOP2A[7*]	3.532901932	5.278075632	2.387677714	3.440318458	3.063068467	2.112843763	3.60998915
SERINC2[8*]	3.079592099	3.012280183	1.98446753	2.735891047	2.257841708	1.027929147	2.593333095
PRC1[9*]	2.935466289	2.942754325	1.415612726	2.396459098	1.483971211	0.027698078	1.585619277
NUSAP1[10*]	2.619274828	2.87256555	1.858529803	1.716175731	2.296581709	0.846712133	2.614595581
ASF1B[11*]	2.483382985	2.248433213	1.610446158	0.873984434	1.455707424	1.438644311	2.950812247
MELK[12]	2.483768726	5.266143248	2.200463478	1.425773179	2.352545292	0.909090205	2.534795851
PHF19[13]	8.707916533	1.102399077	1.743072395	1.923328076	2.147413807	3.734664011	2.388063636
BIRC5[14]	6.332643843	1.559579462	1.166098843	1.949230484	0.385679856	2.166181556	2.193946678
TTK[15]	5.282002689	2.272720925	1.429912875	1.397955308	0.054096649	1.361670449	2.158389384
DDX11[16]	5.119208634	0.014236098	2.423275041	0.467426435	1.341703676	0.32885802	0.617228856
TROAP[17]	3.366499071	1.443635655	0.210036474	1.267318886	1.332517794	1.001506831	2.133442549
CDC20[18]	3.476446495	3.241937712	1.486520389	3.262726263	1.899185734	1.900325522	3.096089223
KIFC1[19]	3.782920543	1.079358514	2.204974057	3.5054001	2.685980399	1.344459512	1.973672146
CDCA5[20]	2.975188645	1.900106553	1.559711747	2.066138613	2.420515157	0.70534172	2.035413933
PYCR1[21]	2.727879645	2.503292951	0.934768489	3.516918609	2.358864758	1.884133863	3.486658307
ADORA1[22]	2.655434318	2.41466902	1.448549771	1.286700511	3.558002693	0.407241377	2.560765177
FOXM1[23]	2.445505348	1.299728039	1.102711393	5.289498576	0.943858655	1.65380419	2.932369532

**; genes related to lung cancer proliferation*

References

1. Shiba-Ishii et al., Mol Cancer 14, 142 (2015)
2. Jin et al., Theranostics. 2020 Jul 25;10(21):9619
3. Yuan et al., J Cancer. 2020 Jul 11;11(18):5413-5423
4. Zheng et al., Oncogene 37, 502–511 (2018)
5. Zheng et al., Front Oncol. 2020 Mar 5;10:180
6. Wang et al., Mol Carcinog. 2020 Jan;59(1):73-86
7. Kou et al., J Cancer. 2020 Feb 10;11(9):2496-2508
8. Zeng et al., Oncol Lett. 2018 Nov;16(5):5916-5922
9. Zhan et al., Mol Cancer. 2017 Jun 24;16(1):108
10. Xu et al., J Cell Physiol. 2020 Apr;235(4):3886-3893
11. Zhang et al., Front Oncol. 2021 Sep 9;11:731547
12. Tang et al., Signal Transduct Target Ther. 2020 Dec 2;5(1):279
13. Jain et al., Elife. 2020;9:e51373
14. Narimani et al., Blood Lymphat Cancer. 2019;9:53-61

15. Kaistha et al., Br J Cancer. 2014 Oct 28;111(9):1780-7
16. Xu et al., Cancer Lett. 2021 Nov 1;520:282-294
17. Li et al., Biomed Res Int. 2019 May 6;2019:6140951
18. Li et al., Int J Oncol. 2014 Oct;45(4):1547-55
19. Wang et al., Oncol Lett. 2019 Dec;18(6):5739-5746
20. Tian et al., BMC Cancer. 2018 Nov 29;18(1):1187
21. Wang et al., Biomed Pharmacother. 2019 Mar;111:588-595
22. Ni et al., Onco Targets Ther. 2020 Dec 1;13:12409-12419
23. Liang et al., Oncogene 40, 4847–4858 (2021).
