# Supplementary material for: Stratifin (SFN) regulates lung cancer progression via nucleating the Vps34‐BECN1‐TRAF6 complex for autophagy induction
Source: Clin Transl Med. 2022 Jun 8;12(6):e896. doi: 10.1002/ctm2.896 (PMC9174881; doi:10.1002/ctm2.896)
Supplement: Supplementary file 7 — Supporting information [file CTM2-12-e896-s005.pdf]

Supplementary Table S6. Up-regulated genes related to cancer migration or invasion in lung tumor tissues. (LTT, Lung Tumor Tissue; LNT, Lung Normal Tissue)

Gene	LTT26 vs. LNT26	LTT52 vs. LNT52	LTT13 vs. LNT13	LTT17 vs. LNT17	LTT51 vs. LNT51	LTT12 vs. LNT12	LTT29 vs. LNT29
ETV4[1*]	3.566510957	1.214601166	1.669677921	1.805075982	5.513216095	2.628710681	2.377141533
NUSAP1[2*]	2.619274828	2.87256555	1.858529803	1.716175731	2.296581709	0.846712133	2.614595581
MELK[3*]	2.483768726	5.266143248	2.200463478	1.425773179	2.352545292	0.909090205	2.534795851
FOXM1[4*]	2.445505348	1.299728039	1.102711393	5.289498576	0.943858655	1.65380419	2.932369532
PYCR1[5*]	2.727879645	2.503292951	0.934768489	3.516918609	2.358864758	1.884133863	3.486658307
SERINC2[6*]	3.079592099	3.012280183	1.98446753	2.735891047	2.257841708	1.027929147	2.593333095
TOP2A[7*]	3.532901932	5.278075632	2.387677714	3.440318458	3.063068467	2.112843763	3.60998915
FAM83A[8*]	3.580316098	3.519880512	4.971655964	6.113093892	1.506793975	3.186924007	6.410733001
UBE2C[9*]	4.548709059	5.160920936	2.548624652	3.380533634	2.109344831	1.223136628	3.867732138
AURKA[10*]	3.894845483	1.188626297	0.61542368	5.004398449	2.553099699	1.461907646	1.564821499
ADAM8[11]	4.349781449	3.990753976	2.571794003	2.825692227	5.942973956	2.573674428	0.445048667
PHF19[12]	8.707916533	1.102399077	1.743072395	1.923328076	2.147413807	3.734664011	2.388063636
ADORA1[13]	2.655434318	2.41466902	1.448549771	1.286700511	3.558002693	0.407241377	2.560765177
KIFC1[14]	3.782920543	1.079358514	2.204974057	3.5054001	2.685980399	1.344459512	1.973672146
ASPM[15]	4.107121954	1.923731046	2.561124806	2.683889737	0.912821893	0.012865401	2.176957302
DDX11[16]	5.119208634	0.014236098	2.423275041	0.467426435	1.341703676	0.32885802	0.617228856
CA9[17]	5.222037711	3.435790475	4.072996034	2.766525529	1.425871071	5.940020128	5.190460598

*; genes related to lung cancer migration or invasion

1. Wang et al., Mol Carcinog. 2020 Jan;59(1):73-86

2. Xu et al., J Cell Physiol. 2020 Apr;235(4):3886-3893

3. Tang et al., Signal Transduct Target Ther. 2020 Dec 2;5(1):279

4. Liang et al.,Oncogene 40, 4847–4858 (2021).

5. Sang et al., Cancer Biother Radiopharm. 2019 Aug;34(6):380-387

6. Zeng et al., Oncol Lett. 2018 Nov;16(5):5916-5922

7. Kou et al., J Cancer. 2020 Feb 10;11(9):2496-2508

8. Zheng et al., Front Oncol. 2020 Mar 5;10:180

9. Jin et al., Theranostics. 2020 Jul 25;10(21):9619

10. Zheng et al., Oncogene 37, 502–511 (2018)

11. Romagnoli et al., EMBO Mol Med. 2014 Feb;6(2):278-94

12. Jain et al., Elife. 2020;9:e51373

13. Ni et al., Onco Targets Ther. 2020 Dec 1;13:12409-12419

14. Wang et al., Oncol Lett. 2019 Dec;18(6):5739-5746

15. Wang et al., J Med Sci. 2021 Nov 6. doi: 10.1002/kjm2.12464

16. Wan et al., Ann Hepatol. 2021 Jan-Feb;20:100258

17. Chen et al., Gut. 2005 Jul;54(7):920-7
