# Supplementary material for: Stratifin (SFN) regulates lung cancer progression via nucleating the Vps34‐BECN1‐TRAF6 complex for autophagy induction
Source: Clin Transl Med. 2022 Jun 8;12(6):e896. doi: 10.1002/ctm2.896 (PMC9174881; doi:10.1002/ctm2.896)
Supplement: Supplementary file 8 — Supporting information [file CTM2-12-e896-s003.pdf]

**Supplementary Table S7. Up-regulated genes related to cancer metastasis in lung tumor tissues. (LTT, Lung Tumor Tissue; LNT, Lung Normal Tissue)**

| Gene | LTT26 vs. LNT26 | LTT52 vs. LNT52 | LTT13 vs. LNT13 | LTT17 vs. LNT17 | LTT51 vs. LNT51 | LTT12 vs. LNT12 | LTT29 vs. LNT29 |
|---|---|---|---|---|---|---|---|
| TFAP2A[1*] | 4.108089018 | 4.627790586 | 7.052621751 | 5.002351089 | 5.282324765 | 0.907654043 | 8.996207929 |
| FOXM1[2*] | 2.445505348 | 1.299728039 | 1.102711393 | 5.289498576 | 0.943858655 | 1.65380419 | 2.932369532 |
| MELK[3*] | 2.483768726 | 5.266143248 | 2.200463478 | 1.425773179 | 2.352545292 | 0.909090205 | 2.534795851 |
| NUSAP1[4*] | 2.619274828 | 2.87256555 | 1.858529803 | 1.716175731 | 2.296581709 | 0.846712133 | 2.614595581 |
| PRC1[5*] | 2.935466289 | 2.942754325 | 1.415612726 | 2.396459098 | 1.483971211 | 0.027698078 | 1.585619277 |
| ETV4[6*] | 2.784086857 | 1.625012114 | 1.097259629 | 2.250625035 | 4.802364517 | 2.230170481 | 2.706599203 |
| GALNT6[7*] | 3.84639209 | 1.370944119 | 2.42925864 | 1.668378454 | 2.662091215 | 0.89904898 | 3.690532158 |
| UBE2C[8*] | 4.548709059 | 5.160920936 | 2.548624652 | 3.380533634 | 2.109344831 | 1.223136628 | 3.867732138 |
| MMP9[9] | 7.117217255 | 0.554773509 | 4.769965 | 0.511724598 | 3.594094847 | 3.871799099 | 4.069580647 |
| ADAM8[10] | 4.349781449 | 3.990753976 | 2.571794003 | 2.825692227 | 5.942973956 | 2.573674428 | 0.445048667 |
| TROAP[11] | 3.366499071 | 1.443635655 | 0.210036474 | 1.267318886 | 1.332517794 | 1.001506831 | 2.133442549 |
| SIX4[12] | 2.356090835 | 1.904677919 | 5.700046756 | 5.091261123 | 3.122647293 | 1.617357294 | 4.395083919 |

*; genes related to lung cancer metastasis


1. Xiong et al., Cell Death Dis. 2021 Apr 6;12(4):352

2. Liang et al., Oncogene 40, 4847–4858 (2021)

3. Tang et al., Signal Transduct Target Ther. 2020 Dec 2;5(1):279

4. Xu et al., J Cell Physiol. 2020 Apr;235(4):3886-3893

5. Zhan et al., Mol Cancer. 2017 Jun 24;16(1):108

6. Wang et al., Mol Carcinog. 2020 Jan;59(1):73-86

7. Song et al., Cell Death Dis 11, 352 (2020)

8. Jin et al., Theranostics. 2019 Mar 17;9(7):2036-2055

9. Mehner et al., Oncotarget. 2014;5(9):2736-2749

10. Romagnoli et al., EMBO Mol Med. 2014 Feb;6(2):278-94

11. Li et al., Cell Death Dis 12, 125 (2021)

12. Li et al., PeerJ. 2017 May 30;5:e3394
