# Supplementary material for: Stratifin (SFN) regulates lung cancer progression via nucleating the Vps34‐BECN1‐TRAF6 complex for autophagy induction
Source: Clin Transl Med. 2022 Jun 8;12(6):e896. doi: 10.1002/ctm2.896 (PMC9174881; doi:10.1002/ctm2.896)
Supplement: Supplementary file 9 — Supporting information [file CTM2-12-e896-s007.pdf]

Supplementary Table S8. Down-regulated genes related to cancer suppressor in lung tumor tissues (LTT, Lung Tumor Tissue; LNT, Lung Normal Tissue)

Gene	LTT26 vs. LNT26	LTT52 vs. LNT52	LTT13 vs. LNT13	LTT17 vs. LNT17	LTT51 vs. LNT51	LTT12 vs. LNT12	LTT29 vs. LNT29
FCN3[1*]	-15.53550572	-14.70765367	-6.912602532	-6.556919006	-2.701738745	-6.650156822	-7.94517162
ALDH1A1[2*]	-11.15339745	-2.421959705	-1.437525147	-2.912895167	-4.252011543	-1.632470262	-1.437152718
TNNC1[3*]	-9.496628208	-4.92956863	-7.871486018	-4.157616938	-4.028635071	-5.100787719	-5.683237768
GPRC5A[4*]	-9.296498678	-1.181633477	-1.683190175	-1.959271853	-1.373161225	-6.339202492	-1.140312649
EFEMP1[5*]	-9.096071157	-2.435453001	-2.129775102	-4.234724633	-1.287842182	-5.342822722	-2.746951008
CLDN18[6*]	-8.954657688	-6.826927695	-10.2432978	-11.30237197	-5.792124635	-7.79102021	-11.59059848
SPARCL1[7*]	-8.092991261	-3.811864122	-1.67222768	-2.345521916	-3.445086171	-5.809878101	-3.18548353
TIMP3[8*]	-7.723548809	-3.233431842	-1.971615152	-0.769188323	-3.504809286	-5.509567142	-0.936067171
BMPR1A[9*]	-7.701211934	-0.065203553	-0.792567746	-0.410601652	-1.219574524	-4.886372157	-0.472721558
IRS2[10*]	-7.330024571	-0.192009922	-0.778714668	-1.568325833	-0.33722764	-5.351477952	-1.889410884
TGFBR2[11*]	-7.150905146	-1.619245384	-1.795359737	-2.018198095	-2.216208122	-4.772538328	-1.818772787
FHL1[12*]	-6.440297777	-5.945572623	-3.671703235	-4.106607705	-4.351380552	-6.626670878	-4.194343675
TCF21[13*]	-6.236978551	-6.103235648	-4.040678934	-4.31010639	-5.33977864	-7.333840235	-4.841306001
MAOA[14*]	-6.056934001	0.052678296	-1.407856289	-3.399968835	-0.869786979	-2.917981439	-2.879238968
CA4[15*]	-5.895077575	-7.191511612	-5.229630492	-6.858324019	-5.32007113	-6.906451089	-6.528139867
ING1[16*]	-5.716949327	-0.094367088	-0.176128968	-0.305188758	-0.829889921	-3.759859153	-1.211827653
UBL3[17*]	-5.63627298	-0.73651478	-0.63752097	-0.684523414	-1.233375002	-4.798123744	-1.818821557
SIK1[18*]	-5.562243713	-3.072065439	-1.460995301	-0.691918824	-2.065892215	-5.21176493	-3.929730118
CD9[19]	-8.586532342	-0.617351	-0.22516866	-2.288581983	-1.296898436	-4.330023742	-0.241281594
ABI3BP[20]	-8.312197874	-2.536619981	-3.797949201	-3.880681413	-4.446063233	-6.516260177	-4.606137154
SEPP1[21]	-8.25401476	-0.089662952	-1.292647074	-4.427269481	-1.987732963	-3.648460853	-1.938383195
PLAC8[22]	-8.106040809	-2.93055226	-0.598645824	-0.8530585	-0.111603891	-5.602964648	-5.131558594
TSPAN7[23]	-7.95488947	-3.313729776	-3.580986538	-4.921571368	-4.769883315	-5.301209747	-4.936214364
SLPI[24]	-7.553561157	-1.249221741	-1.359472533	-2.067196576	-1.326995684	-6.567641937	-1.263378546
DCN[25]	-7.344656125	-3.502195154	-0.959819807	-2.096095521	-4.789358793	-5.894809834	-1.209213851
SEPP1[26]	-7.047651363	-0.220502205	-2.101211952	-1.531861464	-1.845595475	-3.468894331	-1.494191797
HBB[27]	-6.71207092	-3.071337461	-4.527558104	-4.694668732	-3.18175744	-5.404055012	-4.255755044
GKN2[28]	-6.473420451	-5.923526152	-6.888591112	-5.730134246	-3.611186769	-9.457253465	-7.844306511
TLR7[29]	-6.384004087	-0.709770507	-0.498188128	-0.515946725	-0.421199284	-4.199616078	-2.119955257
CCL23[30]	-6.254855031	-1.299696145	-0.842176377	-3.30984615	-2.310202358	-5.675604625	-5.098213533
TGFBR3[31]	-6.167153153	-3.82839226	-3.480538151	-2.780800029	-1.636953818	-3.89553637	-5.704406903
PIK3R1[32]	-5.976405812	-5.488480727	-2.137480753	-5.230647512	-2.010135567	-3.639009446	-3.242544465
RNF8[33]	-5.810974197	-0.82757642	-0.590483176	-0.818017423	-0.440463502	-3.590041946	-0.677542814
TBC1D9[34]	-5.808659788	-1.337479914	-1.439974675	-0.922849079	-1.593191692	-4.229361696	-1.520803304

*; genes related to lung cancer suppressor

1. Jang et al., Cell Death Dis 12, 407 (2021)

2. Okudela et al., Int J Clin Exp Pathol. 2013;6(1):1-12

3. Kim et al., Mol Cells. 2020 Jul 31;43(7):619-631

4. Zhong et al., Cancer Res. 2015 May 1;75(9):1801-14

5. Lang et al., Zhongguo Fei Ai Za Zhi. 2015 Feb;18(2):92-7

6. Luo et al., Int J Cancer. 2018 Dec 15;143(12):3169-3180
7. Zhou et al., Ann Clin Lab Sci. 2021 Nov;51(6):756-765
8. Su et al., Ther Adv Med Oncol. 2019 Jul 16;11:1758835919864247
9. Xu et al., Zhongguo Fei Ai Za Zhi. 2010 Jul;13(7):659-64
10. Zhang et al., Am J Cancer Res. 2017;7(1):53-63
11. Lo et al., Cancer Lett. 2021 Mar 1;500:51-63
12. Niu et al., Int J Cancer. 2012 Jun 1;130(11):2549-56
13. Hu et al., Zhongguo Fei Ai Za Zhi. 2014 Apr;17(4):302-7
14. Huang et al., Front Oncol. 2021 Mar 8;11:645821
15. Chen et al., Oncol Lett. 2017;14(4):5046-5050.
16. Luo et al., Oncol Rep. 2011 Apr;25(4):1073-81
17. Zhao et al., Cancer Biol Med. 2020;17(1):76-87
18. Murray et al., Cancer Discov. 2019;9(11):1590-1605
19. Li et al., Cell Death Dis 11, 299 (2020)
20. Latini et al., Endocr Relat Cancer. 2008 Sep;15(3):787-99
21. Wang et al., PLoS One. 2020 Jul 31;15(7):e0236491
22. Wu et al., Oncol Lett. 2020 Nov;20(5):128
23. Yu et al., Front Oncol. 2021 Jan 8;10:613869
24. Sun et al., Int J Biol Sci. 2022;18(1):140-153
25. Hu et al., Commun Biol 4, 72 (2021)
26. Wang et al., PLoS One. 2020 Jul 31;15(7):e0236491
27. Onda et al., Br J Cancer 92, 2216–2224 (2005)
28. Ouyang et al., Am J Transl Res. 2017;9(2):803-811
29. Hyun et al., Oncotarget. 2017; 8: 24932-24948
30. Karan et al., Front Oncol. 2021;11:727583
31. Fang et al., Cancers (Basel). 2020;12(6):1375
32. Lin et al., Sci Rep 5, 8997 (2015)
33. Li et al., J Clin Invest. 2018 Oct 1;128(10):4525-4542
34. Kothari et al., Cancers (Basel). 2021 Jul 16;13(14):3557
